# C-Sulfonylation of 4-Alkylpyridines: Formal Picolyl C–H Activation via Alkylidene Dihydropyridine Intermediates

**DOI:** 10.1021/acs.joc.3c00017

**Published:** 2023-02-27

**Authors:** Soe L. Tun, Grant N. Shivers, F. Christopher Pigge

**Affiliations:** Department of Chemistry, University of Iowa, Iowa City, Iowa 52242, United States

## Abstract

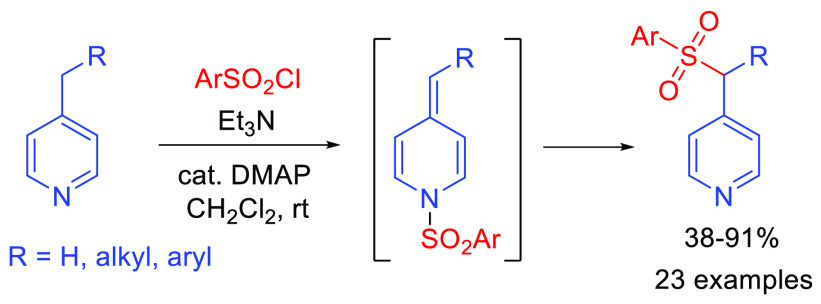

4-Picoline derivatives are converted to the corresponding aryl picolyl sulfones upon treatment with aryl sulfonyl chlorides and Et_3_N in the presence of catalytic DMAP. The reaction proceeds smoothly for a variety of alkyl and aryl picolines using a range of aryl sulfonyl chlorides. The reaction is believed to involve *N*-sulfonyl 4-alkylidene dihydropyridine intermediates and results in formal sulfonylation of unactivated picolyl C–H bonds.

Organosulfones represent an important class of functionalized organic molecules that display a wealth of useful properties. The sulfonyl group is encountered in various pharmaceuticals and bioactive molecules, agrochemicals, and functional organic materials.^1−7^ Additionally, alkyl and aryl sulfones are important synthetic intermediates in numerous preparative sequences owing to the versatile reactivity profile exhibited by sulfonyl moieties. For example, α-sulfonyl carbanions are important nucleophiles in C–C bond forming transformations, and organosulfones are promising substrates in transition metal catalyzed coupling reactions.^8−13^

Simple heterocyclic ring systems are essential components in bioactive small molecules, drugs, and natural products. Nitrogen-containing rings are particularly well-represented with structural surveys showing that ∼60% of FDA-approved small-molecule drugs possess an azaheterocyclic ring.^14,15^ Among azaheterocycles, pyridine and closely related monoaza ring systems (piperidine, quinoline, etc.) are most important, and uncovering methods that allow straightforward access to functionalized pyridine derivatives remains a prime objective of contemporary synthetic heterocyclic chemistry. Such efforts acquire added significance in that the range of commercially available pyridine building blocks is much more limited compared to carbocyclic analogues. Consequently, transformations that introduce greater molecular complexity to relatively simple pyridine substrates are especially valuable.

We are exploring new routes to functionalized alkylpyridines that are predicated upon transient generation of reactive alkylidene dihydropyridine intermediates.^16−21^ In the course of developing a direct methenylation of 4-alkylpyridines using Eschenmoser’s salt,^22^ we attempted to prepare the tosylate of hydroxypropylpyridine **1a**. However, under the conditions shown in Scheme 1 the pyridine substrate was found to undergo O-tosylation concomitantly with sulfonylation of the picolyl position, and **3aa** was isolated in good yield. Notably, related picolyl sulfonylations of 4-picoline and 4-benzylpyridines performed under similar reaction conditions have been reported previously, first by Földi and later by Anders and co-workers.^23−25^ Aside from these limited reports, it does not appear that direct sulfonylation of alkylpyridines has been examined in any detail despite the attractive reactivity profile of picolyl sulfones. Accordingly, we have investigated the utility of this transformation for functionalization of diverse alkylpyridine substrates and found that a wide range of 4-picoline derivatives can be efficiently sulfonylated in good yield under exceedingly mild reaction conditions, as described below.

**Scheme 1:**
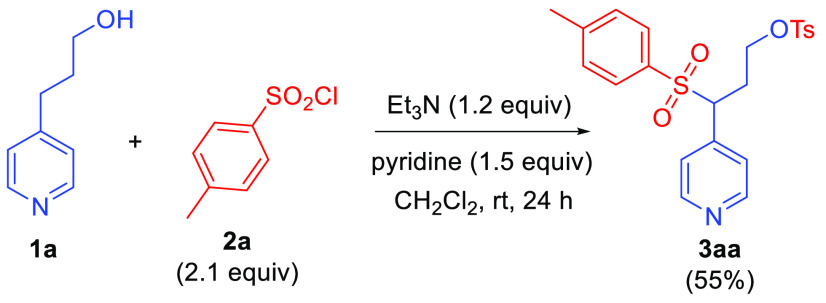
Bis(sulfonylation) of 4-Hydroxypropylpyridine

At the outset, a brief survey of reaction conditions was performed using 4-ethylpyridine (**1b**) as the test substrate in combination with tosyl chloride **2a** (Table 1). Exposing these reactants to the conditions outlined in Scheme 1 afforded the expected sulfonylated product **3ba** in good isolated yield (entry 1). Increasing the amount of **2a** and Et_3_N to 2.5 and 2.0 equiv, respectively, and omitting pyridine as an additive gave **3ba** in increased yield (entry 2). Reducing the amount of **2a** to 1.5 equiv, however, resulted in markedly decreased yield of **3ba** (entry 3). Further increasing the amount of Et_3_N to 3.5 equiv returned the best yield of **3ba** (entry 4). Including 10 mol % DMAP as a reaction additive also gave **3ba** in high yield while reducing the reaction time from 16 h to only 1.5 h (entry 5). Finally, CHCl_3_ was found to be a suitable solvent for the reaction (entry 6). Based on these results, reaction conditions shown in Table 1, entry 5 were selected to explore the scope of alkylpyridine sulfonylation.

**Table 1:**
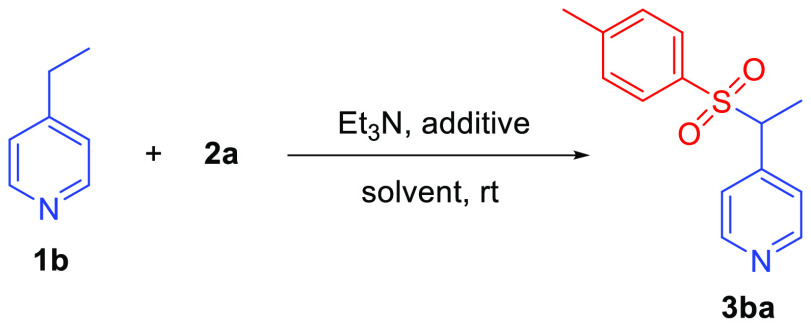
Conditions for 4-Ethylpyridine Sulfonylation^a^

entry	**2a** (equiv)	Et_3_N (equiv)	additive (equiv)	solvent	% yield **3ba**^b^
1	2.1	1.2	pyridine (1.5)	CH_2_Cl_2_	70
2	2.5	2.0	none	CH_2_Cl_2_	80
3	1.5	2.0	none	CH_2_Cl_2_	47
4	2.5	3.5	none	CH_2_Cl_2_	85
***5***	***2.5***	***3.5***	***DMAP (0.1)***	***CH**_**2**_**Cl**_**2**_*	***84***^c^
6	2.5	3.5	DMAP (0.1)	CHCl_3_	74^c^

a Reactions performed using 1.0 mmol **1b** in solvent at rt at [**1b**] = 0.4 M for 16 h.

b Isolated yield of **3ba** after purification by flash column chromatography.

c Reaction time = 1.5 h.

With identification of suitable reaction conditions, the scope of the transformation was examined using **2a** as the sulfonylating agent in combination with various 4-alkylpyridine substrates (Scheme 2). Gratifyingly, 4-alkylpyridines with relatively unactivated hydrocarbon groups were observed to give the reaction in good to excellent yield (**3ba**–**3ha**) in reaction times between 3 and 6 h. Successful substrates include 4-alkylpyridines possessing purely hydrocarbon side chains (**3ba**–**3da**) as well as tetrahydroisoquinoline (**3ea**). Several different functional groups also could be incorporated into the side chain to give more highly functionalized picolyl sulfone products. Compatible functional groups include acetoxy (**3fa**), ester (**3ga**), cyano (**3ha**), and phthalimido-protected amine (**3ia**). 4-Benzylpyridine and two additional 4-benzylpyridine derivatives were also smoothly sulfonylated under these conditions (**3ja**–**3la**). Additionally, sulfonylation of a tertiary picolyl position was successful (**3ma**), although longer reaction time was required. Notably, diaryl sulfonyl methanes have been used as organic electrophiles in Pd-catalyzed reactions with aryl boronic acids to prepare structurally diverse triarylmethanes.^26^ Several 4-methylpyridine derivatives were converted to the corresponding picolyl sulfones using slightly modified reaction conditions. Exposure of 4-picoline (**1n**) to conditions shown in Table 1, entry 5 produced significant quantities of the known bis(sulfonyl) methylpyridine in addition to desired monosulfone **3na**, as revealed in ^1^H NMR spectra of crude reaction mixtures.^25^ To avoid picolyl bis(sulfonylation), the amount of sulfonyl chloride **2a** was reduced to 2.0 equiv, and **3na** was obtained accompanied by little to no bis(sulfone) as indicated by TLC. Sulfonylation of 3-bromo-4-picoline and 4-methylquinoline also were performed using these modified conditions to afford **3oa** and **3pa**, respectively. The reaction with 2-ethylpyridine, however, was not successful, perhaps due to steric effects that interfere with initial N-sulfonylation of the substrate (*vide infra*). Additionally, 4-picolines substituted with strong electron-withdrawing groups (3-nitro and 3-cyano) were also unreactive, perhaps due to decreased pyridine nucleophilicity. Finally, this sulfonylation procedure was successfully applied in multigram scale reactions (**3ba**, **3da**, and **3na**). In these larger scale reactions sulfones **3ba** and **3na** could be conveniently isolated directly by recrystallization of crude reaction mixtures, thus avoiding the need for chromatographic purification.

**Scheme 2:**
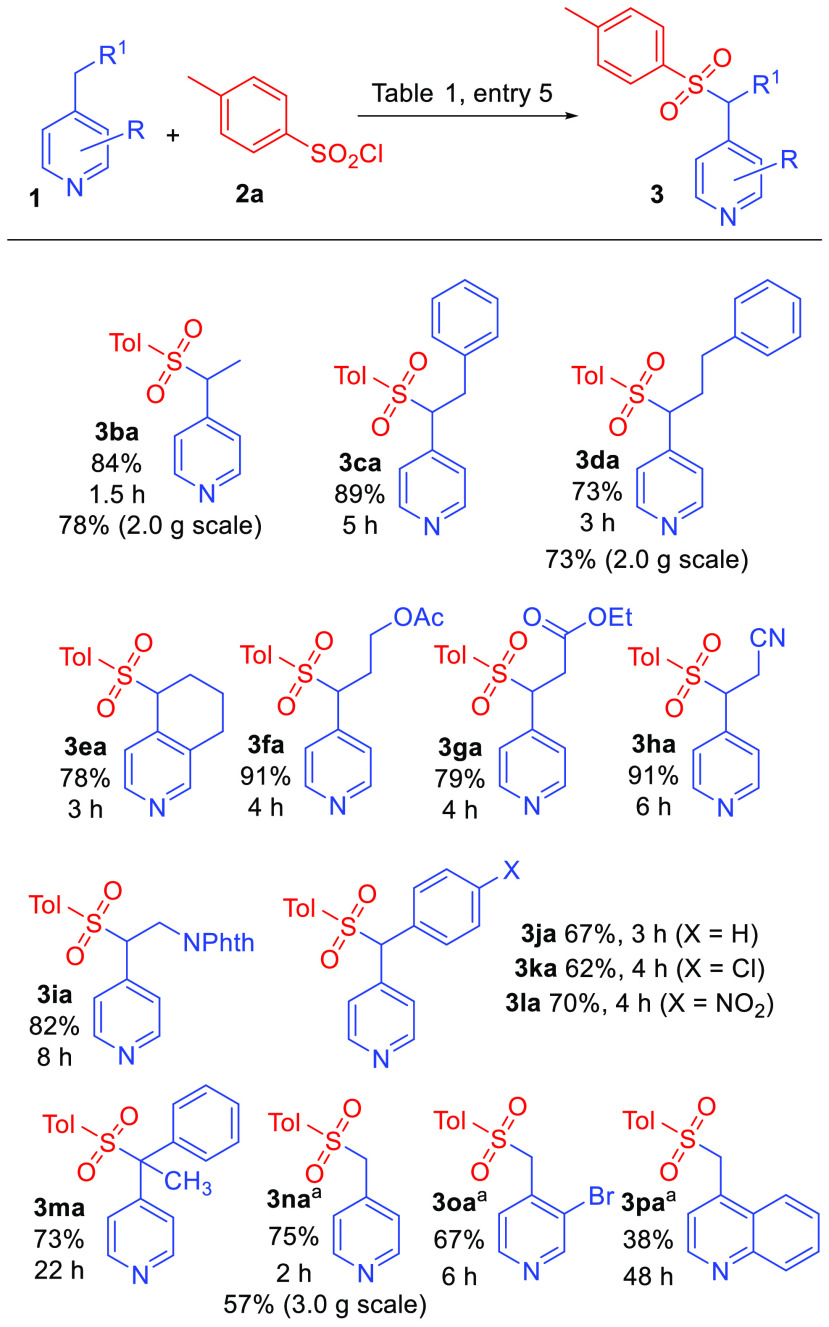
Tolylsulfonylation of 4-Alkylpyridines Reactions performed using 1.00 mmol **1** unless noted otherwise. Indicated times refer to time needed for complete disappearance of starting **1** as revealed by TLC.

The scope of this reaction with respect to the sulfonyl chloride reactant was briefly explored, and the results are shown in Scheme 3. 4-Ethylpyridine **1b** was selected as the alkylpyridine substrate for this study using reaction conditions indicated in Table 1, entry 5. Exposure of **1b** to aryl sulfonyl chlorides **2b**–**h** resulted in smooth picolyl sulfonylation in uniformly good yields in reaction times between 1.5–8 h. Unsubstituted benzene- and 1-naphthalenesulfonyl chloride were both effective sulfonylating agents (**3bb**, **3be**), along with more sterically demanding phenyl sulfonyl analogues (2,4,6-trimethyl- and 2,4,6-triisopropylphenyl sulfonyl chlorides **3bc** and **3bd**). Electron deficient nitrophenyl sulfonyl chlorides also gave the reaction in good yield (**3bf**, **3bg**), although somewhat longer reaction times were required. Finally, a heteroaromatic 2-pyridylsulfonyl chloride proved to be an excellent reaction partner, affording **3bh** in 81% yield in only 1.5 h. Attempted sulfonylation using 2-nitrobenzenesulfonyl chloride, however, was unsuccessful. Picolyl sulfonylation was also not observed when using alkyl sulfonyl chlorides (methanesulfonyl chloride and camphorsulfonyl chloride).

**Scheme 3:**
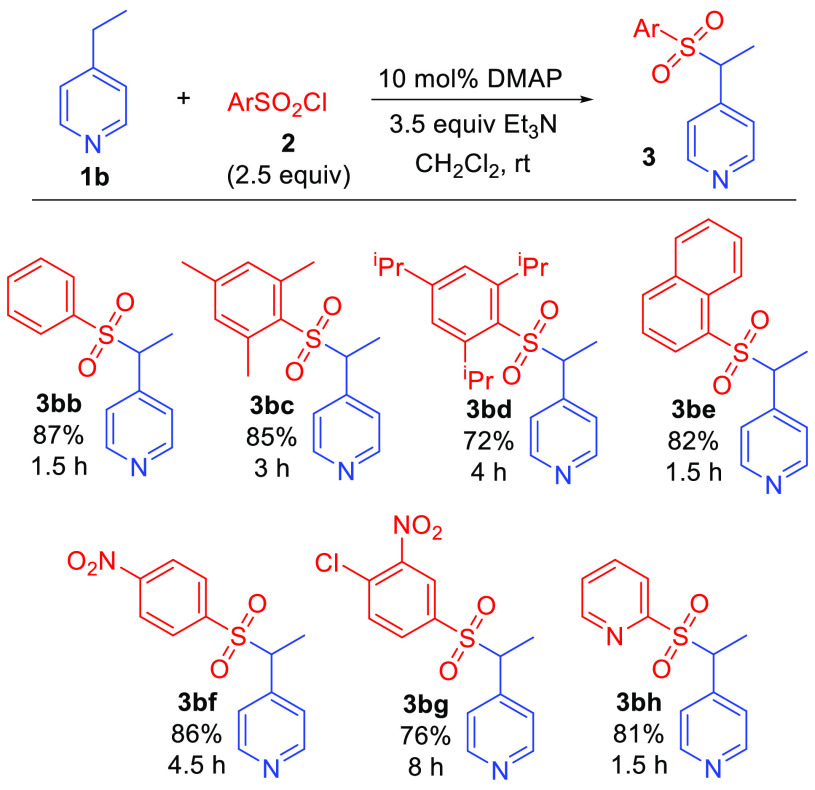
Reaction of 4-Ethylpyridine with Aryl Sulfonyl Chlorides Reactions performed using 1.00 mmol **1b**. Indicated times refer to time needed for complete disappearance of **1b** as revealed by TLC.

Considering the results depicted in Schemes 2 and 3, direct aryl sulfonylation of 4-alkylpyridines, including unactivated alkylpyridines, appears to be of broad scope and is compatible with a range of functional groups. A plausible mechanistic rationale for the transformation is illustrated in Scheme 4 and begins with initial N-sulfonylation of the pyridine substrate **4** to give pyridinium salt **5**. The picolyl position of **5** is now activated toward deprotonation, and reaction with Et_3_N affords alkylidene dihydropyridine intermediate **6**.^24,27^ Picolyl sulfonylation can then proceed in the presence of excess sulfonyl chloride activated by the addition of catalytic DMAP. Pyridinium salt **7** may then undergo N-desulfonylation to give final product **9** or undergo a second deprotonation to give alkylidene dihydropyridine **8**. Steric effects preclude additional picolyl sulfonylation (unless R = H, in which case reduced amounts of aryl sulfonyl chloride are employed), and **8** is converted to final product **9** upon addition of aqueous HCl as part of the reaction workup. The sluggish reactivity of 4-methylquinoline and the inability to sulfonylate 2-ethylpyridine under these conditions may be indicative of steric effects that impede initial formation of **5**.

**Scheme 4:**
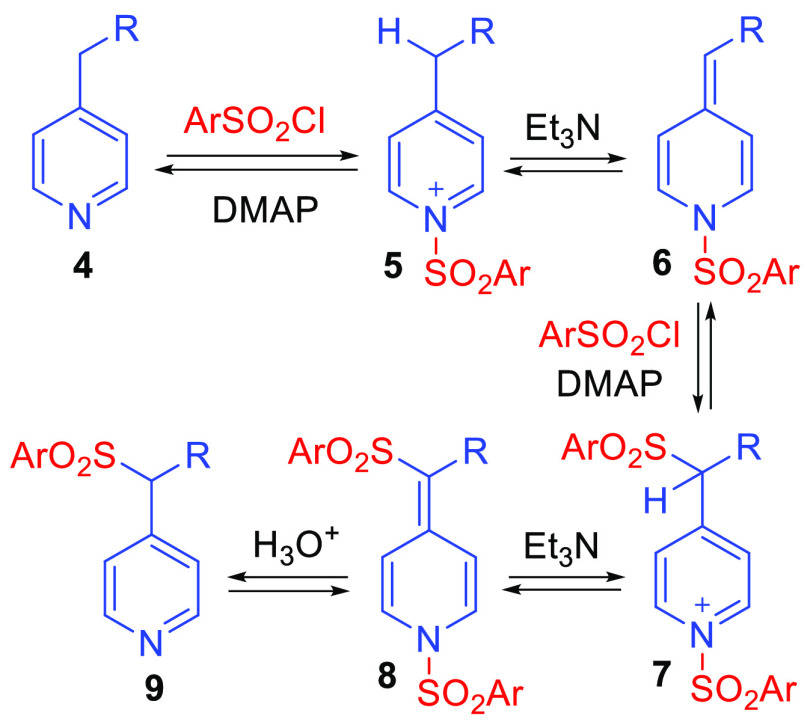
Plausible Mechanism for Picolyl Sulfonylation

The direct preparation of 4-picolyl aryl sulfones from 4-alkyl pyridines and aryl sulfonyl chlorides reported here offers straightforward access to synthetically versatile pyridine building blocks. Moreover, this method circumvents shortcomings of alternative routes to picolyl sulfones. For example, alkylation of sulfinate anions with picolyl halides is limited by the availability of sulfinate salts as well as picolyl halides.^28,29^ Likewise, oxidation of picolyl sulfides is complicated by unwanted formation of pyridine-N-oxides and requires the prior preparation of picolyl sulfides.^30^ To demonstrate the ease with which picolyl sulfones can be further manipulated, **3na** was treated with ethylene dibromide in the presence of tetrabutylammonium bromide (TBAB) and Cs_2_CO_3_ to afford cyclopropane derivative **10** in excellent yield (Scheme 5). Alkylation of **3na** with a benzyl bromide derivative using LiHMDS as a base provided **11** in good yield, and the combination of excess LiHMDS and N-fluorobenzenesulfonimide (NFSI) afforded difluorinated pyridine derivative **12** in 64% isolated yield. The sulfone moiety can also be engaged in modified Julia-type olefinations, as illustrated in the preparation of the stilbazole derivative **13** using procedures reported by Kang and co-workers.^31^ Finally, the presence of the sulfone group at the picolyl position is observed to facilitate formation of stable and isolable N-acyl alkylidene dihydropyridines, as shown in the high-yielding conversion of **3ba** to **14**. In turn, dearomatized **14** should be amenable to a variety of additional transformations, and research along these lines is in progress.

**Scheme 5:**
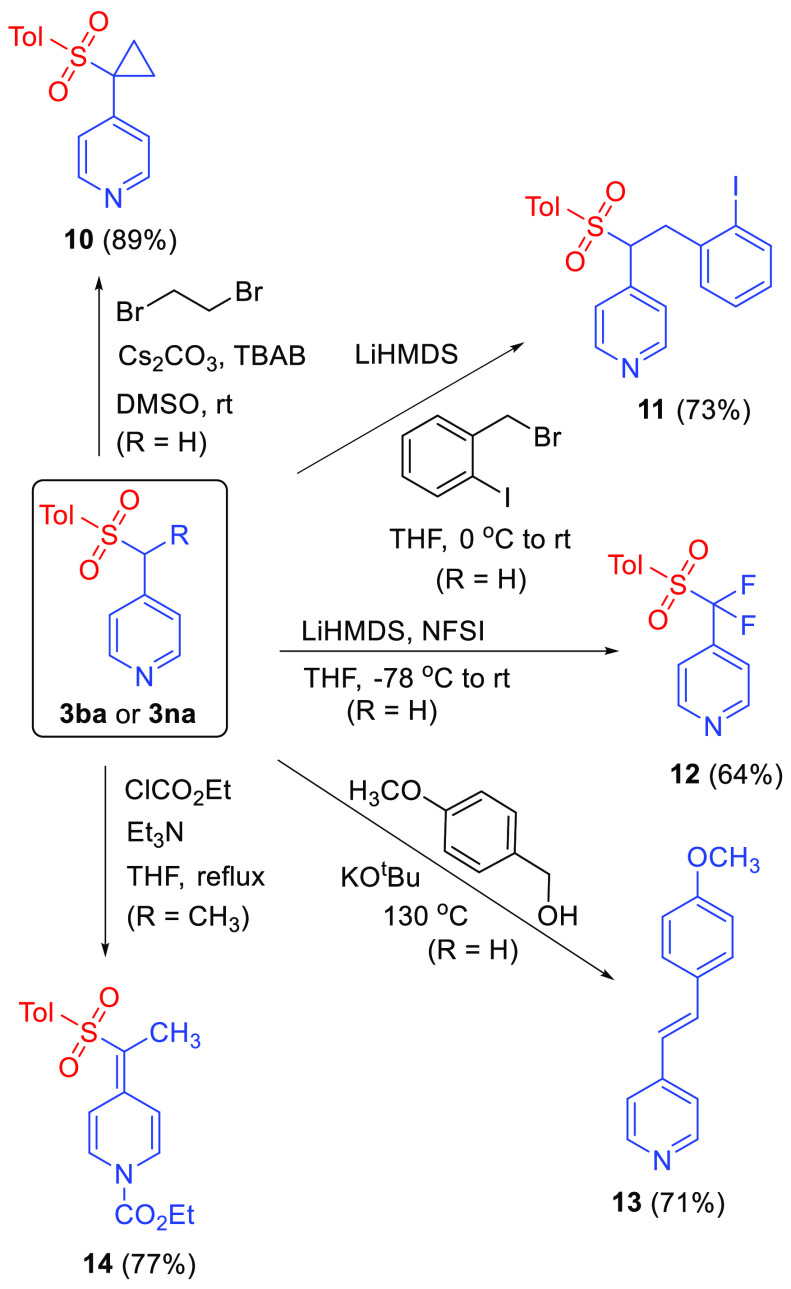
Manipulations of Picolyl Sulfones

In conclusion, an experimentally straightforward and convenient method for direct conversion of functionalized 4-alkyl pyridines to the corresponding aryl picolyl sulfones is reported. The heterocyclic sulfones accessible via this procedure possess considerable synthetic potential and may serve as valuable intermediates to a wide range of more sophisticated pyridine and pyridine-related ring systems.

## Data Availability

The data underlying this study are available in the published article and its online Supporting Information.

## Supporting Information Available

Experimental procedures, characterization data, NMR spectra (PDF)
