# Supplementary material for: C-Sulfonylation of 4-Alkylpyridines: Formal Picolyl C–H Activation via Alkylidene Dihydropyridine Intermediates
Source: J Org Chem. 2023 Feb 27;88(6):3998–4002. doi: 10.1021/acs.joc.3c00017 (PMC10028608; doi:10.1021/acs.joc.3c00017)
Supplement: Supplementary file 1 — jo3c00017_si_001.pdf [file jo3c00017_si_001.pdf]

Supporting Information for

C-Sulfonylation of 4-Alkylpyridines: Formal Picolyl C-H Activation via Alkylidene Dihydropyridine Intermediates

*Soe L. Tun, Grant. N. Shivers, and F. Christopher Pigge**

Department of Chemistry, University of Iowa, Iowa, 52242, USA

Table of Contents:

Experimental Procedures and Characterization Data S2 – S22

References S23

^{1}H and ^{13}C{^{1}H} NMR Spectra S24 – S92

Experimental Procedures and Characterization Data

All commercially available starting materials and reagents were used as received unless otherwise noted. Reactions were performed under an argon atmosphere unless otherwise noted. ^{1}H NMR spectra were recorded on an AVANCE NEO 400 spectrometer ($CDCl_3$ = 7.26 ppm or TMS= 0.00 ppm). Data are reported as follows: chemical shift in delta units (δ), multiplicity (s =singlet, d = doublet, t = triplet, q = quartet, p = pentet, doublet of doublets = dd, dt = doublet of triplets, td = triplet of doublets, qd = quartet of doublets, m = multiplet), coupling constants (reported in Hz), and integration value. ^{1}H decoupled ^{13}C NMR spectra were recorded at 100 MHz with deuterated chloroform as a standard ($CDCl_3$ = 77.16 ppm). The purity and characterization of compounds were further established by high resolution mass spectrometry (HRMS) with the Thermo Q Exactive Orbitrap mass spectrometer using positive ion electrospray ionization (ESI). Melting points were recorded using a capillary tube on a Mel-Temp apparatus and are uncorrected.

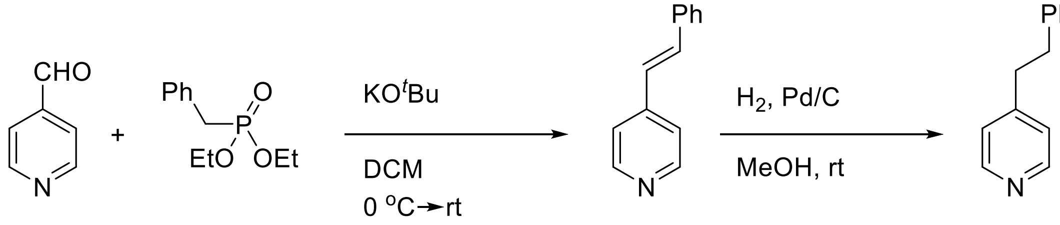

4-Phenethylpyridine (**1c**): KO^{t}Bu (1.57 g, 14.0 mmol, 1.5 equiv) was added to a round-bottomed flask containing DCM (40 mL, 0.35 M), and the flask was cooled to 0 °C. HWE reagent (2.32 mL, 11.2 mmol, 1.2 equiv) was then added and stirred for 10 minutes, followed by addition of 4-pyridinecarboxaldehyde (0.88 mL, 9.33 mmol, 1.0 equiv) dropwise to the reaction flask and let it stir overnight (~15 h). After complete consumption of the starting material as indicated by TLC, the reaction mixture was then quenched with sat. NH_4Cl (40 mL), and extracted with DCM (10 x3). The combined organic extract was then dried over Na_2SO_4, and concentrated *in vacuo*, and the crude ethyl (*E*)-4-styrylpyridine (**1c'**) was directly used without further purification for next step.

Compound **1c'** was added to a vial containing EtOH (23 mL). 10% Pd/C (0.148 g, 0.14 mmol, 0.15 equiv) was then added, and the reaction vial was purged with argon for 20 minutes, followed by purging with hydrogen balloon. The reaction mixture was then equipped with hydrogen balloon

and stirred overnight (~15 h). After consumption of the starting material as indicated by NMR, the reaction mixture was then filtered through short Celite plug, concentrated *in vacuo*, and purified by clash column chromatography using 50%-100% EtOAc in hexane to get **1c** as a white solid (0.83 g, 49%). ^{1}H NMR ($CDCl_3$, 400 MHz) δ = 8.49 (dd, J = 6.0, 1.6 Hz, 2H), 7.30-7.26 (m, 2H), 7.22-7.19 (m, 1H), 7.16-7.14 (m, 2H), 7.08 (dd, J = 6.0, 1.5 Hz, 2H), 2.93 (s, 4H). ^{13}C{^{1}H} NMR (100 MHz, $CDCl_3$) δ = 150.6, 149.9, 140.8, 128.6, 128.5, 126.4, 124.1, 37.2, 36.7. The spectral data are consistent with reported literature values.[1]

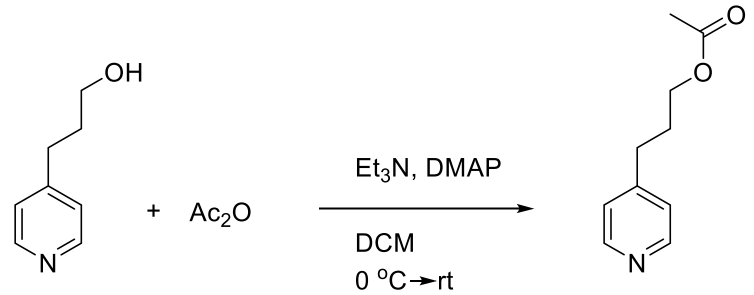

3-(Pyridin-4-yl)propyl acetate (**1f**)**:** 3-(Pyridin-4-yl)propan-1-ol (1.0 g, 7.29 mmol, 1.0 equiv), triethylamine (2 mL, 14.58 mmol, 2.0 equiv), and DMAP (89 mg, 0.73 mmol, 0.1 equiv) were added to a two-neck round bottomed flask containing DCM (16.2 mL, 0.45 M), and the reaction mixture was cooled to 0 °C. Acetic anhydride (1.03 mL, 10.94 mmol, 1.5 equiv) was added slowly and the reaction mixture was stirred at room temperature overnight (~15 h). The mixture was then extracted by DCM (5 mL x3), dried over Na_2SO_4, and concentrated *in vacuo*. The crude mixture was purified by flash column chromatography using 60-100% EtOAc in hexane to get **1f** as yellow oil (0.95 g, 73%). ^{1}H NMR ($CDCl_3$, 400 MHz) δ = 8.51 (dd, J = 6.1, 1.6 Hz, 2H), 7.12 (d, J = 6.1 Hz, 2H), 4.10 (t, J = 6.5 Hz, 2H), 2.69 (t, J = 7.8 Hz, 2H), 2.05 (s, 3H), 1.98 (m, 2H). ^{13}C{^{1}H} NMR ($CDCl_3$, 125 MHz) δ = 171.1, 150.2, 150.0, 123.9, 63.5, 31.7, 29.2, 21.0. HRMS (ESI) *m/z* calcd for $C_{10}H_{14}O_2N$ $[M+H]^+$ 180.1019, found 180.1017.

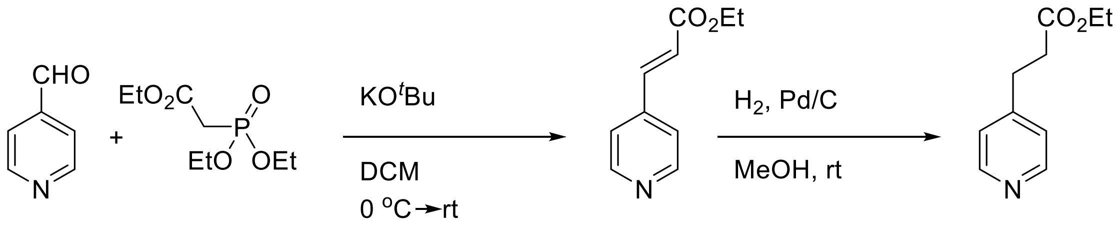

Ethyl 3-(pyridin-4-yl)propanoate (**1g**): KO^{t}Bu (1.57 g, 14.0 mmol, 1.5 equiv) was added to a

round-bottomed flask containing DCM (46 mL, 0.2 M), and the flask was cooled to 0 °C. HWE reagent (2.24 mL, 11.2 mmol, 1.2 equiv) was then added and stirred for 10 minutes, followed by addition of 4-pyridinecarboxaldehyde (0.88 mL, 9.33 mmol, 1.0 equiv) dropwise to the reaction flask and let it stir overnight (~15 h). After complete consumption of the starting material as indicated by TLC, the reaction mixture was then quenched with sat. NH_4Cl (40 mL), and extracted with DCM (10 x3). The combined organic extract was then dried over Na_2SO_4, and concentrated *in vacuo*, and the crude ethyl 3-(pyridin-4-yl)acrylate (**1g'**) was directly used without further purification for next step.

Compound **1g'** was added to a vial containing EtOH (23 mL). 10% Pd/C (0.148 g, 0.14 mmol, 0.15 equiv) was then added, and the reaction vial was purged with argon for 20 minutes, followed by purging with hydrogen balloon. The reaction mixture was then equipped with hydrogen balloon and stirred overnight (~15 h). After consumption of the starting material as indicated by NMR, the reaction mixture was then filtered through short Celite plug, concentrated *in vacuo*, and purified by flash column chromatography using 50%-100% EtOAc in hexane to get **1g** as a colorless oil (1.19 g, 71%). ^{1}H NMR ($CDCl_3$, 400 MHz) δ = 8.49 (dd, J = 6.0, 1.6 Hz, 2H), 7.12 (dd, J= 6.0, 1.5 Hz, 2H), 4.13 (q, J = 7.1 Hz, 2H), 2.95 (t, J = 7.6 Hz, 2H), 2.64 (t, J= 7.6 Hz, 2H), 1.23 (t, J= 7.1 Hz, 3H). $^{13}C\{^1H\}$ NMR (100 MHz, $CDCl_3$) δ = 172.3, 150.0, 149.5, 123.8, 60.8, 34.6, 30.2, 14.3. The spectral data are consistent with reported literature values.[1]

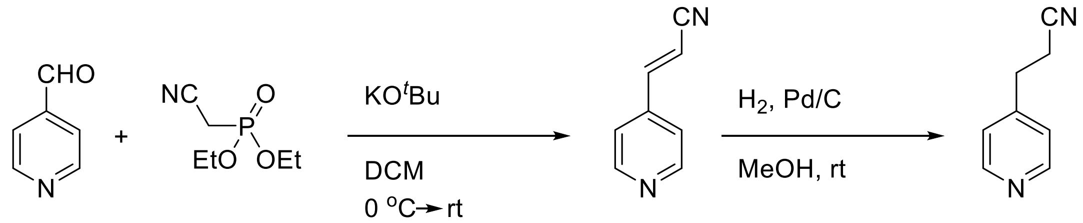

3-(Pyridin-4-yl)propanenitrile (**1h**): KO^{t}Bu (1.43 g, 12.72 mmol, 1.5 equiv) was added to a round-bottomed flask containing DCM (42 mL, 0.2 M), and the flask was cooled to 0 °C. HWE reagent (1.65 mL, 10.16 mmol, 1.0 equiv) was then added and stirred for 10 minutes, followed by addition of 4-pyridinecarboxaldehyde (0.8 mL, 8.48 mmol, 1.2 equiv) dropwise to the reaction flask and let it stir overnight (~15 h). After complete consumption of the starting material as indicated by TLC, the reaction mixture was then quenched with sat. NH_4Cl (40 mL), and extracted with DCM (10 mL x 3). The combined organic extract was then dried over Na_2SO_4, and concentrated *in vacuo*,

and the crude E-5yridinedin-4-yl)acrylonitrile (**1h'**) was directly used without further purification for next step (0.87 g, 78%).

Compound **1h'** (0.5 g, 3.8 mmol, 1.0 equiv) was added to a vial containing MeOH (0.4 M). 10% Pd/C (55 mg, 0.64 mmol, 0.15 equiv) was then added, and the reaction vial was purged with argon for 20 minutes, followed by purging with hydrogen balloon. The reaction mixture was then equipped with hydrogen balloon and stirred overnight (~15 h). After consumption of the starting material as indicated by TLC, the reaction mixture was then filtered through short Celite plug, concentrated *in vacuo*, and purified by flash column chromatography using 50%-100% EtOAc in hexane to get **1h** as a yellow oil (0.49 g, 96%). ^{1}H NMR ($CDCl_3$, 400 MHz) δ = 8.59 (d, J = 5.9 Hz, 2H), 7.18 (d, 5.9 Hz, 2H), 2.97 (t, J = 7.3 Hz, 2H), 2.67 (t, J = 7.3 Hz, 2H). $^{13}C\{^{1}H\}$ NMR ($CDCl_3$, 100 MHz) δ = 150.5, 146.7, 123.6, 118.5, 30.8, 18.3. HRMS (ESI) *m/z* calcd for $C_8H_9N_2$ $[M+H]^+$ 133.0760, found 133.0760.

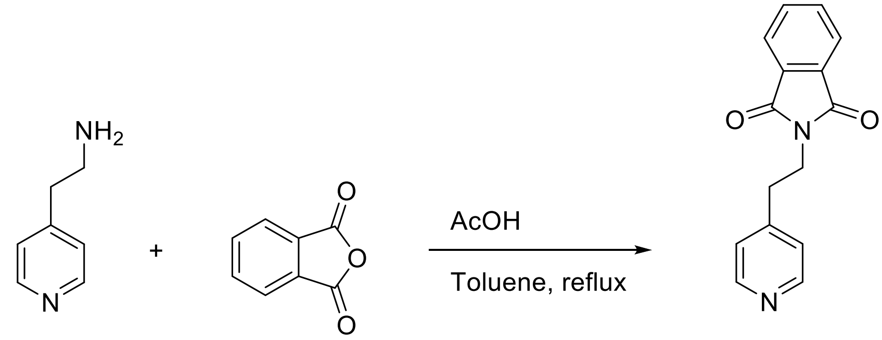

2-(2-(Pyridin-4-yl)ethyl)isoindoline-1,3-dione (**1i**): 4-(2-Aminoethyl)pyridine (1.0 g, 8.2 mmol, 1.0 equiv) was dissolved in 30 mL of toluene. Phthalic anhydride (1.57 g, 10.6 mmol, 1.3 equiv) and 1 mL of AcOH were added and the reaction was heated to reflux with a heating mantle overnight (~15 h). After cooling to room temperature, the precipitate that formed was collected by filtration and recrystallized from EtOAc to afford **1i** (1.32 g, 64%) as a white solid. ^{1}H NMR ($CDCl_3$, 400 MHz) δ = 8.51-8.50 (m, 2H), 7.84-7.81 (m, 2H), 7.72-7.70 (m, 2H), 7.19-7.17 (m, 2H), 3.98-3.94 (m, 2H), 3.03 (m, 2H). $^{13}C\{^{1}H\}$ NMR (CDCl3, 100 MHz) δ = 168.2, 150.1, 147.0, 134.2, 132.0, 124.3, 123.5, 38.1, 33.9. The spectral data are consistent with reported literature values.[1]

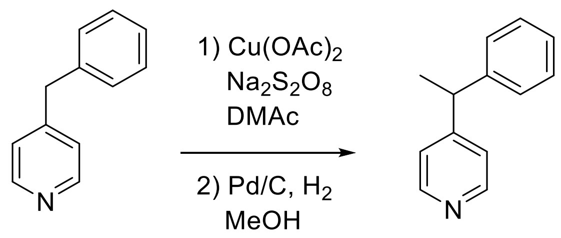

4-(1-phenylethyl)pyridine (**1m**). 4-(1-Phenylvinyl)pyridine (**1m'**) was prepared using a known procedure.[2] 4-Benzylpyridine (0.50 g, 2.96 mmol) was added to a 100 mL 2-neck flask charged with a stir bar, followed by addition of $Na_2S_2O_8$ (1.41 g, 5.91 mmol) and $Cu(OAc)_2$ (54 mg, 0.295 mmol). The compounds were dissolved in DMAc (15 mL) and sparged of oxygen by passing argon through the solution for 15 minutes. The reaction was heated to 120°C with an oil bath for 5 hours, after which time TLC indicated complete consumption of starting material. The reaction was allowed to cool to rt and ethylene diamine (3 mL), H_2O (30 mL), and EtOAc (30 mL) were added. The mixture was transferred to a separatory funnel and an additional 50 mL of H_2O and EtOAc each were added. The layers were separated, and the organic phase was washed with H_2O (3 x 50 mL). The combined organic extract was then dried over Na_2SO_4, and concentrated *in vacuo*, and the crude 4-(1-phenylvinyl)pyridine (**1m'**) was directly used without further purification for next step.

Compound **1m'** was added to a vial containing MeOH (0.4 M). 10% Pd/C (31 mg) was then added, and the reaction vial was purged with Ar for 20 minutes, followed by purging with hydrogen balloon. The reaction mixture was then equipped with hydrogen balloon and stirred overnight (~15 h). After consumption of the starting material as indicated by TLC, the reaction mixture was then filtered through short Celite plug, concentrated *in vacuo*, and purified by flash column chromatography using 25%-50% EtOAc in hexane to get **1m** as a yellow oil (0.422 g, 78% over 2 steps). ^{1}H NMR (400MHz, $CDCl_3$) δ = 8.49 (2H, dd, J = 6.1, 1.4 Hz), 7.32-7.28 (2H, m), 7.23-7.18 (3H, m), 7.13 (2H, dd, J = 6.1, 1.6 Hz), 4.13 (1H, q, J = 7.2 Hz), 1.64 (3H, d, J = 7.2 Hz); ^{13}C{^{1}H} NMR (100MHz, $CDCl_3$) δ = 155.1, 149.8, 144.4, 128.6, 127.6, 123.0, 44.2, 21.0. The spectral data are consistent with reported literature values.[3]

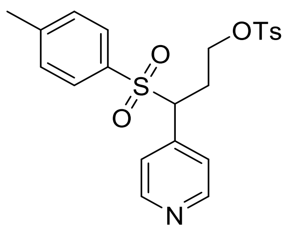

3-(Pyridin-4-yl)-3-tosylpropyl 4-methylbenzenesulfonate (**3aa**). 3-(Pyridin-4-yl)propan-1-ol **1a** (1.0g, 7.29 mmol) was added to an oven-dried round bottomed flask containing triethylamine (1.22 mL, 8.7 mmol, 1.2 equiv) and pyridine (0.9 mL, 11.1 mmol, 1.5 equiv) dissolved in dichloromethane (25 mL). Tosyl chloride (2.9 g, 15.3 mmol, 2.1 equiv) was then added and the reaction was allowed to stir at room temperature for 24 h. The mixture was then quenched with saturated Na_2CO_3 (10 mL), extracted with DCM (10 mL x4), and the combined extracts dried over anhydrous Na_2SO_4. Filtration and concentration *in vacuo* afforded a crude product that was purified using flash column chromatography using 50-70% EtOAc in hexanes as the eluent to get **3aa** as off-white solid (1.79 g, 55%). Mp 181-183 ^oC; Chromatography conditions: 30-55% EtOAc in hexanes; ^1H NMR ($CDCl_3$, 400 MHz) δ = 8.46-8.44 (m, 2H), 7.65 (d, J = 8.3 Hz, 2H), 7.41 (d, J = 8.1 Hz, 2H), 7.29 (d, J = 8.3 Hz, 2H), 7.21 (d, J = 8.1 Hz, 2H), 6.96 (d, J = 5.9 Hz, 2H), 4.17 (m, 2H), 3.75 (td, J = 9.9, 3.9 Hz, 1H), 2.81 (m, 1H), 2.44 (s, 3H), 2.41 (s, 3H), 2.39-2.32 (m, 1H); $^{13}C\{^1H\}$ NMR ($CDCl_3$, 100 MHz) δ = 150.2, 145.6, 145.4, 140.6, 133.5, 132.4, 130.1, 129.8, 129.1 , 128.0, 124.6, 66.6, 27.5, 21.8. HRMS (ESI) *m/z* calcd for $C_{22}H_{24}O_5NS_2$ $[M+H]^+$ 446.1090, found 446.1083.

General Procedure (GP1): To a solution of 4-alkylpyridine derivatives (1.0 mmol, 1.0 equiv) in 1 mL of dichloromethane in an oven-dried 25 mL round-bottomed flask was added triethylamine (3.5 mmol, 3.5 equiv) and DMAP (0.1 mmol, 0.1 equiv), then the mixture was cooled to 0 ^oC. Sulfonyl chloride (2.5 mmol, 2.5 equiv) in 3 mL of dichloromethane was added slowly, and the reaction mixture was allowed to slowly warm to room temperature and was stirred until completion as indicated by TLC. Aqueous 1M HCl (3 mL) solution was then added and the reaction maintained for additional 30 min. Saturated $NaHCO_3$ solution was then added to neutralize the solution and create an alkaline pH. The mixture was transferred to a separatory funnel, and washed with DCM (5 x 10 mL). The combined organic phase was dried over anhydrous sodium sulfate, followed by filtration and evaporation of the solvent to afford the crude product that was purified by flash column chromatography on silica gel to afford 4-picolyl sulfone derivatives.

General Procedure (GP2): To a solution of 4-picoline derivatives (1.0 mmol, 1.0 equiv) in 1 mL of dichloromethane in an oven-dried 25 mL round-bottomed flask was added triethylamine (3.0 mmol, 3.0 equiv) and DMAP (0.1 mmol, 0.1 equiv), then the mixture was cooled to 0 °C. Sulfonyl chloride (2.0 mmol, 2.0 equiv) in 3 mL of dichloromethane was added slowly, and the reaction mixture was allowed to slowly warm to room temperature and was stirred until completion as indicated by TLC. Aqueous 1M HCl (3 mL) solution was then added and the reaction maintained for additional 30 min. Saturated $NaHCO_3$ solution was then added to neutralize the solution and create an alkaline pH. The mixture was transferred to a separatory funnel and washed with DCM (5 x 10 mL). The combined organic phase was dried over anhydrous sodium sulfate, followed by filtration and evaporation of the solvent to afford the crude product that was purified by flash column chromatography on silica gel to afford 4-picolyl sulfone derivatives.

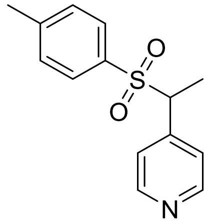

4-(1-Tosylethyl)pyridine (**3ba**): Compound **3ba** was synthesized from 4-ethylpyridine (0.11 g, 1.0 mmol) and tosyl chloride (0.44 g, 2.5 mmol) following GP1 for 1.5 h and obtained as a white solid (0.22 g, 84%). Mp 134-136 °C; Chromatography conditions: 40-70% EtOAc in hexanes; ^{1}H NMR ($CDCl_3$, 400 MHz) δ =8.51 (dd, *J*= 6.0, 1.3 Hz, 2H), 7.45 (d, *J*= 8.2 Hz, 2H), 7.23 (d, *J*= 8.2 Hz, 2H), 7.09 (dd, *J* = 6.0, 1.3 Hz, 2H), 4.19 (q, *J*= 7.1 Hz, 1H), 2.42 (s, 3H), 1.75 (d, 7.1 Hz, 3H); $^{13}C\{^1H\}$ NMR ($CDCl_3$, 100 MHz) δ = 150.0, 145.3, 143.1, 133.5, 129.7, 129.3, 124.3, 65.3, 21.8, 13.8. HRMS (ESI) *m/z* calcd for $C_{14}H_{16}O_2NS$ $[M+H]^+$ 262.0896, found 262.0894.

Gram-Scale Synthesis of 4-Picolyl Sulfone ***3ba***: To a solution of 4-ethylpyridine (2.0 g, 18.7 mmol) in 4.7 mL of DCM in an oven-dried 250 mL round-bottom flask was added DMAP (0.22 g, 1.8 mmol) and Et_3N (9.1 mL, 65.5 mmol), and the reaction mixture was cooled to 0 °C. Tosyl chloride (8.9 g, 46.7 mmol) in 42 mL of DCM was slowly added using an addition funnel over 45 minutes, and the reaction mixture was stirred at room temperature overnight (~16 h). Aqueous 1M HCl (20 mL) solution was then added, and the reaction maintained for an additional 1 h. The biphasic reaction mixture was then extracted with 1M HCl (3 x10 mL) and the combined aqueous phase

was neutralized with saturated aqueous Na_2CO_3 solution until an alkaline pH was achieved. The solid that precipitated was then collected by vacuum filtration, dissolved in DCM, and dried over anhydrous sodium sulfate. Filtration and evaporation of the solvent gave the crude product that was purified by recrystallization from EtOAc to afford 3.81 g (78%) **3ba**.

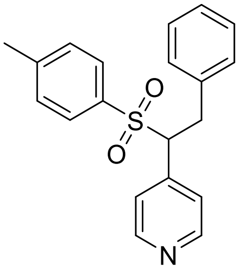

4-(2-Phenyl-1-tosylethyl)pyridine (**3ca**): Compound **3ca** was synthesized from 4-phenethylpyridine **1c** (0.18 g, 1.0 mmol) and tosyl chloride (0.47 g, 2.4 mmol) following GP1 for 5 h and obtained as a white solid (0.30 g, 89%). Mp 197-199 °C; Chromatography conditions: 25-50% EtOAc in hexanes; ^{1}H NMR ($CDCl_3$, 400 MHz) δ = 8.43 (d, J = 5.9 Hz, 2H), 7.48 (d, J = 8.2 Hz, 2H), 7.22 (d, J = 8.2 Hz, 2H), 7.13 (d, J = 6.8 Hz, 2H), 7.02 (d, J = 5.9 Hz, 2H), 6.95 (m, 2H), 4.23 (dd, J = 8.7, 3.1 Hz, 1H), 3.82 (dd, J = 14.0, 3.1 Hz, 1H), 3.34 (m, 1H), 2.40 (s, 3H); ^{13}C{^{1}H} NMR ($CDCl_3$, 100 MHz) δ = 150.0, 145.3, 141.4, 136.1, 133.9, 129.8, 129.1, 129.0, 128.8, 127.1, 124.9, 72.3, 33.9, 21.8. HRMS (ESI) *m/z* calcd for $C_{20}H_{20}O_2NS$ $[M+H]^+$ 338.1209, found 338.1205.

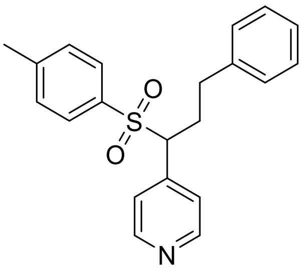

4-(3-Phenyl-1-tosylpropyl)pyridine (**3da**): Compound **3da** was synthesized from 4-(3-phenylpropyl)pyridine (0.20 g, 1.0 mmol) and tosyl chloride (0.47 g, 2.4 mmol) following GP1 for 3 h and obtained as a white solid (0.26g, 73%). Mp 121-124 °C; Chromatography conditions: 25-75% EtOAc in hexanes; ^{1}H NMR ($CDCl_3$, 400 MHz) δ = 8.53 (d, J = 5.9 Hz, 2H), 7.38 (d, J = 8.1 Hz, 2H), 7.28-7.18 (m, 5H), 7.03 (m, 4H), 3.95 (dd, J = 10.9, 3.6 Hz, 1H), 2.79-2.62 (m, 2H), 2.48-2.39 (m, 5H); ^{13}C{^{1}H} NMR ($CDCl_3$, 100 MHz) δ = 150.2, 145.2, 141.7, 139.5, 133.9, 129.7,

129.2 , 128.8, 128.5, 126.7, 124.9, 69.8, 32.5, 28.8, 21.8. HRMS (ESI) *m/z* calcd for $C_{21}H_{22}O_2NS$ $[M+H]^+$ 352.1366, found 352.1363.

Gram scale synthesis of 4-picolyl sulfone ***3da***: Using the procedure given above for the gram-scale synthesis of **3ba**, 4-(3-phenylpropyl)pyridine (2.0 g, 10.1 mmol) was treated with tosyl chloride (4.8 g, 25.4 mmol) in the presence of Et_3N (5.0 mL, 35.5 mmol) and catalytic DMAP (0.12 g, 1.0 mmol). The crude product was purified by flash column chromatography on silica gel using 25-75% EtOAc in hexane as the eluent to afford **3da** as a white solid (2.55 g, 73%).

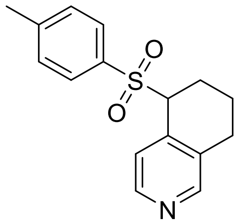

5-Tosyl-5,6,7,8-tetrahydroisoquinoline (**3ea**): Compound **3ea** was synthesized from 5,6,7,8-tetrahydroisoquinoline (0.13 g, 1.0 mmol) and tosyl chloride (0.47 g, 2.4 mmol) following GP1 for 3 h and obtained as a white solid (0.223 g, 78%). Mp 153-156 °C; Chromatography conditions: 40-70% EtOAc in hexanes; ^{1}H NMR ($CDCl_3$, 400 MHz) δ = 8.37 (m, 2H), 7.56 (d, *J* = 8.2 Hz, 2H), 7.29 (m, 3H), 4.34 (m, 1H), 2.69-2.55 (m, 2H), 2.45 (s, 3H), 2.39-2.33 (m, 1H), 2.11-2.02 (m, 2H), 1.68-1.60 (m, 1H); $^{13}C\{^1H\}$ NMR ($CDCl_3$, 100 MHz) δ = 151.0, 147.0, 145.3, 136.1, 135.0, 134.4, 129.9, 129.5, 125.6, 63.3, 25.8, 23.7, 21.8, 19.0. HRMS (ESI) *m/z* calcd for $C_{16}H_{18}O_2NS$ $[M+H]^+$ 288.1053, found 288.1048.

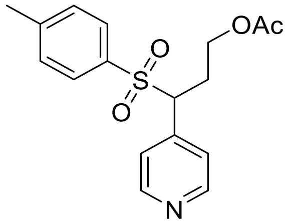

3-(Pyridin-4-yl)-3-tosylpropyl acetate (**3fa**): Compound **3fa** was synthesized from 3-(pyridin-4-yl)propyl acetate **1f** (179 mg, 1.0 mmol) and tosyl chloride (0.47 g, 2.4 mmol) following GP1 for 4 h and obtained as a white solid (0.3 g, 91%). Mp 128-130 °C; Chromatography conditions: 30-65% EtOAc in hexanes; ^{1}H NMR ($CDCl_3$, 400 MHz) δ = 8.51 (dd, *J* = 5.9, 1.3 Hz, 2H), 7.43 (d, *J*

= 8.4 Hz, 2H), 7.21 (d, *J* = 8.4 Hz, 2H), 7.04 (dd, *J* = 5.9, 1.3 Hz, 2H), 4.16 (m, 2H), 3.86 (m, 1H), 2.78 (m, 1H), 2.48-2.39 (m, 3H), 1.91 (s, 3H); $^{13}C\{^{1}H\}$ NMR ($CDCl_3$, 100 MHz) δ = 170.6, 150.2, 145.5, 141.3, 133.5, 129.8, 129.2, 124.6, 68.0, 61.1, 27.2, 21.8, 20.8. HRMS (ESI) *m/z* calcd for $C_{17}H_{20}O_4NS$ $[M+H]^+$ 334.1108, found 334.1104.

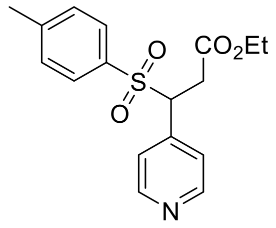

Ethyl 3-(pyridin-4-yl)-3-tosylpropanoate (**3ga**): Compound **3ga** was synthesized from ethyl 3-(pyridin-4-yl)propanoate **1g** (0.18 g, 1.0 mmol) and tosyl chloride (0.47 g, 2.4 mmol) following GP1 for 4 h and obtained as a white solid (0.26 g, 79%). Mp 169-171 °C; Chromatography conditions: 30-65% EtOAc in hexanes; ^{1}H NMR ($CDCl_3$, 400 MHz) δ = 8.51 (d, *J* = 5.9 Hz, 2H), 7.45 (d, *J* = 8.2 Hz, 2H), 7.24 (d, *J* = 8.2 Hz, 2H), 7.08 (d, *J* = 5.9 Hz, 2H), 4.59 (dd, *J* = 10.3, 4.5 Hz, 1H), 4.03 (m, 2H), 3.43 (dd, *J* = 16.7, 4.5 Hz, 1H), 3.08 (dd, *J* = 16.7, 10.3 Hz, 1H), 2.42 (s, 3H), 1.14 (t, *J* = 7.1 Hz, 3H); $^{13}C\{^{1}H\}$ NMR ($CDCl_3$, 100 MHz) δ = 169.3, 150.1, 145.7, 141.3, 133.2, 129.9, 129.3, 124.5, 66.4, 61.7, 33.2, 21.8, 14.1. HRMS (ESI) *m/z* calcd for $C_{17}H_{20}O_4NS$ $[M+H]^+$ 334.1108, found 334.1104.

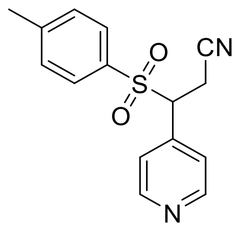

3-(Pyridin-4-yl)-3-tosylpropanenitrile (**3ha**): Compound **3ha** was synthesized from 3-(pyridin-4-yl)propanenitrile **1h** (0.13 g, 1.0 mmol) and tosyl chloride (0.47 g, 2.4 mmol) following GP1 for 6 h and obtained as a white solid (0.259 g, 91%). Mp 144-146 °C; Chromatography conditions: 30-55% EtOAc in hexanes; ^{1}H NMR ($CDCl_3$, 400 MHz) δ = 8.59 (d, *J* = 5.9, 1.5 Hz, 2H), 7.44 (d, *J* = 8.4 Hz, 2H), 7.26 (d, *J* = 8.4 Hz, 2H), 7.10 (dd, *J* = 5.9, 1.5 Hz, 2H), 4.34 (dd, *J* = 11.2, 4.8 Hz, 1H), 3.45 (dd, *J* = 17.1, 4.8 Hz, 1H), 3.22 (dd, *J* = 17.1, 11.2 Hz, 1H), 2.43 (s, 3H); $^{13}C\{^{1}H\}$

NMR ($CDCl_3$, 100 MHz) δ = 150.6, 146.5, 139.0, 132.2, 130.2, 129.3, 124.0, 115.3, 65.8, 21.9, 17.7. HRMS (ESI) *m/z* calcd for $C_{15}H_{15}O_2N_2S$ $[M+H]^+$ 287.0849, found 287.0850.

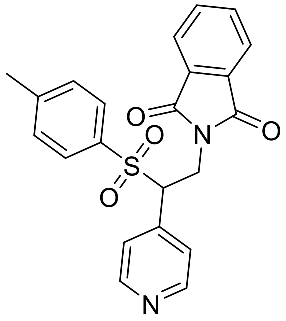

2-(2-(Pyridin-4-yl)-2-tosylethyl)isoindoline-1,3-dione (**3ia**): Compound **3ia** was synthesized from 2-(2-(pyridin-4-yl)ethyl)isoindoline-1,3-dione (0.25 g, 1.0 mmol) and tosyl chloride (0.47 g, 2.4 mmol) following GP1 for 8 h and obtained as a white solid (0.41 mg, 82%). Mp 192-195 °C; Chromatography conditions: 50-80% EtOAc in hexanes; ^1H NMR ($CDCl_3$, 400 MHz) δ = 8.47 (d, *J* = 5.9 Hz, 2H), 7.51 (m, 2H), 7.68 (m, 2H), 7.52 (d, *J* = 8.3 Hz, 2H), 7.23 (d, *J* = 8.3 Hz, 2H), 7.14 (d, *J* = 5.9 Hz, 2H), 4.97 (m, 1H), 4.5 (m, 2H), 2.39 (s, 3H); $^{13}C\{^1H\}$ NMR ($CDCl_3$, 100 MHz) δ = 167.7, 150.1, 145.8, 139.1, 134.4, 133.7, 131.6, 129.9, 129.2, 125.0, 123.7, 66.7, 36.4, 21.8. HRMS (ESI) *m/z* calcd for $C_{22}H_{19}O_4N_2S$ $[M+H]^+$ 407.1060, found 407.1061.

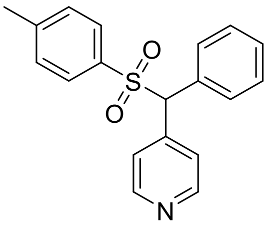

4-(Phenyl(tosyl)methyl)pyridine (**3ja**): Compound **3ja** was synthesized from 4-benzylpyridine (0.17 g, 1.0 mmol) and tosyl chloride (0.47 g, 2.4 mmol) following GP1 for 3 h and obtained as a white solid (0.22 g, 67%). Mp 205-207 °C; Chromatography conditions: 25-75% EtOAc in hexanes; ^1H NMR ($CDCl_3$, 400 MHz) δ = 8.58 (d, *J* = 5.9 Hz, 2H), 7.49 (m, 4H), 7.46-7.44 (m, 2H), 7.34-7.31 (m, 3H), 7.18 (d, *J* = 8.0 Hz, 2H), 5.22 (s, 1H), 2.38 (s, 3H); $^{13}C\{^1H\}$ NMR ($CDCl_3$, 100 MHz) δ = 150.3, 145.2, 142.2, 134.9, 131.9, 130.1, 129.6, 129.3, 129.2, 129.0, 124.7, 75.5, 21.8. HRMS (ESI) *m/z* calcd for $C_{19}H_{18}O_2NS$ $[M+H]^+$ 324.1053, found 324.1055.

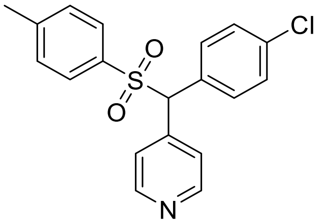

4-((4-Chlorophenyl)(tosyl)methyl)pyridine (**3ka**): Compound **3ka** was synthesized from 4-(4-chlorobenzyl)pyridine (0.20 g, 1 mmol) and tosyl chloride (0.47 g, 2.4 mmol) following GP1 for 4 h and obtained as a white solid (0.22g, 62%). Mp 219-222 °C; Chromatography conditions: 50-75% EtOAc in hexanes; ^{1}H NMR ($CDCl_3$, 400 MHz) δ = 8.59 (d, *J* = 5.7 Hz, 2H), 7.51 (d, *J* =8.2 Hz, 2H), 7.45 (d, *J* = 5.7 Hz, 2H), 7.40 (d, *J* = 8.7 Hz, 2H), 7.31 (d, *J* = 8.7 Hz, 2H), 7.21 (d, *J* = 8.2 Hz, 2H), 5.20 (s, 1H), 2.40 (s, 3H); ^{13}C{^{1}H} NMR ($CDCl_3$, 100 MHz) δ = 150.5, 145.5, 141.8, 135.6, 134.6, 131.4, 130.4, 129.8, 129.3, 129.2, 124.5, 74.7, 21.8. HRMS (ESI) *m/z* calcd for $C_{19}H_{17}O_2NClS$ [M+H]$^+$ 358.0663, found 358.0666.

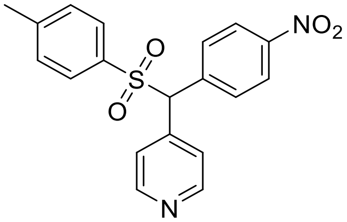

4-((4-Nitrophenyl)(tosyl)methyl)pyridine (**3la**): Compound **3la** was synthesized from 4-(4-nitrobenzyl)pyridine (0.21 g, 1.0 mmol) and tosyl chloride (0.47 g, 2.4 mmol) following GP1 for 4 h and obtained as a yellow solid (0.19 g, 70%). Mp 171-173 °C; Chromatography conditions: 30-65% EtOAc in hexanes; ^{1}H NMR ($CDCl_3$, 400 MHz) δ = 8.61 (dd, *J* = 6.0, 1.4 Hz, 2H), 8.2 (dd, *J* = 8.9, 1.9 Hz, 2H), 7.69 (dd, *J* = 8.9, 1.9 Hz, 2H), 7.52 (d, *J* = 8.5 Hz, 2H), 7.44 (dd, *J* = 6.0, 1.4 Hz, 2H), 7.22 (d, *J* = 8.5 Hz, 2H), 5.34 (s, 1H), 2.41 (s, 3H); ^{13}C{^{1}H} NMR ($CDCl_3$, 100 MHz) δ = 150.6, 148.3, 145.9, 141.0, 138.9, 134.2, 131.1, 130.0, 129.1, 124.5, 124.1, 74.7, 21.8. HRMS (ESI) *m/z* calcd for $C_{19}H_{17}O_4N_2S$ [M+H]$^+$ 369.0904, found 369.0905.

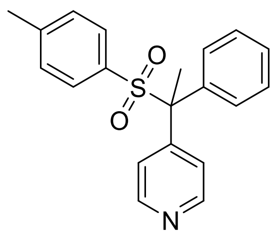

4-(1-phenyl-1-tosylethyl)pyridine (**3ma**): Compound **3ma** was synthesized from 4-(1-phenylethyl)pyridine **1m** (0.17 g, 0.91 mmol) and tosyl chloride (0.43 g, 2.26 mmol) following GP1 for 22 h and obtained as a pale-yellow solid (0.22 g, 73%). Mp 137-139 °C; Chromatography conditions: 30-55% EtOAc in hexanes; ^{1}H NMR ($CDCl_3$, 400 MHz) δ = 8.56 (d, *J* = 5.9 Hz, 2H), 7.52 (dd, *J* = 6.1, 1.4 Hz, 2H), 7.37-7.32 (m, 3H), 7.29-7.56 (m, 2H), 7.18 (d, *J* = 8.3 Hz, 2H), 7.06 (d, *J* = 8.3 Hz, 2H), 2.53 (s, 3H), 2.06 (s, 3H); ^{13}C{^{1}H} NMR ($CDCl_3$, 100 MHz) δ = 149.9, 149.4, 144.8, 137.2, 133.0, 130.5, 129.9, 129.0, 128.8, 128.3, 124.0, 74.2, 25.4, 21.7. HRMS (ESI) *m/z* calcd for $C_{20}H_{20}NO_2S$ $[M+H]^+$ 338.1209, found 338.1208.

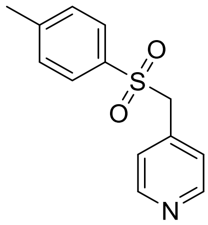

4-(Tosylmethyl)pyridine (**3na**): Compound **3na** was synthesized from 4-picoline (0.09 g, 1.0 mmol) and tosyl chloride (0.38 g, 2.0 mmol) following GP2 for 2 h and obtained as a white solid (0.18 g, 74%). Mp 181-183 °C; Chromatography conditions: 30-55% EtOAc in hexanes; ^{1}H NMR ($CDCl_3$, 400 MHz) δ = 8.53 (d, *J* = 5.9 Hz, 2H), 7.53 (d, *J* = 8.2 Hz, 2H), 7.27 (d, *J* = 8.2 Hz, 2H), 7.04 (d, *J* = 5.9 Hz, 2H), 4.27 (s, 3H), 2.43 (s, 3H); ^{13}C{^{1}H} NMR ($CDCl_3$, 100 MHz) δ = 150.2, 145.5, 137.3, 134.7, 129.9, 128.7, 125.6, 62.1, 21.8. HRMS (ESI) *m/z* calcd for $C_{13}H_{14}O_2NS$ $[M+H]^+$ 248.0740, found 248.0738.

Gram-Scale Synthesis of 4-(Tosylmethyl)pyridine ***3na**:* To a solution of 4-picoline (3.0 g, 32.2 mmol) in 8.0 mL of DCM in an oven-dried 250 mL round-bottom flask was added DMAP (0.39 g, 3.2 mmol) and Et_3N (13.4 mL, 96.6 mmol), and the reaction mixture was cooled to 0 °C. Tosyl chloride (12.2 g, 64.4 mmol) in 72 mL of DCM was slowly added using an addition funnel over

1 h, and the reaction mixture was stirred at room temperature overnight (~16 h). Aqueous 1M HCl (35 mL) solution was then added, and the reaction maintained for additional 1 h. The reaction mixture was then extracted with 1M aq. HCl (4 x 15 mL) and the combined aqueous phase was neutralized with saturated aq. Na_2CO_3 solution to create an alkaline pH. The solid that precipitated was then collected by vacuum filtration, dissolved in DCM, and dried over anhydrous sodium sulfate. Filtration and evaporation of the solvent gave the crude product that was purified by recrystallization from EtOAc to afford 4-(tosylmethyl)pyridine **3na** as a pale brown solid (4.54 g, 57%).

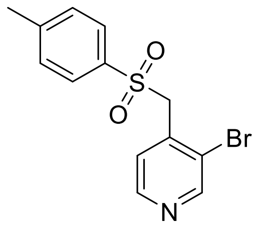

3-Bromo-4-(tosylmethyl)pyridine (**3oa**): Compound **3oa** was synthesized from 3-bromo-4-methylpyridine (0.17 g, 1.0 mmol) and tosyl chloride (0.38 g, 2.0 mmol) following GP2 for 6 h and obtained as an off-white solid (0.22 g, 67%). Mp 152-154 °C; Chromatography conditions: 10-45% EtOAc in hexanes; ^{1}H NMR ($CDCl_3$, 400 MHz) δ = 8.63 (s, 1H), 8.52 (d, *J* = 4.9 Hz, 2H), 7.55 (d, *J* = 8.3 Hz, 2H), 7.43 (d, *J* = 4.9 Hz, 2H), 7.28 (d, *J* = 8.3 Hz, 2H), 4.53 (s, 2H), 2.44 (s, 3H), ; ^{13}C{^{1}H} NMR ($CDCl_3$, 100 MHz) δ = 152.5, 148.6, 145.7, 137.3, 135.0, 130.0, 128.9, 127.1, 124.2, 60.9, 21.9. HRMS (ESI) *m/z* calcd for $C_{13}H_{13}O_2NBrS$ [M+H]$^+$ 325.9845, found 325.9852.

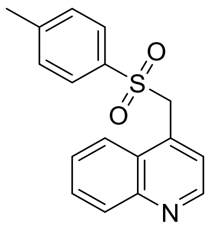

4-(Tosylmethyl)quinoline (**3pa**): Compound **3pa** was synthesized from lepidine (0.14 mg, 1.0 mmol) and tosyl chloride (0.38 g, 2.0 mmol) following GP2 for 48 h and obtained as an off-white solid (0.11 g, 38%). Mp 128-130 °C; Chromatography conditions: 20-65% EtOAc in hexanes; ^{1}H NMR ($CDCl_3$, 400 MHz) δ = 8.81 (d, *J* = 4.4 Hz, 1H), 8.11 (d, *J* = 8.4 Hz, 1H), 7.84 (d, *J* = 8.4

Hz, 1H), 7.70 (m, 1H), 7.51-7.47 (m, 3H), 7.19 (d, J = 8.1 Hz, 2H), 7.12 (d, J = 4.2 Hz, 1H), 4.79 (s, 2H), 2.39 (s, 3H); $^{13}C\{^1H\}$ NMR ($CDCl_3$, 100 MHz) δ = 149.7, 148.7, 145.4, 134.8, 134.4, 130.4, 129.9, 129.7, 128.8, 127.3, 127.2, 124.4, 123.6, 59.1, 21.8. HRMS (ESI) *m/z* calcd for $C_{17}H_{16}O_2NS$ $[M+H]^+$ 298.0896, found 298.0896.

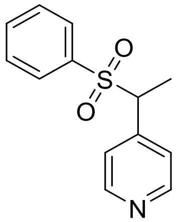

4-(1-(Phenylsulfonyl)ethyl)pyridine (**3bb**): Compound **3bb** was synthesized from 4-ethylpyridine (0.11 g, 1mmol) and benzenesulfonyl chloride (0.44 g, 2.5 mmol) following GP1 for 1.5 h and obtained as an off-white solid (0.22 g, 87%). Mp 92-93 °C; Chromatography conditions: 25-75% EtOAc in hexanes; ^1H NMR ($CDCl_3$, 400 MHz) δ = 8.51 (dd, J = 6.1, 1.5 Hz, 2H), 7.63-7.58 (m, 3H), 7.45 (m, 2H), 7.08 (dd, J = 6.1, 1.5 Hz, 2H), 4.21 (q, J = 7.1 Hz, 1H), 1.77 (d, J = 7.1 Hz, 3H); $^{13}C\{^1H\}$ NMR ($CDCl_3$, 100 MHz) δ = 150.1, 142.9, 136.4, 134.2, 129.3, 129.1, 124.3, 65.3, 13.8. HRMS (ESI) *m/z* calcd for $C_{13}H_{14}O_2NS$ $[M+H]^+$ 248.0740, found 248.0741.

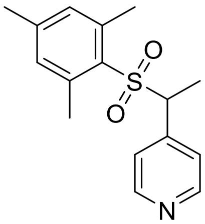

4-(1-(Mesitylsulfonyl)ethyl)pyridine (**3bc**): Compound **3bc** was synthesized from 4-ethylpyridine (0.11 g, 1mmol) and 2-mesitylenesulfonyl chloride (0.55 g, 2.5 mmol) following GP1 for 3 h and obtained as a white solid (0.25 g, 85%). Mp 162-164 °C; Chromatography conditions: 30-70% EtOAc in hexanes; ^1H NMR ($CDCl_3$, 400 MHz) δ = 8.49 (dd, J = 6.0, 1.4 Hz, 2H), 7.06 (dd, J = 6.0, 1.4 Hz, 2H), 6.89 (s, 2H), 4.26 (q, J = 7.1 Hz, 1H), 2.43 (s, 6H), 2.29 (s, 3H), 1.80 (d, J = 7.1 Hz, 3H); $^{13}C\{^1H\}$ NMR ($CDCl_3$, 100 MHz) δ = 149.9, 143.9, 142.8, 140.8, 132.3, 131.0, 124.5, 64.3, 23.0, 21.2, 12.6. HRMS (ESI) *m/z* calcd for $C_{16}H_{20}O_2NS$ $[M+H]^+$ 290.1209, found 290.1208.

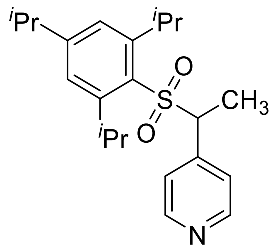

4-(1-((2,4,6-triisopropylphenyl)sulfonyl)ethyl)pyridine (**3bd**): Compound **3bd** was synthesized from 4-ethylpyridine (0.11 g, 1 mmol) and 2,4,6-triisopropylbenzenesulfonyl chloride (0.76 g, 2.5 mmol) following GP1 for 4 h and obtained as a white solid (0.27 g, 72%). Mp 112-114 ºC; Chromatography conditions: 25-75% EtOAc in hexanes; ^{1}H NMR ($CDCl_3$, 400 MHz) δ = 8.52 (dd, *J* = 6.1, 1.5 Hz, 2H), 7.11-7.10 (m, 4H), 4.27 (q, *J* = 7.1 Hz, 1H), 3.80 (br s, 2H), 2.89 (m, 1H), 1.86 (d, *J* = 7.1 Hz, 3H), 1.27-1.23 (m, 12H), 1.02 (br s, 6H); ^{13}C{^{1}H} NMR ($CDCl_3$, 100 MHz) δ = 154.3, 151.9, 150.1, 142.9, 130.5, 124.6, 124.2, 65.8, 34.3, 30.0, 23.7, 23.6, 13.0. HRMS (ESI) *m/z* calcd for $C_{22}H_{32}O_2NS$ $[M+H]^+$ 374.2148, found 374.2146.

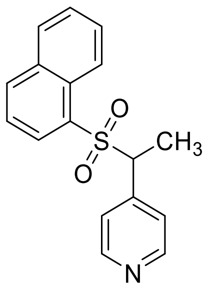

4-(1-(Naphthalen-1-ylsulfonyl)ethyl)pyridine (**3be**): Compound **3be** was synthesized from 4-ethylpyridine (0.11g, 1mmol) and 1-naphthalenesulfonylchloride (0.57 g, 2.5 mmol) following GP1 for 1.5 h and obtained as a white solid (0.24g, 82%). Mp 135-137 ºC; Chromatography conditions: 25-75% EtOAc in hexanes; ^{1}H NMR ($CDCl_3$, 400 MHz) δ = 8.74 (d, *J* = 8.2 Hz, 1H), 8.40 (dd, *J* = 6.1, 1.5 Hz, 2H), 8.08 (d, *J* = 8.4 Hz, 1H), 7.98-7.93 (m, 2H), 7.72-7.62 (m, 2H), 7.42 (t, *J* = 15.6 Hz, 1H), 6.95 (dd, *J* = 6.1, 1.5 Hz, 2H), 4.50 (q, *J* = 7.1 Hz, 1H), 1.80 (d, *J* = 7.1 Hz, 3H); ^{13}C{^{1}H} NMR ($CDCl_3$, 100 MHz) δ = 150.0, 142.9, 135.6, 134.1, 132.3, 131.9, 129.5, 129.4, 129.0, 127.2, 124.3, 124.1, 124.1, 64.7, 13.6. HRMS (ESI) *m/z* calcd for $C_{17}H_{16}O_2NS$ $[M+H]^+$ 298.0896, found 298.0893.

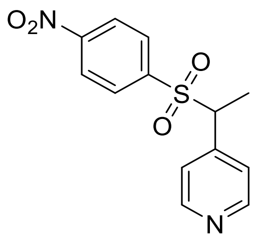

4-(1-((4-Nitrophenyl)sulfonyl)ethyl)pyridine (**3bf**): Compound **3bf** was synthesized from 4-ethylpyridine (0.11 g, 1.0 mmol) and 4-nitrobenzenesulfonyl chloride (0.55 g, 2.5 mmol) following GP1 for 4.5 h and obtained as a white solid (0.25 g, 86%). Mp 156-158 °C; Chromatography conditions: 50-75% EtOAc in hexanes; ^{1}H NMR (CDCl$_3$, 400 MHz) δ = 8.56 (d, J = 5.6 Hz, 2H), 8.29 (d, J = 8.4 Hz, 2H), 7.79 (d, J = 8.4 Hz, 2H), 7.11 (d, J = 5.6 Hz, 2H), 4.27 (q, J = 7.0 Hz, 1H), 1.82 (d, J = 7.0 Hz, 3H); ^{13}C{^{1}H} NMR (CDCl$_3$, 100 MHz) δ = 151.1, 150.5, 142.2, 142.0, 130.8, 124.2, 124.1, 65.5, 13.7. HRMS (ESI) *m/z* calcd for $C_{13}H_{13}O_4N_2S$ [M+H]$^+$ 293.0591, found 293.0589.

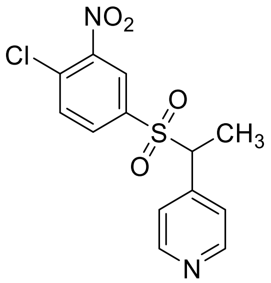

4-(1-((4-Chloro-3-nitrophenyl)sulfonyl)ethyl)pyridine (**3bg**): Compound **3bg** was synthesized from 4-ethylpyridine (0.11g, 1mmol) and 4-chloro-3-nitrobenzenesulfonyl chloride (0.64 g, 2.5 mmol) following GP1 for 8 h and obtained as a yellow solid (0.25 g, 76%). Mp 132-135 °C; Chromatography conditions: 30-65% EtOAc in hexanes; ^{1}H NMR (CDCl$_3$, 400 MHz) δ = 8.60 (d, J = 6.0 Hz, 2H), 8.13 (d, J = 1.9 Hz, 1H), 7.64-7.58 (m, 2H), 7.14 (dd, J = 6.1, 1.5 Hz, 2H), 4.27 (q, J = 7.2 Hz, 1H), 1.82 (d, J = 7.2 Hz, 3H); ^{13}C{^{1}H} NMR (CDCl$_3$, 100 MHz) δ = 150.5, 147.9, 141.8, 136.7, 133.5, 133.1, 132.9, 126.4, 124.1, 65.6, 13.7. HRMS (ESI) *m/z* calcd for $C_{13}H_{12}O_4N_2ClS$ [M+H]$^+$ 327.0201, found 327.0199.

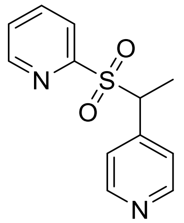

2-((1-(Pyridin-4-yl)ethyl)sulfonyl)pyridine (**3bh**): Compound **3bh** was synthesized from 4-ethylpyridine (0.11 g, 1.0 mmol) and pyridine-2-sulfonyl chloride[4] following GP1 for 1.5 h and obtained as a brown solid (0.20 g, 81%). Mp 110-113 ^{o}C; Chromatography conditions: 50-80% EtOAc in hexanes; ^{1}H NMR ($CDCl_3$, 400 MHz) δ = 8.78 (d, J = 4.5 Hz, 1H), 8.50 (d, J = 6.0 Hz, 2H), 7.85-7.79 (m, 2H), 7.53-7.51 (m, 1H), 7.20 (d, J = 6.0 Hz, 2H), 4.89 (q, J = 7.1 Hz, 1H), 1.79 (d, J = 7.1 Hz, 3H); $^{13}C\{^{1}H\}$ NMR ($CDCl_3$, 100 MHz) δ = 155.7, 150.4, 150.1, 142.5, 138.1, 127.7, 124.4, 123.8, 60.9, 13.4. HRMS (ESI) *m/z* calcd for $C_{12}H_{13}O_2N_2S$ [M+H]$^+$ 249.0692, found 249.0692.

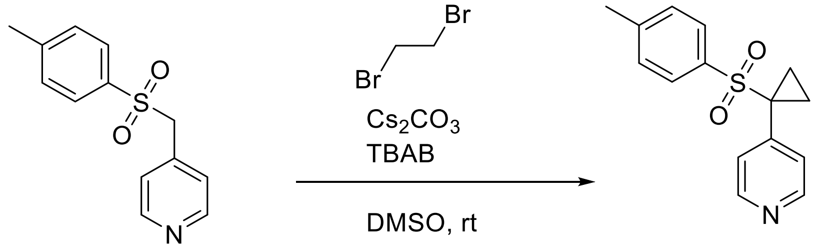

4-(1-Tosylcyclopropyl)pyridine (**10**): 1,2-Dibromoethane (0.21 mL, 2.43 mmol) was added to an oven-dried round bottomed flask containing 4-(tosylmethyl)pyridine **3na** (0.2 g, 0.81 mmol), TBATB (52 mg, 0.16 mmol), and Cs_2CO_3 (0.79 g, 2.43 mmol), and the reaction mixture was stirred at room temperature overnight. The mixture was then quenched with deionized water (10 mL), extracted with DCM (10 mL x4), and the combined extracts dried over anhydrous Na_2SO_4. Filtration and concentration *in vacuo* afforded a crude product that was purified using flash column chromatography using 2% DCM in methanol as the eluent to afford **10** as off-white solid (0.2 g, 89%). Mp 132-135 ^{o}C; ^{1}H NMR ($CDCl_3$, 400 MHz) δ = 8.47 (d, J = 5.0 Hz, 2H), 7.38 (d. J = 8.0 Hz, 2H), 7.21 (d, J = 8.0 Hz, 2H), 7.08 (d, J = 5.0 Hz, 2H), 2.42 (s, 3H), 1.99 (m, 2H), 1.26 (m, 2H); $^{13}C\{^{1}H\}$ NMR ($CDCl_3$, 100 MHz) δ = 149.9, 144.9, 143.5, 134.8, 129.6, 129.0, 126.8, 46.9, 21.8, 12.7. HRMS (ESI) *m/z* calcd for $C_{15}H_{16}O_2NS$ [M+H]$^+$ 274.0896, found 274.0894.

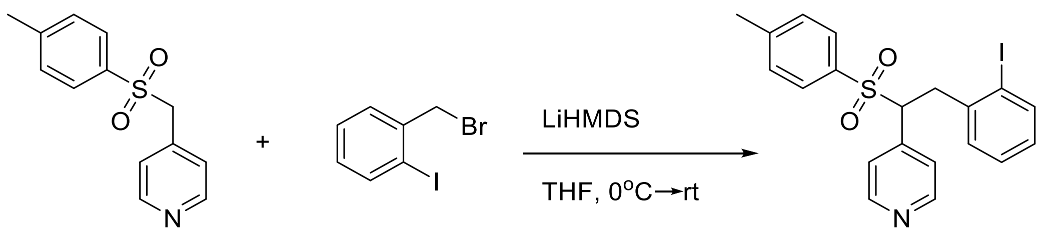

4-(2-(2-Iodophenyl)-1-tosylethyl)pyridine **(11):** 4-(Tosylmethyl)pyridine **3na** (0.3 g, 1.2 mmol) was added to an over-dried round bottomed flask containing anhydrous THF (6 mL) and the mixture was cooled to 0 °C in an ice water bath. LiHMDS (1.8 mL, 1.8 mmoL) was then added dropwise, and the reaction was allowed to stir for 1 h. 2-Iodo benzylbromide (0.43 g, 1.44 mmol) was then added, and the reaction mixture was stirred at room temperature until consumption of the starting pyridine as indicated by TLC (~3 h). The mixture was then quenched with deionized water (10 mL), extracted with DCM (10 mL x4), and the combined extracts dried over anhydrous Na_2SO_4. Filtration and concentration *in vacuo* afforded a crude product that was purified using flash column chromatography using 20% EtOAc in hexane as the eluent to afford **11** as white solid (0.41 g, 73%). Mp 109-110 °C; ^{1}H NMR ($CDCl_3$, 400 MHz) δ = 8.46 (d, J = 6.1 Hz, 2H), 7.73 (d, J = 7.7 Hz, 1H), 7.60 (d, J = 8.2 Hz, 2H), 7.26 (m, 3H), 7.10 (d, J = 6.1 Hz, 2H), 7.03 (m, 1H), 6.81 (m, 2H), 4.49 (dd, J = 11.4, 3.9 Hz, 1H), 3.82 (dd, J = 14.0, 3.9 Hz, 1H), 3.45 (dd, J = 14.0, 11.4, 1H), 2.42 (s, 3H); ^{13}C{^{1}H} NMR ($CDCl_3$, 100 MHz) δ = 150.1, 145.4, 141.0, 140.0, 138.5, 134.2, 131.2, 129.9, 129.3, 129.1, 128.4, 125.0, 100.0, 69.6, 39.2, 21.8. HRMS (ESI) *m/z* calcd for $C_{20}H_{19}O_2NIS$ $[M+H]^+$ 464.0176, found 464.0174.

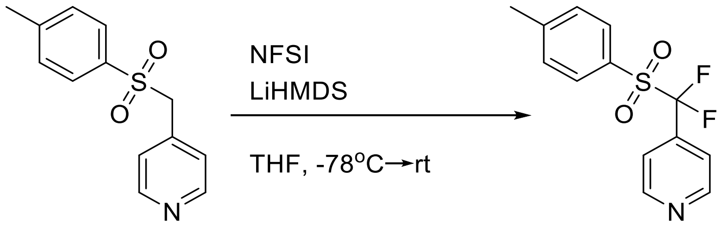

4-(Difluoro(tosyl)methyl)pyridine **(12):** 4-(Tosylmethyl)pyridine **3na** (0.2 g, 0.81 mmol, 1.0 equiv) was added to an oven-dried round bottomed flask containing anhydrous THF (4 mL, 0.2 M) and the mixture was cooled to -78 °C in an dry ice/acetone bath. LiHMDS (2.11 mL, 2.11 mmol, 2.6 equiv) was then added dropwise, and the reaction was allowed to stir for 1 h. *N*-

Fluorobenzenesulfonimide (0.77 g, 2.43 mmol, 3.0 equiv) was then added, and the reaction mixture was stirred at room temperature until consumption of the starting pyridine as indicated by TLC (~15 h). The mixture was then quenched with deionized water (10 mL), extracted with DCM (10 mL x4), and the combined extracts dried over anhydrous Na_2SO_4. Filtration and concentration *in vacuo* afforded a crude product that was purified using flash column chromatography using 10-30% EtOAc in hexane as the eluent to afford **12** as white solid (0.15 g, 64%). Mp 82-85 °C; ^{1}H NMR ($CDCl_3$, 400 MHz) δ = 8.81 (d, *J* = 5.9 Hz, 2H), 7.88 (d, *J* = 8.0 Hz, 2H), 7.56 (d, *J* = 5.9 Hz, 2H), 7.44 (d, *J* = 8.0 Hz, 2H), 2.51 (s, 3H); ^{13}C{^{1}H} NMR ($CDCl_3$, 100 MHz) δ = 150.4, 147.5, 135.5, 131.2, 130.3, 128.9, 121.9, 121.8, 121.8, 120.4, 22.1; ^{19}F NMR ($CDCl_3$, 470 MHz) δ = 104.6. HRMS (ESI) *m/z* calcd for $C_{13}H_{12}O_2NF_2S$ $[M+H]^+$ 284.0551, found 284.0550.

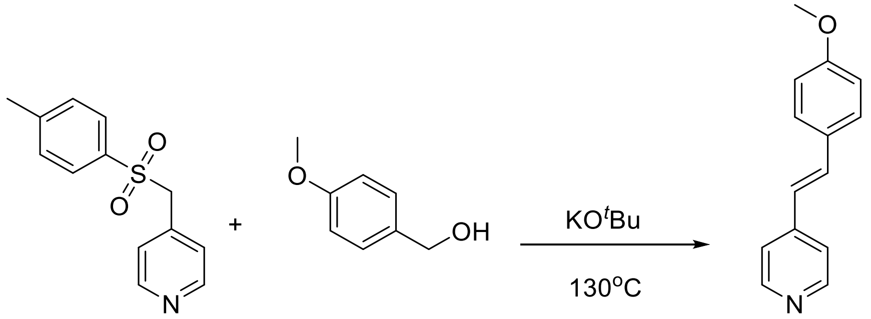

(E)-4-(4-Methoxystyryl)pyridine **(13)**[5]**:** 4-(Tosylmethyl)pyridine **3na** (0.2 g, 0.81 mmol, 1.0 equiv) was added to an oven-dried round bottomed flask containing 4-methoxybenzyl alcohol (1 mL) and the mixture was stirred at 130 °C until it became homogenous. KO^{t}Bu (0.22 g, 2.02 mmol) was then added to the flask and the reaction mixture was heated at 130 °C with an oil bath until complete consumption of the starting pyridine as indicated by TLC. After cooling, the mixture was then quenched with deionized water (5 mL), extracted with DCM (10 mL x3), and the combined extracts dried over anhydrous Na_2SO_4. Filtration and concentration *in vacuo* afforded a crude product that was purified using flash column chromatography using 20-100% EtOAc in hexanes as the eluent to get **13** as yellow solid (0.12 g, 71%). Mp 119-122 °C; ^{1}H NMR ($CDCl_3$, 400 MHz) δ = 8.55 (dd, *J* = 6.2, 1.4 Hz, 2H), 7.49 (d, *J* = 8.7 Hz, 2H), 7.34 (m, 2H), 7.26 (m, 1H), 6.92 (d, *J* = 8.7 Hz, 2H), 6.88 (d, *J* = 16.3 Hz, 1H), 3.85 (s, 3H); ^{13}C{^{1}H} NMR ($CDCl_3$, 100 MHz) δ = 160.3, 150.3, 145.1, 132.8, 129.0, 128.5, 123.9, 120.8, 114.4, 55.5. HRMS (ESI) *m/z* calcd for $C_{14}H_{14}ON$ $[M+H]^+$ 212.1070, found 212.1069.

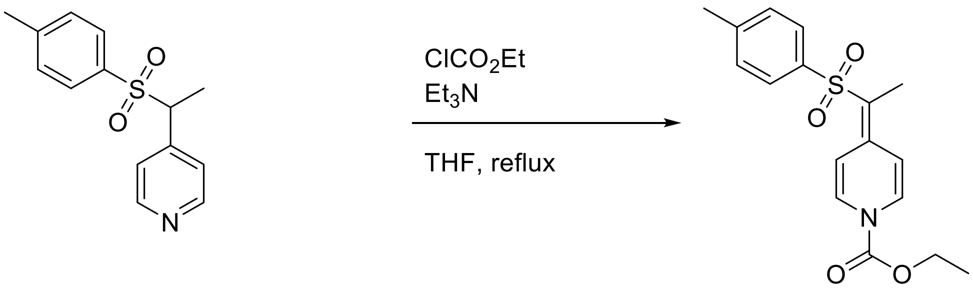

Ethyl 4-(1-tosylethylidene)pyridine-1(4H)-carboxylate **(14):** Triethylamine (1.9 mL, 13.48 mmol, 3.5 equiv) was added to a solution of 4-(1-tosylethyl)pyridine (**3ba**) (1.0 g, 3.85 mmol, 1.0 equiv) in anhydrous THF (10 mL) in a 50 mL two necked round bottomed flask. The resulting solution was stirred at reflux with an oil bath for 5 minutes and ethyl chloroformate (1.0 mL, 9.55 mmol, 2.5 equiv) was added. After completion of anhydrobase formation in 40 minutes, as indicated by NMR, reaction mixture was allowed to cool to room temperature and filtered through short basic alumina pad, concentrated *in vacuo*, and purified by recrystallization using EtOAc to afford ethyl 4-(1- tosylethylidene)pyridine-1(4H)-carboxylate **14** as white solid (0.98 g, 77%). Mp 126-129 °C; ^{1}H NMR ($CDCl_3$, 400 MHz) δ = 7.74 (d, *J* = 8.2 Hz, 2H), 7.56 (dd, *J* = 8.5, 2.4 Hz, 1H), 7.41 (d, *J* = 8.2 Hz, 1H), 7.30 (m ,3H), 6.01 (dd, *J* = 8.5, 2.4 Hz, 1H), 4.39 (q, *J* = 7.1 Hz, 2H), 2.40 (s, 3H), 1.98 (s, 3H), 1.39 (t, *J* = 7.1 Hz, 3H); ^{13}C{^{1}H} NMR ($CDCl_3$, 100 MHz) δ = 150.4, 143.2, 140.1, 136.8, 129.7, 128.4, 126.5, 126.5, 113.0, 110.5, 110.3, 64.6, 21.7, 14.5, 14.4. HRMS (ESI) *m/z* calcd for $C_{17}H_{20}O_4NS$ $[M+H]^+$ 334.1108, found 334.1104.

References

1. Wasfy, N.; Rasheed, F.; Robidas, R.; Hunter, I.; Shi, J.; Doan, B.; Legault, C. Y.; Fishlock, D.; Orellana, A., *Chem. Sci.* **2021**, *12* (4), 1503-1512.

2. Itoh, M.; Hirano, K.; Satoh, T.; Miura, M., *Org. Lett.* **2014**, *16* (7), 2050-2053.

3. Nakao, Y.; Yamada, Y.; Kashihara, N.; Hiyama, T., *J. Am. Chem. Soc.* **2010**, *132* (39), 13666-13668.

4. Nacsa, E. D.; Lambert, T. H., *Org. Chem. Front.* **2018**, *5* (1), 64-69.

5. Yao, C.-Z.; Li, Q.-Q.; Yang, M.-M.; Ning, X.-S.; Kang, Y.-B., *Chem. Commun.* **2015**, *51* (36), 7729-7732.

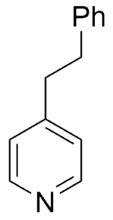

1c

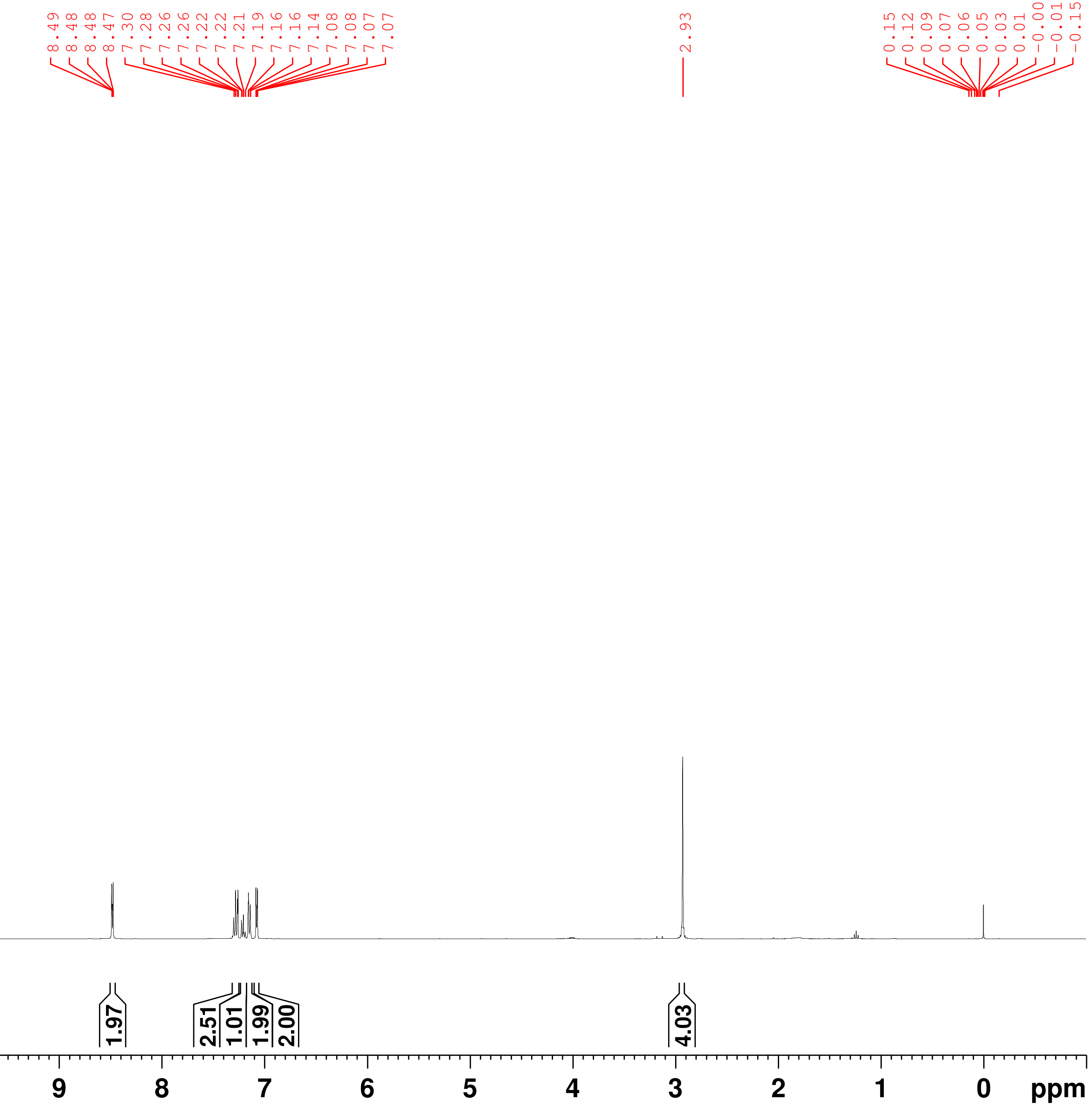

Current Data Parameters
NAME 1H_ST-7-219
EXPNO 4
PROCNO 1

F2 - Acquisition Parameters
Date_ 20220521
Time 10.47 h
INSTRUM Avance
PROBHD Z167430_0032 (
PULPROG zg30
TD 65536
SOLVENT CDCl3
NS 16
DS 0
SWH 8196.722 Hz
FIDRES 0.250144 Hz
AQ 3.9976959 sec
RG 101
DW 61.000 usec
DE 13.20 usec
TE 298.0 K
D1 0.10000000 sec
TD0 1
SFO1 400.3024719 MHz
NUC1 1H
P0 4.00 usec
P1 12.00 usec
PLW1 8.80000019 W

F2 - Processing parameters
SI 65536
SF 400.3000104 MHz
WDW EM
SSB 0
LB 0.30 Hz
GB 0
PC 1.00

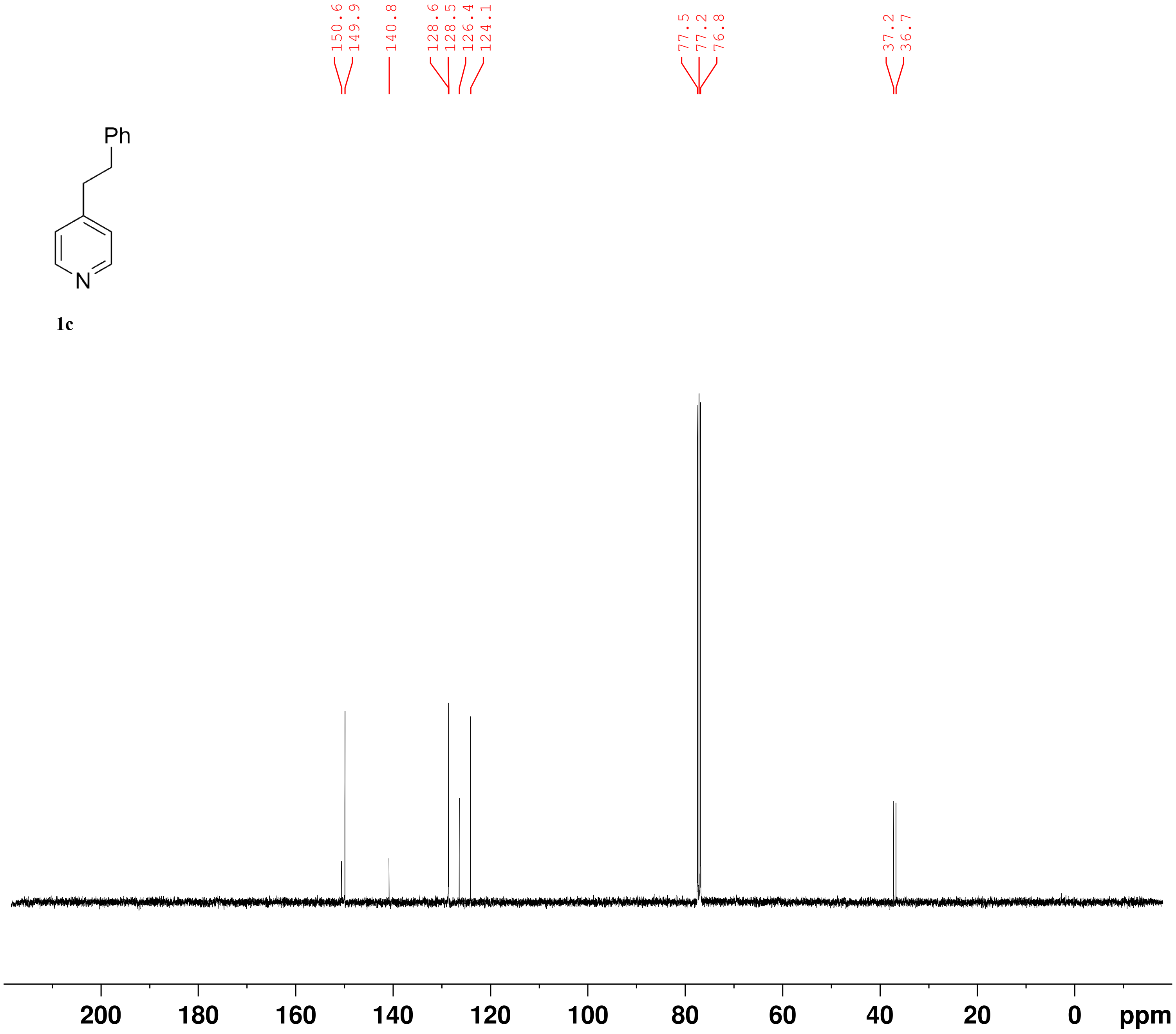


```
Current Data Parameters
NAME          13C_ST-7-219
EXPNO                    2
PROCNO                   1

F2 - Acquisition Parameters
Date_             20220521
Time                 10.53 h
INSTRUM             Avance
PROBHD     Z167430_0032 (
PULPROG            zgpg30
TD                   65536
SOLVENT              CDCl3
NS                      32
DS                       4
SWH              23809.523 Hz
FIDRES            0.726609 Hz
AQ               1.3762560 sec
RG                    3.25
DW                  21.000 usec
DE                   19.29 usec
TE                   298.0 K
D1              2.00000000 sec
D11             0.03000000 sec
TD0                      1
SFO1           100.6655806 MHz
NUC1                   13C
P0                    3.33 usec
P1                   10.00 usec
PLW1           39.31399918 W
SFO2           400.3016012 MHz
NUC2                    1H
CPDPRG[2           waltz64
PCPD2                80.00 usec
PLW2            8.80000019 W
PLW12           0.20176961 W
PLW13           0.10112690 W

F2 - Processing parameters
SI                  131072
SF             100.6555034 MHz
WDW                     EM
SSB      0
LB                    1.00 Hz
GB       0
PC                    1.40
```

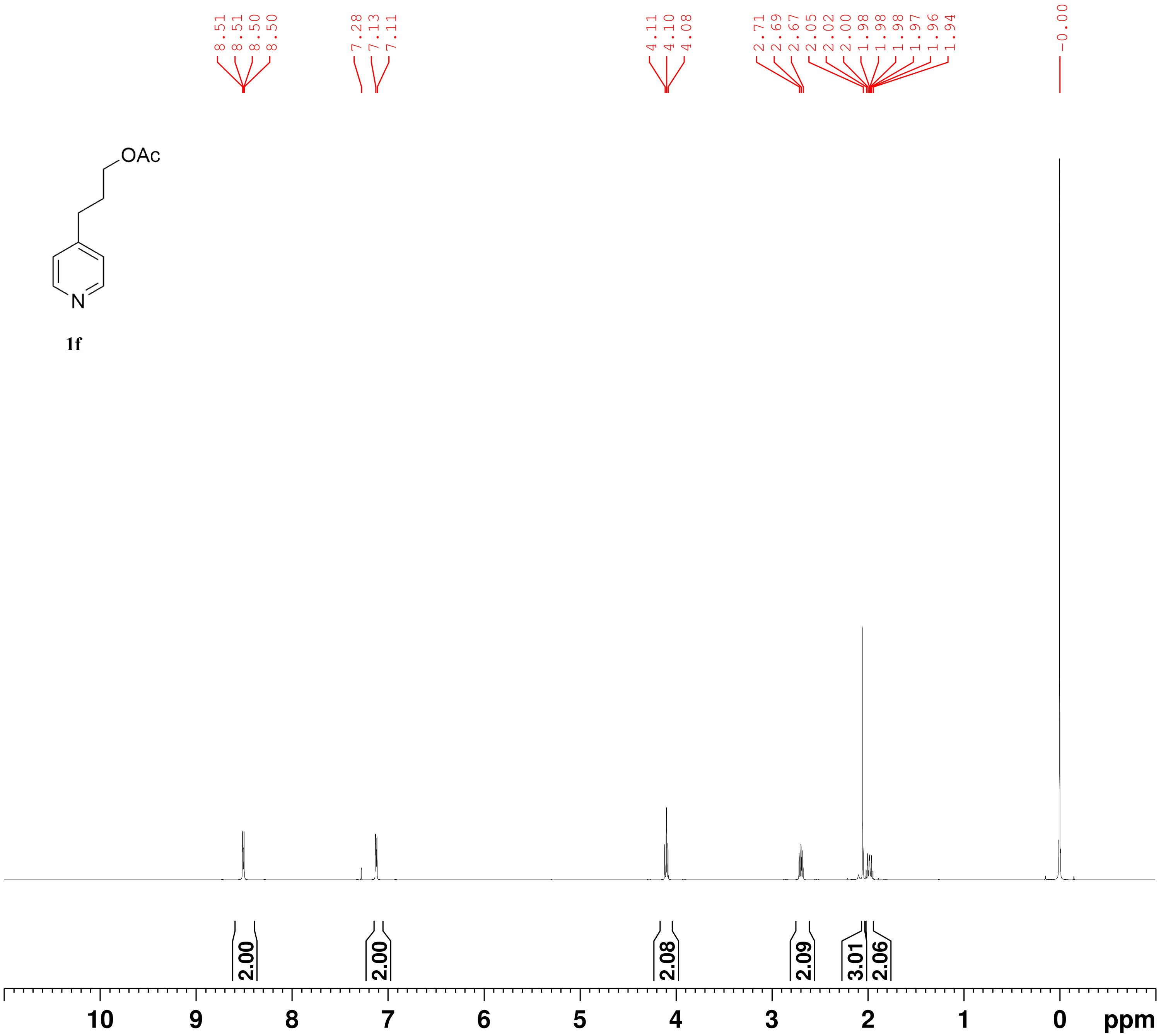


```
Current Data Parameters
NAME        1H gns-15-050
EXPNO                   1
PROCNO                  1

F2 - Acquisition Parameters
Date_            20220209
Time                18.47 h
INSTRUM            Avance
PROBHD     Z167430_0032 (
PULPROG              zg30
TD                  65536
SOLVENT             CDCl3
NS                     16
DS                      0
SWH              8196.722 Hz
FIDRES           0.250144 Hz
AQ              3.9976959 sec
RG                86.8817
DW                 61.000 usec
DE                  13.20 usec
TE                  298.0 K
D1             0.10000000 sec
TD0                     1
SFO1          400.3024719 MHz
NUC1                   1H
P0                   4.00 usec
P1                  12.00 usec
PLW1           8.80000019 W

F2 - Processing parameters
SI                  65536
SF            400.3000024 MHz
WDW                    EM
SSB      0
LB                   0.30 Hz
GB       0
PC                   1.00
```

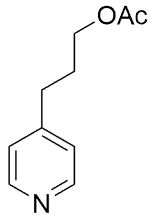

1f

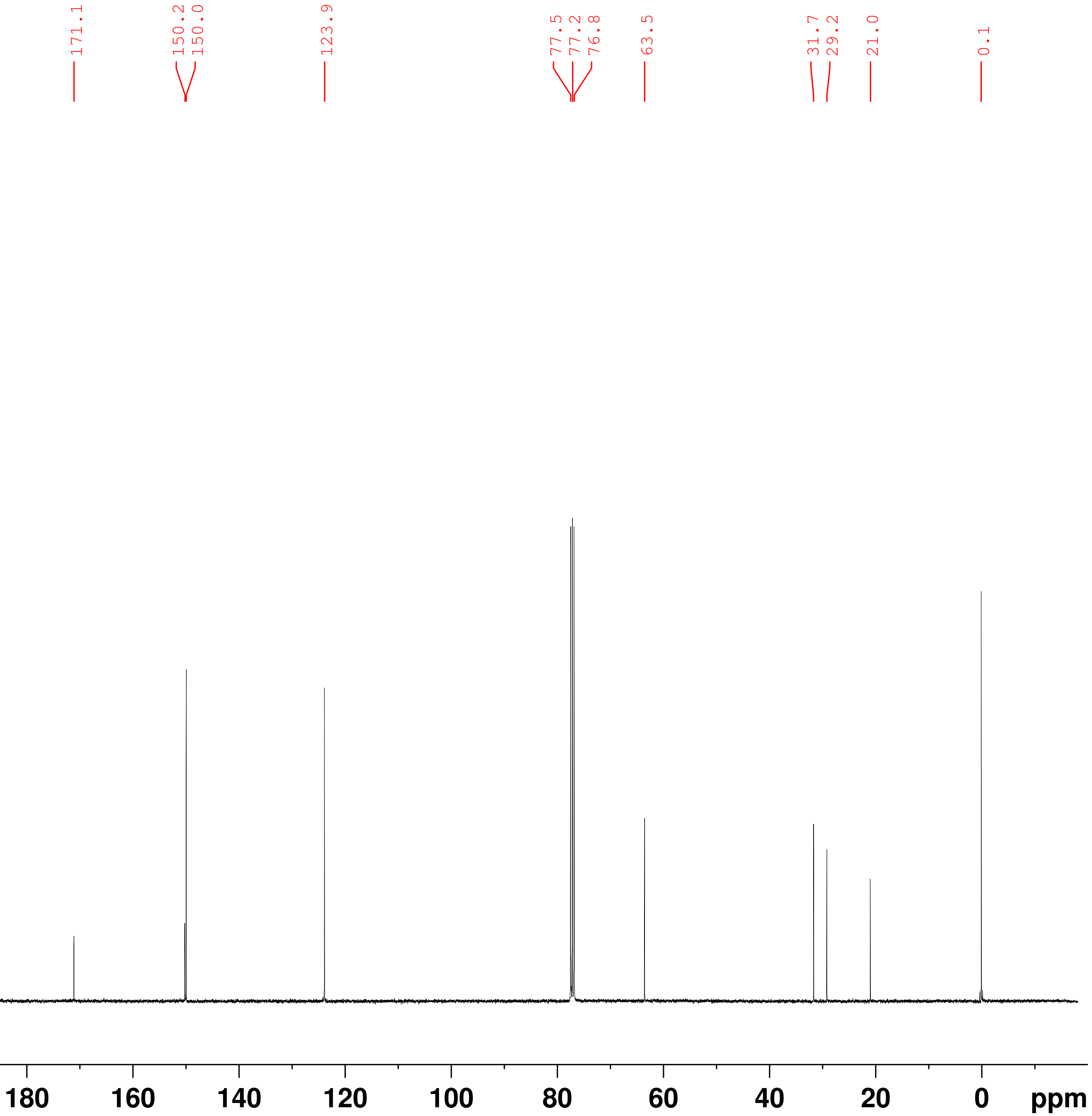


```
Current Data Parameters
NAME      13C gns-15-050
EXPNO                  2
PROCNO                 1

F2 - Acquisition Parameters
Date_           20220209
Time               18.55 h
INSTRUM           Avance
PROBHD   Z167430_0032 (
PULPROG          zgpg30
TD                 65536
SOLVENT            CDCl3
NS                   100
DS                     4
SWH            23809.523 Hz
FIDRES          0.726609 Hz
AQ             1.3762560 sec
RG                  3.25
DW                21.000 usec
DE                 19.29 usec
TE                 298.0 K
D1            2.00000000 sec
D11           0.03000000 sec
TD0                    1
SFO1         100.6655806 MHz
NUC1                 13C
P0                  3.33 usec
P1                 10.00 usec
PLW1         39.31399918 W
SFO2         400.3016012 MHz
NUC2                  1H
CPDPRG[2         waltz64
PCPD2              80.00 usec
PLW2          8.80000019 W
PLW12         0.20176961 W
PLW13         0.10112690 W

F2 - Processing parameters
SI                 65536
SF           100.6555041 MHz
WDW                   EM
SSB      0
LB                  1.00 Hz
GB       0
PC                  1.40
```

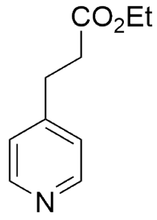

1g

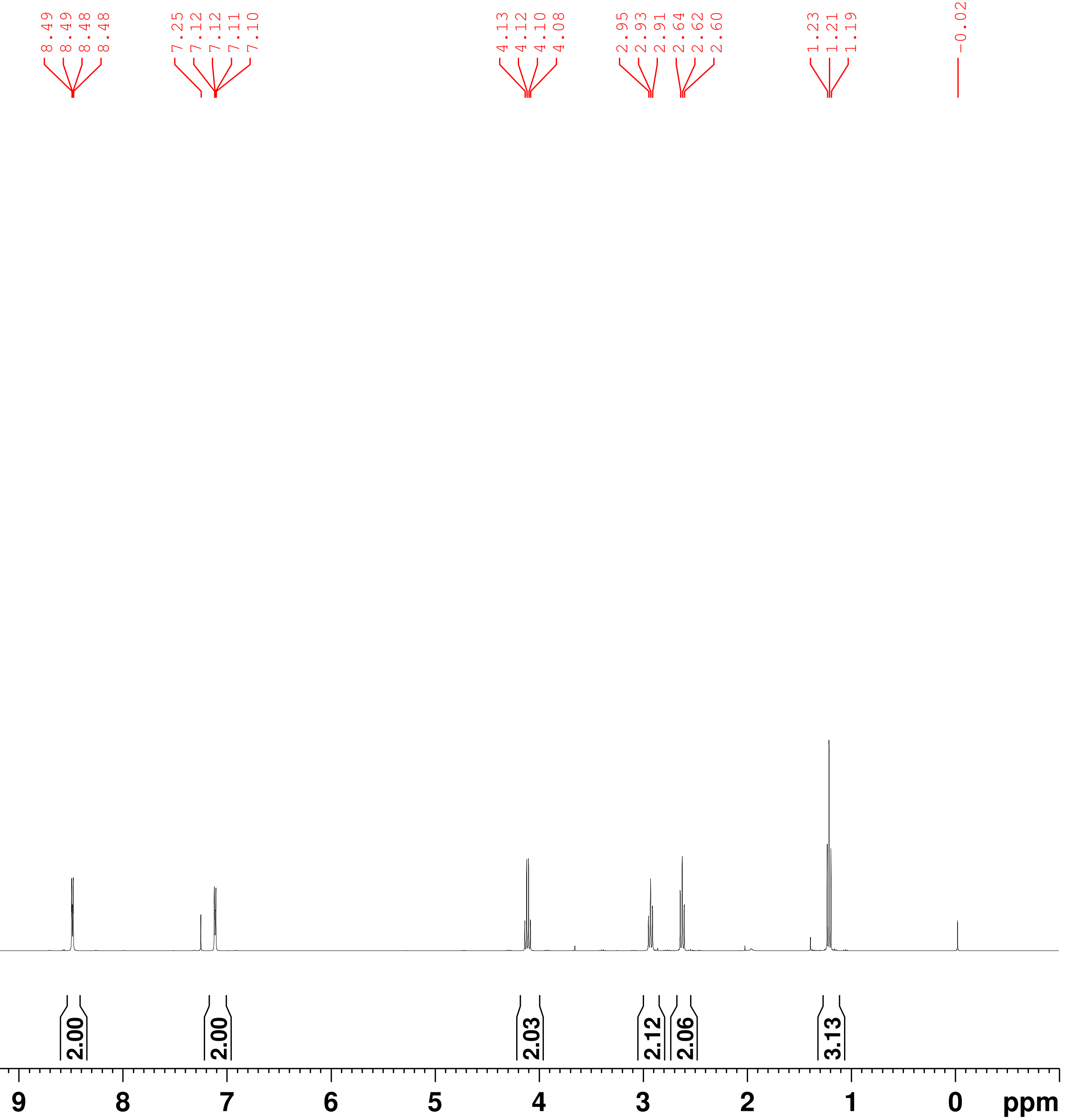


```
Current Data Parameters
NAME         1H ST-07-073
EXPNO                   4
PROCNO                  1

F2 - Acquisition Parameters
Date_            20220303
Time                 9.45 h
INSTRUM            Avance
PROBHD   Z167430_0032 (
PULPROG              zg30
TD                  65536
SOLVENT             CDCl3
NS                     16
DS                      0
SWH              8196.722 Hz
FIDRES           0.250144 Hz
AQ              3.9976959 sec
RG                86.8817
DW                 61.000 usec
DE                  13.20 usec
TE                  298.0 K
D1             0.10000000 sec
TD0                     1
SFO1          400.3024719 MHz
NUC1                   1H
P0                   4.00 usec
P1                  12.00 usec
PLW1           8.80000019 W

F2 - Processing parameters
SI                  65536
SF            400.3000139 MHz
WDW                    EM
SSB      0
LB                   0.30 Hz
GB       0
PC                   1.00
```

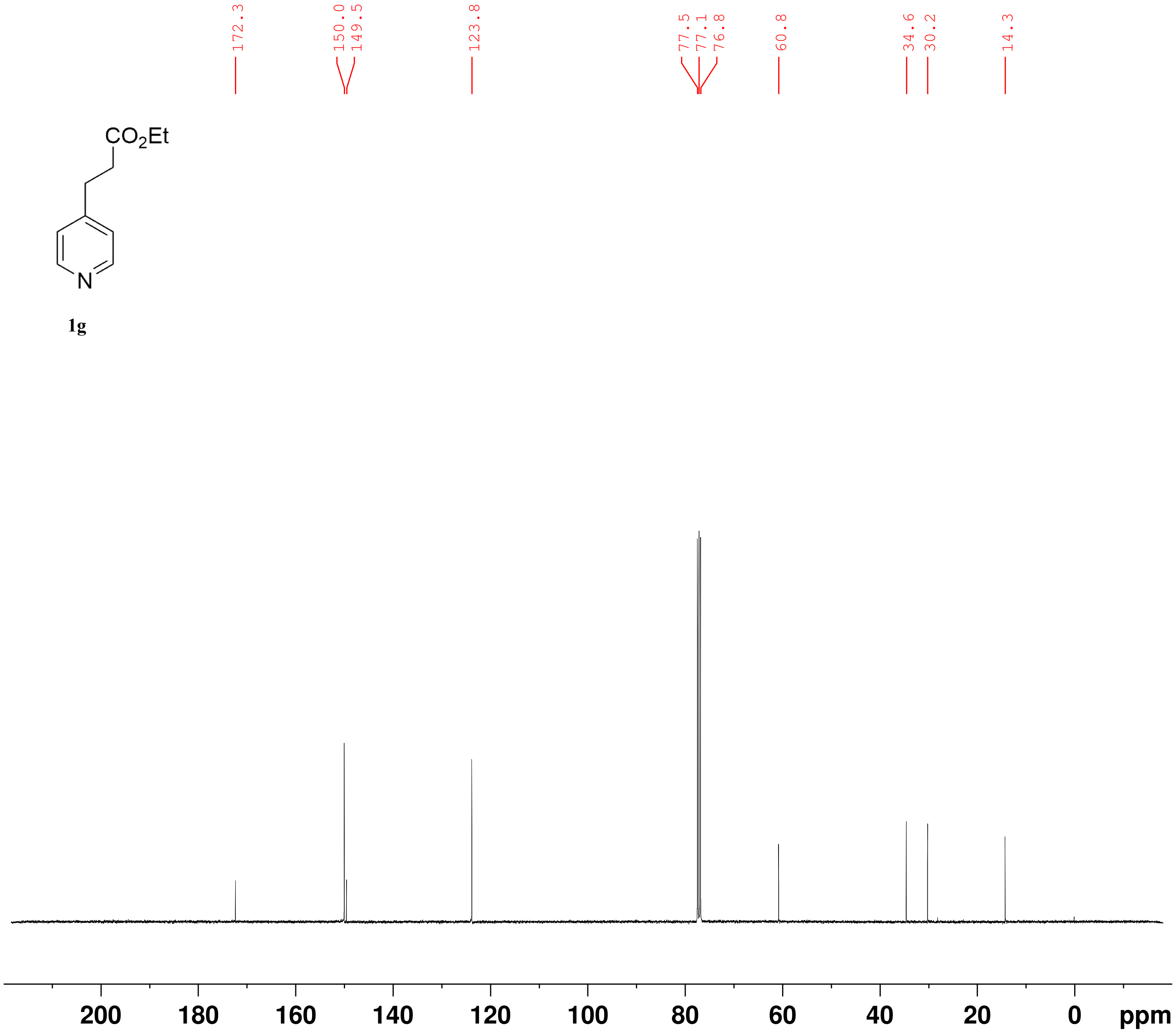

Current Data Parameters

NAME	13C ST-07-073	
EXPNO	5	
PROCNO	1	

F2 - Acquisition Parameters

Date_	20220303	
Time	9.53	h
INSTRUM	Avance	
PROBHD	Z167430_0032 (	
PULPROG	zgpg30	
TD	65536	
SOLVENT	CDCl3	
NS	100	
DS	4	
SWH	23809.523	Hz
FIDRES	0.726609	Hz
AQ	1.3762560	sec
RG	3.25	
DW	21.000	usec
DE	19.29	usec
TE	298.0	K
D1	2.00000000	sec
D11	0.03000000	sec
TD0	1	
SFO1	100.6655806	MHz
NUC1	13C	
P0	3.33	usec
P1	10.00	usec
PLW1	39.31399918	W
SFO2	400.3016012	MHz
NUC2	1H	
CPDPRG[2	waltz64	
PCPD2	80.00	usec
PLW2	8.80000019	W
PLW12	0.20176961	W
PLW13	0.10112690	W

F2 - Processing parameters

SI	65536	
SF	100.6555052	MHz
WDW	EM	
SSB	0	
LB	1.00	Hz
GB	0	
PC	1.40	

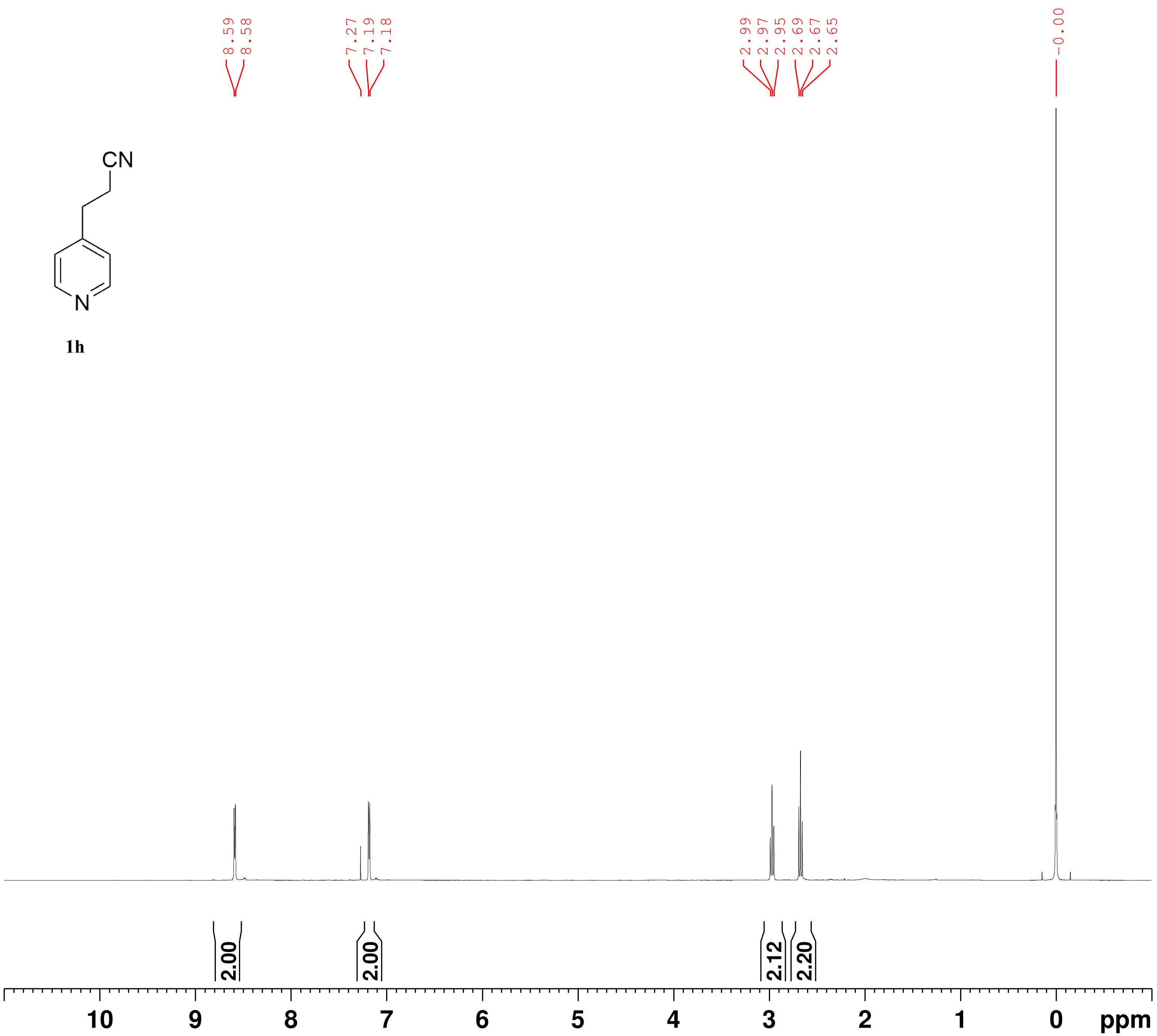

1h

Current Data Parameters
NAME 1H yl-07-073-re
EXPNO 1
PROCNO 1

F2 - Acquisition Parameters
Date_ 20220214
Time 14.55 h
INSTRUM Avance
PROBHD Z167430_0032 (
PULPROG zg30
TD 65536
SOLVENT CDCl3
NS 16
DS 2
SWH 8196.722 Hz
FIDRES 0.250144 Hz
AQ 3.9976959 sec
RG 101
DW 61.000 usec
DE 13.20 usec
TE 298.0 K
D1 1.00000000 sec
TD0 1
SFO1 400.3024719 MHz
NUC1 1H
P0 4.00 usec
P1 12.00 usec
PLW1 8.80000019 W

F2 - Processing parameters
SI 65536
SF 400.3000053 MHz
WDW EM
SSB 0
LB 0.30 Hz
GB 0
PC 1.00

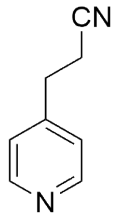

1h

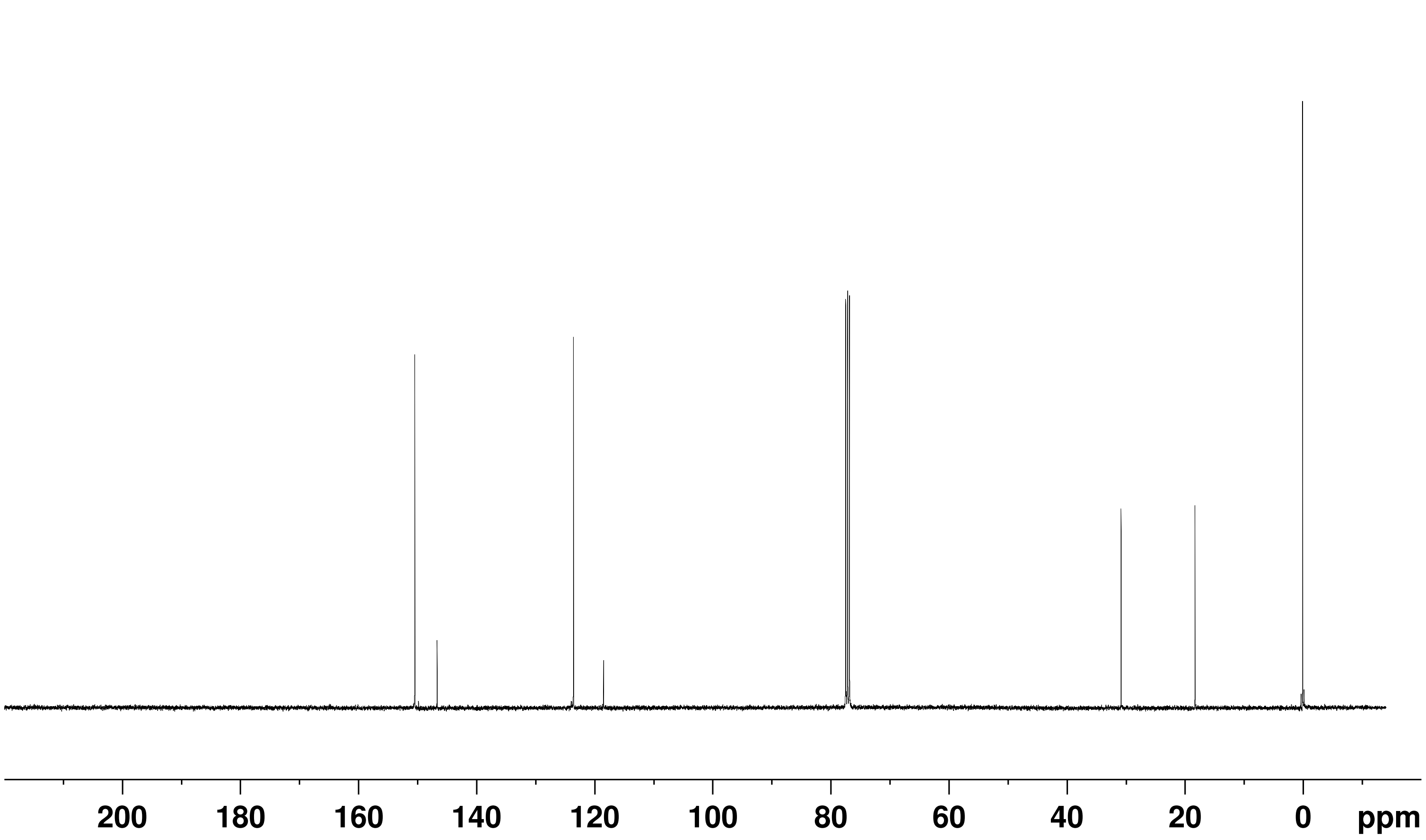

Current Data Parameters

NAME	13C yl-7-073
EXPNO	1
PROCNO	1

F2 - Acquisition Parameters

Date_	20220214
Time	15.06 h
INSTRUM	Avance
PROBHD	Z167430_0032 (
PULPROG	zgpg30
TD	65536
SOLVENT	CDCl3
NS	128
DS	4
SWH	25000.000 Hz
FIDRES	0.762939 Hz
AQ	1.3107200 sec
RG	3.25
DW	20.000 usec
DE	18.29 usec
TE	298.0 K
D1	2.00000000 sec
D11	0.03000000 sec
TD0	1
SFO1	100.6665872 MHz
NUC1	13C
P0	3.33 usec
P1	10.00 usec
PLW1	39.31399918 W
SFO2	400.3016012 MHz
NUC2	1H
CPDPRG[2	waltz64
PCPD2	80.00 usec
PLW2	8.80000019 W
PLW12	0.20176961 W
PLW13	0.10112690 W

F2 - Processing parameters

SI	131072
SF	100.6555046 MHz
WDW	EM
SSB	0
LB	1.00 Hz
GB	0
PC	1.40

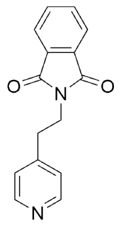

1i

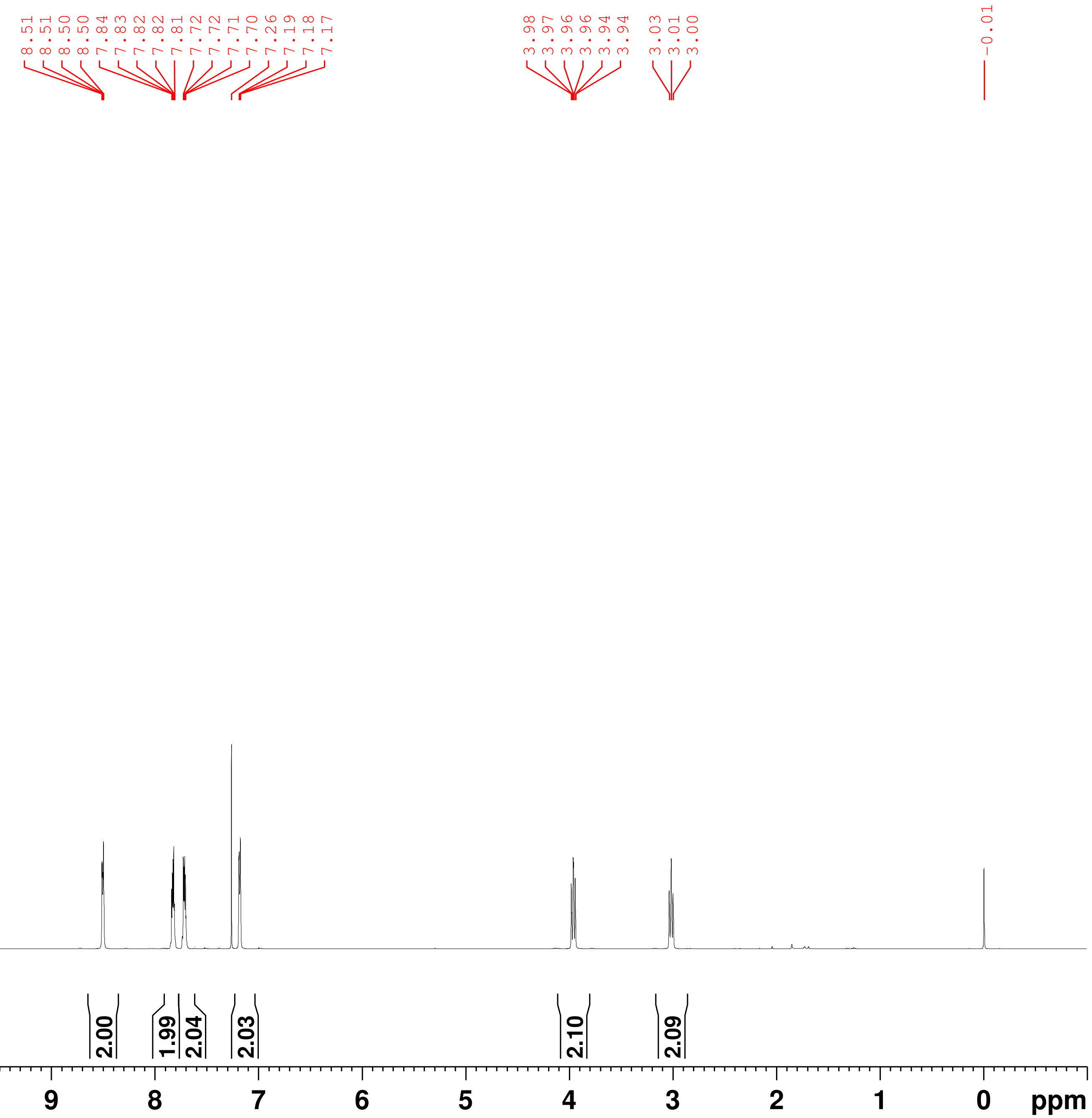


```
Current Data Parameters
NAME       1H_ST-7-233-re
EXPNO                   3
PROCNO                  1

F2 - Acquisition Parameters
Date_            20220531
Time                17.24 h
INSTRUM            Avance
PROBHD    Z167430_0032 (
PULPROG              zg30
TD                  65536
SOLVENT             CDCl3
NS                      4
DS                      0
SWH              8196.722 Hz
FIDRES           0.250144 Hz
AQ              3.9976959 sec
RG                    101
DW                 61.000 usec
DE                  13.20 usec
TE                  298.0 K
D1             0.10000000 sec
TD0                     1
SFO1          400.3024719 MHz
NUC1                   1H
P0                   4.00 usec
P1                  12.00 usec
PLW1           8.80000019 W

F2 - Processing parameters
SI                  65536
SF            400.3000098 MHz
WDW                    EM
SSB      0
LB                   0.30 Hz
GB       0
PC                   1.00
```

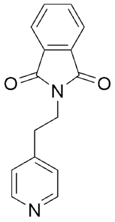

1i

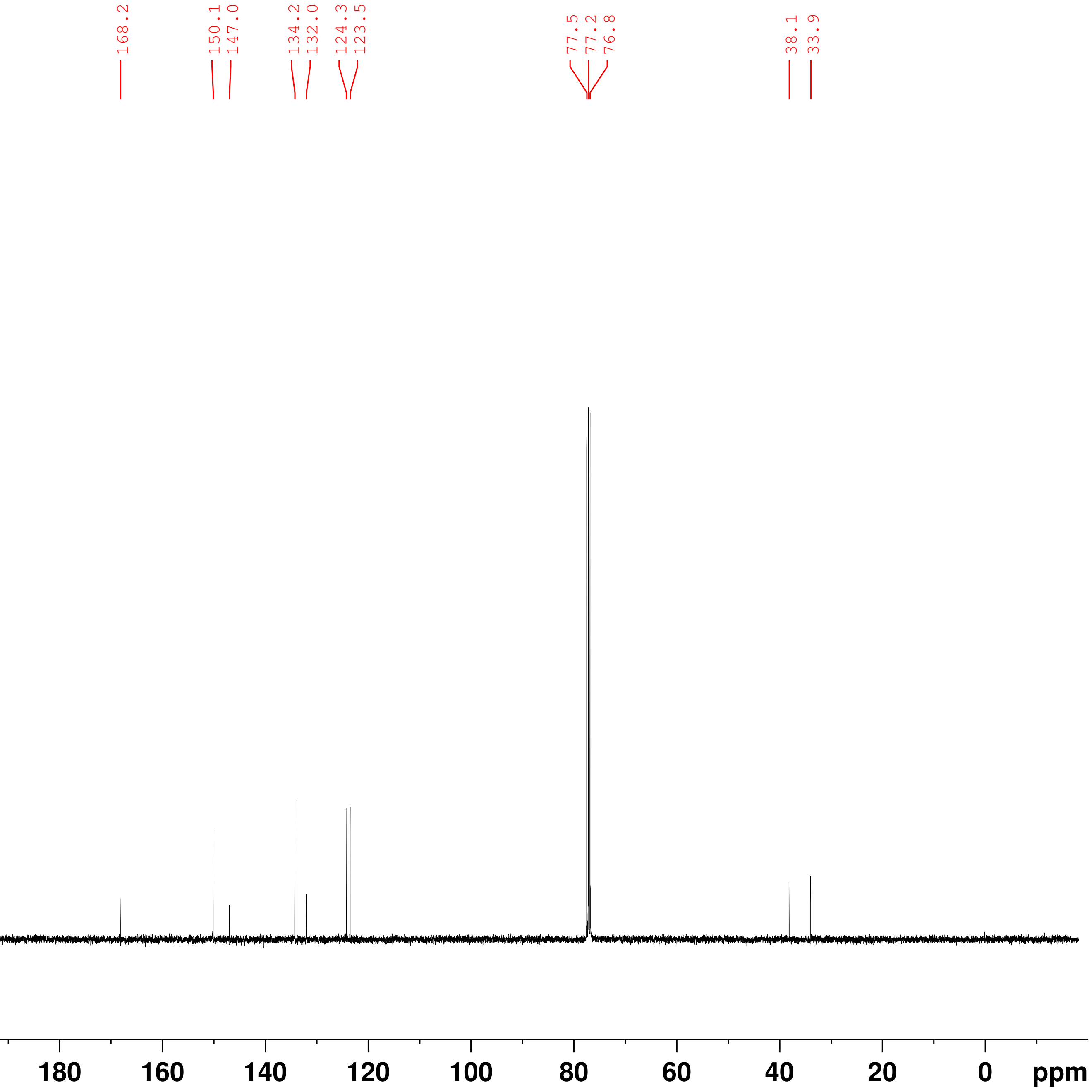

Current Data Parameters
NAME 13C_ST-7-233
EXPNO 3
PROCNO 1

F2 - Acquisition Parameters
Date_ 20220531
Time 17.23 h
INSTRUM Avance
PROBHD Z167430_0032 (
PULPROG zgpg30
TD 65536
SOLVENT CDCl3
NS 32
DS 4
SWH 23809.523 Hz
FIDRES 0.726609 Hz
AQ 1.3762560 sec
RG 3.25
DW 21.000 usec
DE 19.29 usec
TE 298.0 K
D1 3.00000000 sec
D11 0.03000000 sec
TD0 1
SFO1 100.6655806 MHz
NUC1 13C
P0 3.33 usec
P1 10.00 usec
PLW1 39.31399918 W
SFO2 400.3016012 MHz
NUC2 1H
CPDPRG[2 waltz64
PCPD2 80.00 usec
PLW2 8.80000019 W
PLW12 0.20176961 W
PLW13 0.10112690 W

F2 - Processing parameters
SI 131072
SF 100.6555029 MHz
WDW EM
SSB 0
LB 1.00 Hz
GB 0
PC 1.40

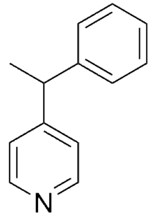

1m

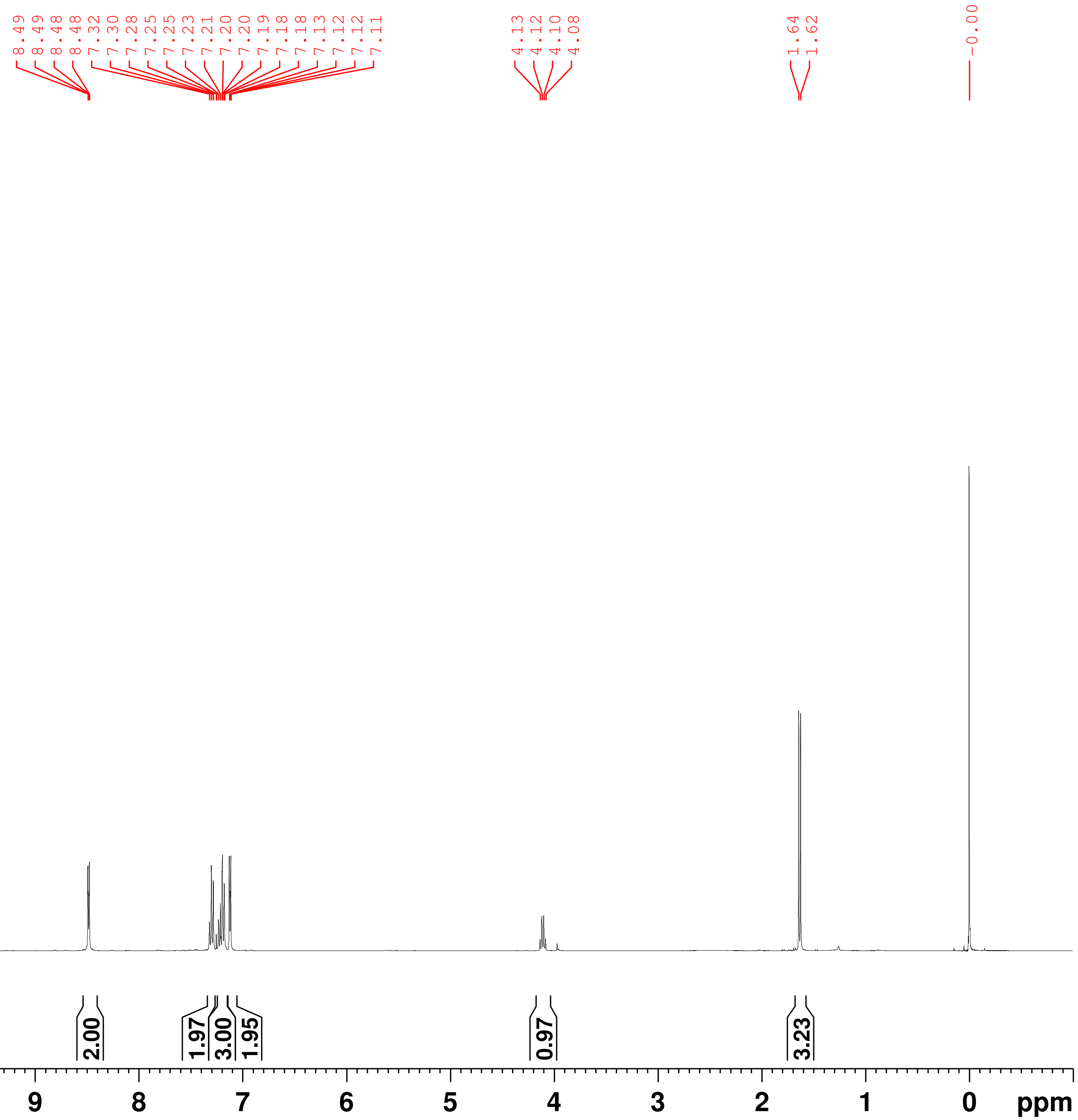

BRUKER

Current Data Parameters
NAME 1H gns-08-116
EXPNO 1
PROCNO 1

F2 - Acquisition Parameters
Date_ 20191213
Time 12.30
INSTRUM spect
PROBHD 5 mm Multinucl
PULPROG zg30
TD 32768
SOLVENT CDCl3
NS 16
DS 0
SWH 8012.820 Hz
FIDRES 0.244532 Hz
AQ 2.0447233 sec
RG 114
DW 62.400 usec
DE 6.50 usec
TE 298.2 K
D1 0.01000000 sec
TD0 1

======== CHANNEL f1 ========
NUC1 1H
P1 7.20 usec
PL1 -5.00 dB
SFO1 400.1332010 MHz

F2 - Processing parameters
SI 131072
SF 400.1300122 MHz
WDW EM
SSB 0
LB 0.25 Hz
GB 0
PC 0.20

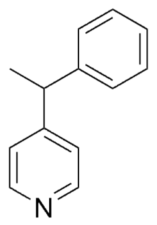

1m

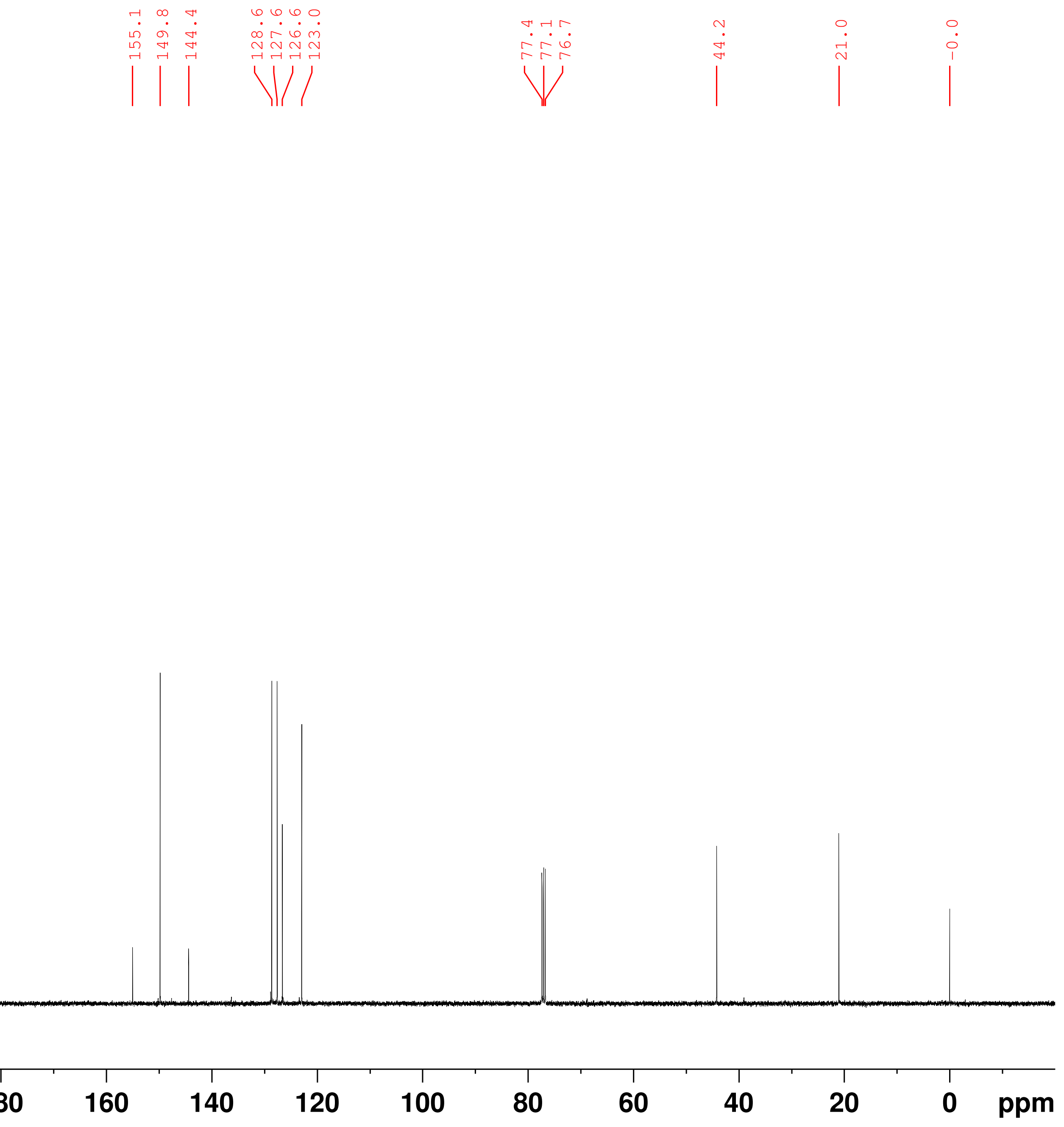

BRUKER

Current Data Parameters
NAME 13C gns-08-116
EXPNO 1
PROCNO 1

F2 - Acquisition Parameters
Date_ 20191213
Time 12.41
INSTRUM spect
PROBHD 5 mm Multinucl
PULPROG zgdc30
TD 65536
SOLVENT CDCl3
NS 300
DS 4
SWH 26246.719 Hz
FIDRES 0.400493 Hz
AQ 1.2484608 sec
RG 161.3
DW 19.050 usec
DE 6.50 usec
TE 298.2 K
D1 0.69999999 sec
d11 0.03000000 sec
TD0 1

======== CHANNEL f1 ========
NUC1 13C
P1 8.07 usec
PL1 -6.00 dB
SFO1 100.6196894 MHz

======== CHANNEL f2 ========
CPDPRG[2 waltz16
NUC2 1H
PCPD2 80.00 usec
PL2 0 dB
PL12 18.00 dB
SFO2 400.1318006 MHz

F2 - Processing parameters
SI 131072
SF 100.6127701 MHz
WDW EM
SSB 0
LB 0.80 Hz
GB 0
PC 0.50

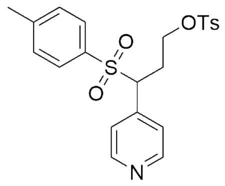

3aa

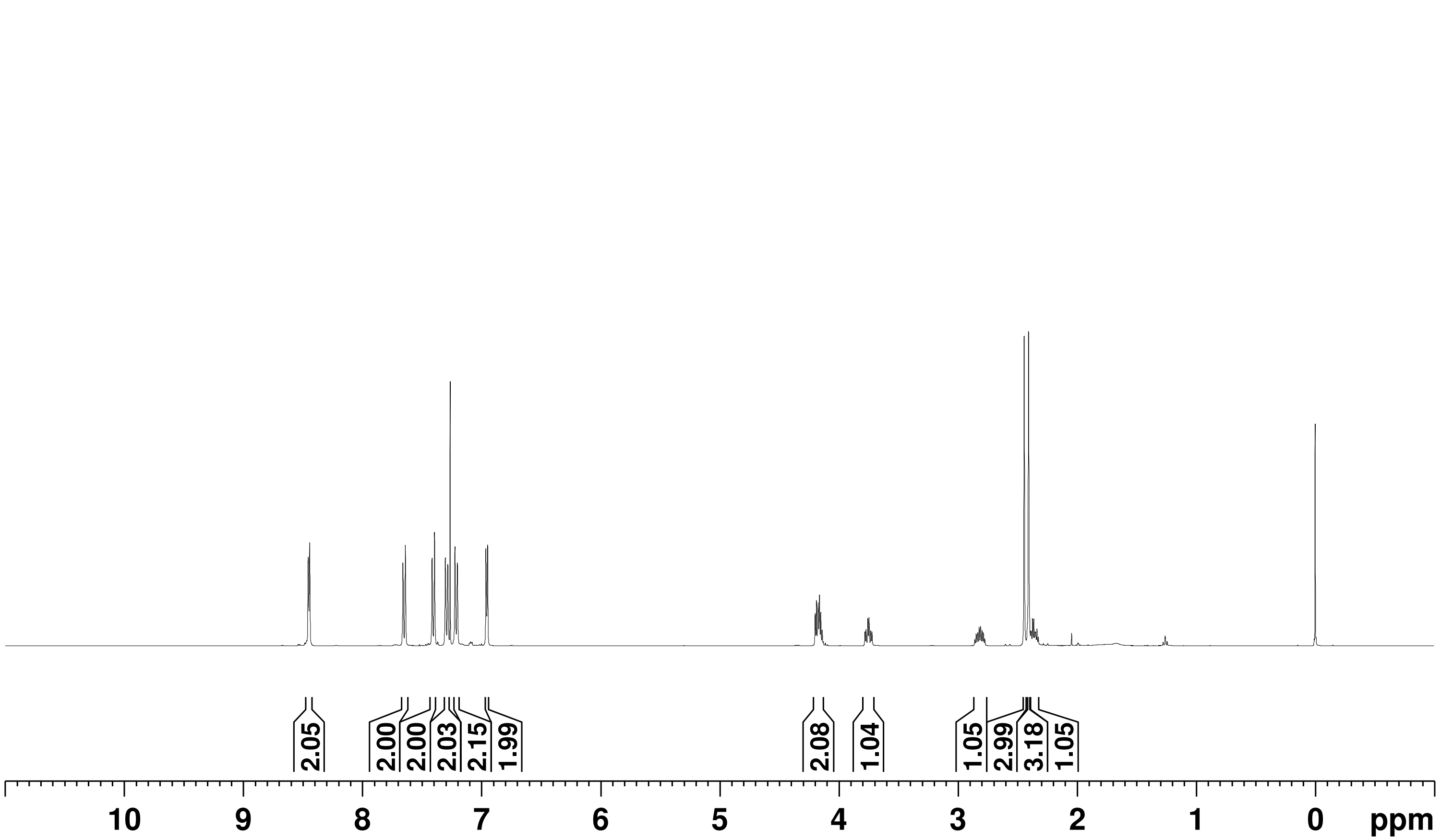


```
Current Data Parameters
NAME        1H_ST-7-89-re1
EXPNO                    2
PROCNO                   1

F2 - Acquisition Parameters
Date_             20220906
Time                 11.21 h
INSTRUM             Avance
PROBHD     Z167430_0032 (
PULPROG               zg30
TD                   65536
SOLVENT              CDCl3
NS                      16
DS                       0
SWH               8196.722 Hz
FIDRES            0.250144 Hz
AQ               3.9976959 sec
RG                     101
DW                  61.000 usec
DE                   13.20 usec
TE                   298.0 K
D1              0.10000000 sec
TD0                      1
SFO1           400.3024719 MHz
NUC1                    1H
P0                    4.00 usec
P1                   12.00 usec
PLW1            8.80000019 W

F2 - Processing parameters
SI                   65536
SF             400.3000086 MHz
WDW                     EM
SSB       0
LB                    0.30 Hz
GB        0
PC                    1.00
```

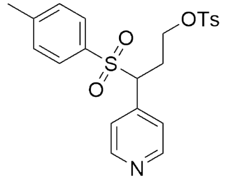

3aa

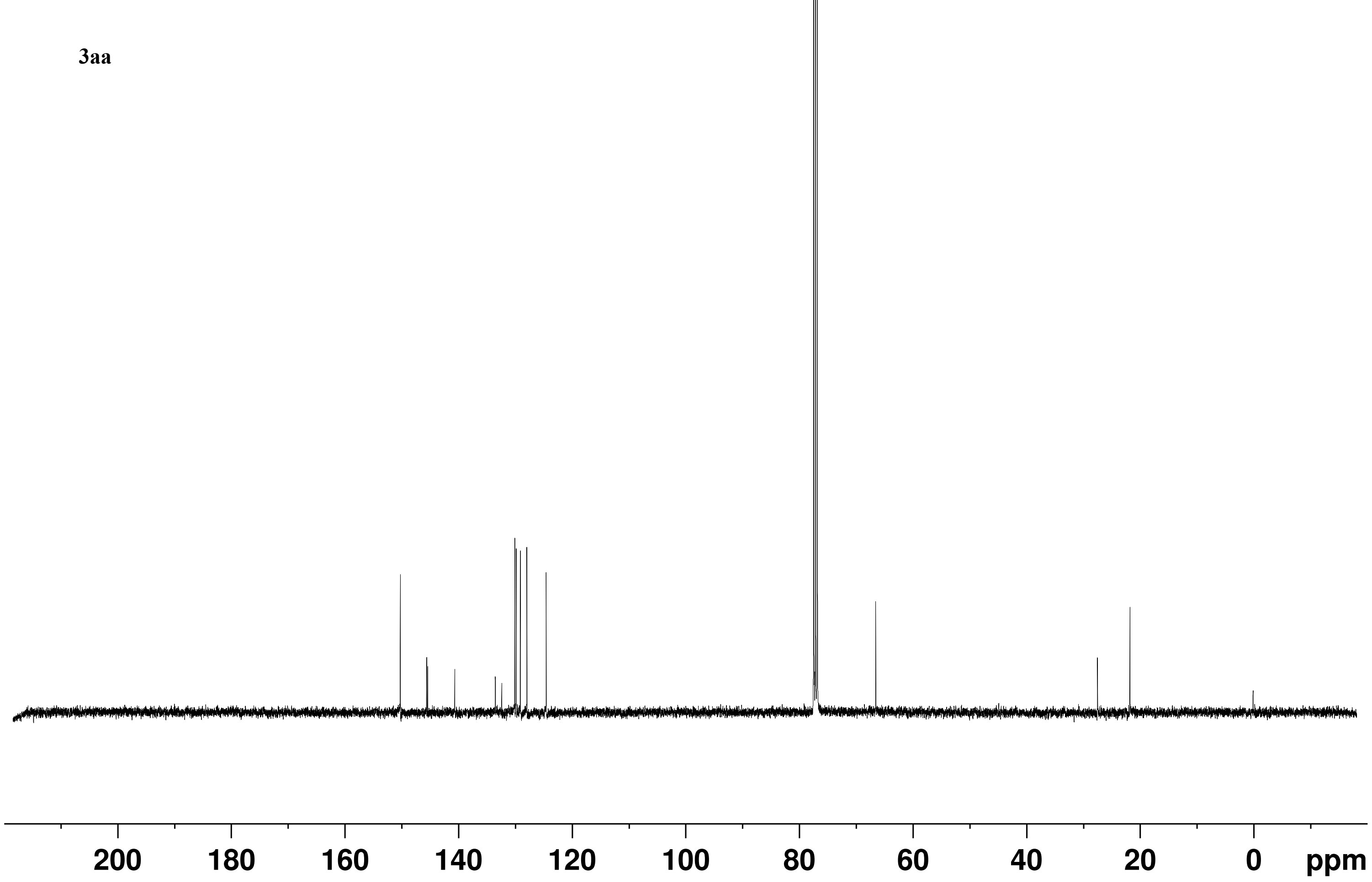

BRUKER

Current Data Parameters
NAME 13C_ST-7-89-re1
EXPNO 1
PROCNO 1

F2 - Acquisition Parameters

Date_	20220906	
Time	11.29	h
INSTRUM	Avance	
PROBHD	Z167430_0032 (	
PULPROG	zgpg30	
TD	65536	
SOLVENT	CDCl3	
NS	128	
DS	4	
SWH	23809.523	Hz
FIDRES	0.726609	Hz
AQ	1.3762560	sec
RG	3.25	
DW	21.000	usec
DE	19.29	usec
TE	298.0	K
D1	2.00000000	sec
D11	0.03000000	sec
TD0	1	
SFO1	100.6655806	MHz
NUC1	13C	
P0	3.33	usec
P1	10.00	usec
PLW1	39.31399918	W
SFO2	400.3016012	MHz
NUC2	1H	
CPDPRG[2	waltz64	
PCPD2	80.00	usec
PLW2	8.80000019	W
PLW12	0.20176961	W
PLW13	0.10112690	W

F2 - Processing parameters

SI	131072	
SF	100.6555025	MHz
WDW	EM	
SSB	0	
LB	1.00	Hz
GB	0	
PC	1.40	

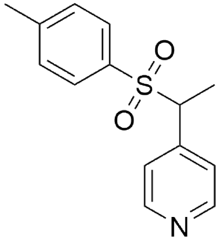

3ba

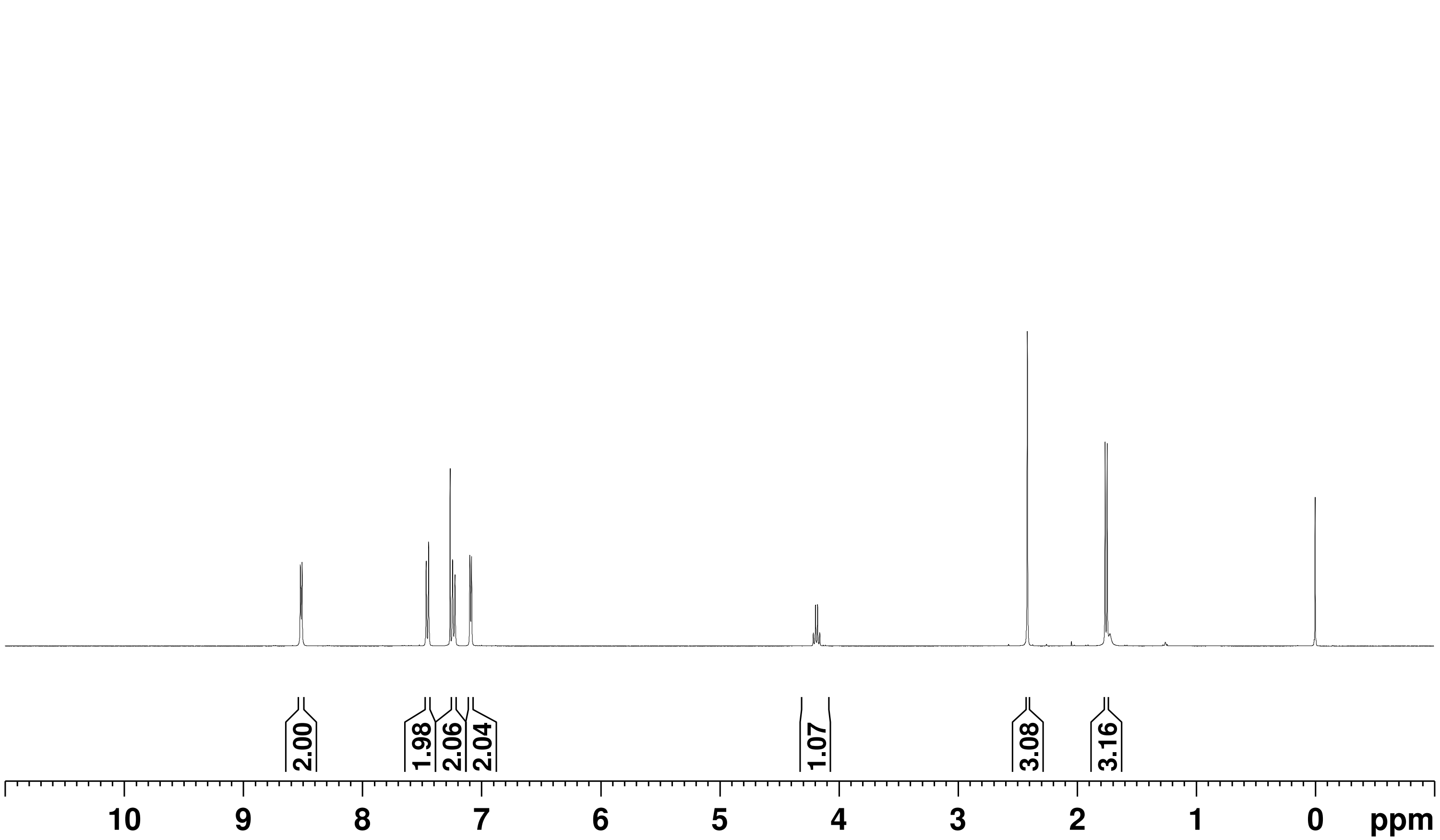

BRUKER

```
Current Data Parameters
NAME          1H ST-07-179
EXPNO                    3
PROCNO                   1

F2 - Acquisition Parameters
Date_             20220508
Time                 14.47 h
INSTRUM             Avance
PROBHD     Z167430_0032 (
PULPROG               zg30
TD                   65536
SOLVENT              CDCl3
NS                      16
DS                       0
SWH               8196.722 Hz
FIDRES            0.250144 Hz
AQ               3.9976959 sec
RG                     101
DW                  61.000 usec
DE                   13.20 usec
TE                   298.0 K
D1              0.10000000 sec
TD0                      1
SFO1           400.3024719 MHz
NUC1                    1H
P0                    4.00 usec
P1                   12.00 usec
PLW1            8.80000019 W

F2 - Processing parameters
SI                   65536
SF             400.3000087 MHz
WDW                     EM
SSB      0
LB                    0.30 Hz
GB       0
PC                    1.00
```

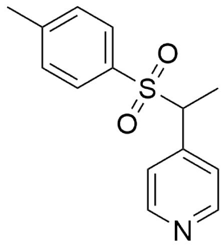

3ba

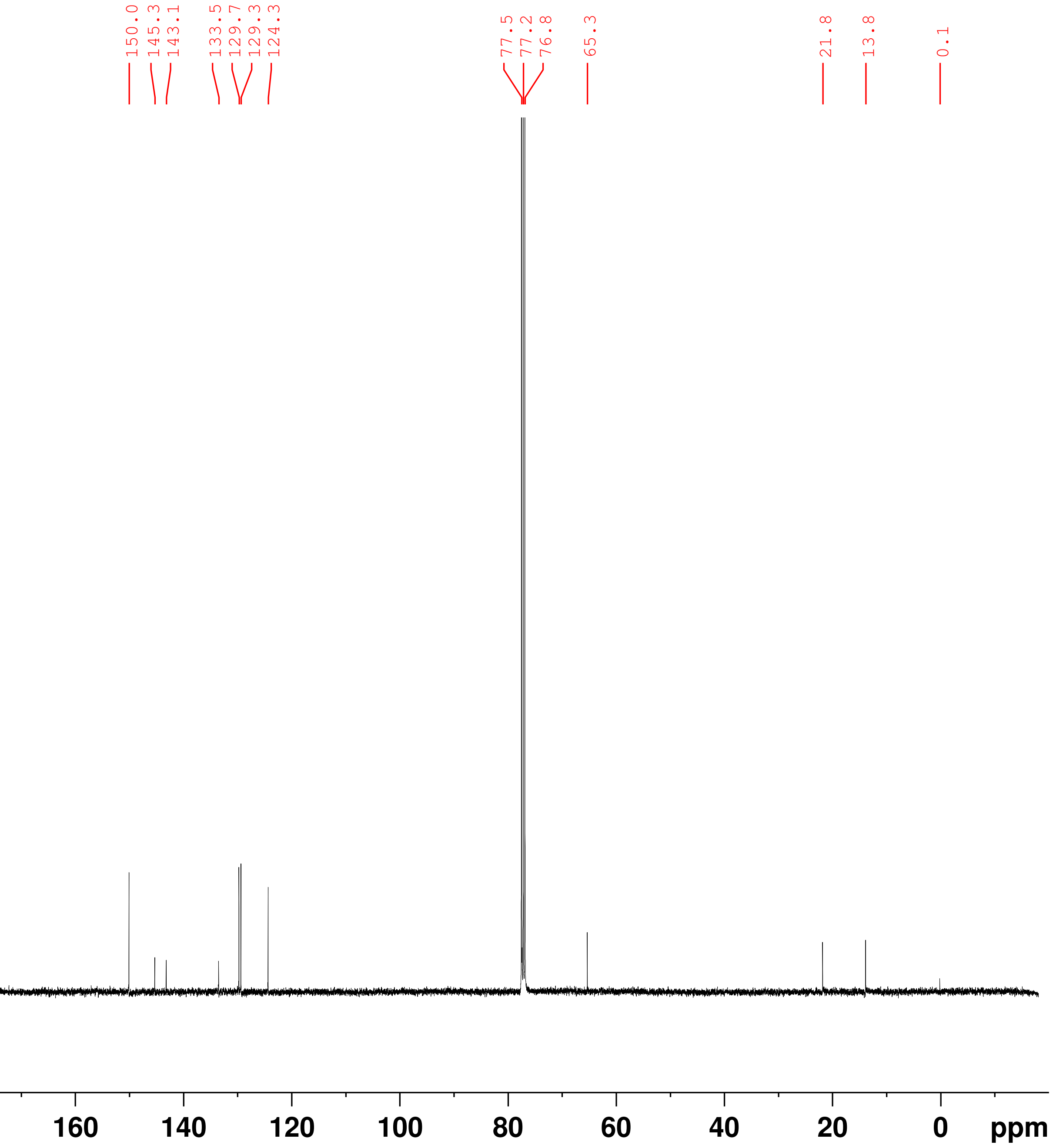

Current Data Parameters
NAME 13C_ST-7-179
EXPNO 5
PROCNO 1

F2 - Acquisition Parameters
Date_ 20220508
Time 14.45 h
INSTRUM Avance
PROBHD Z167430_0032 (
PULPROG zgpg30
TD 65536
SOLVENT CDCl3
NS 128
DS 4
SWH 23809.523 Hz
FIDRES 0.726609 Hz
AQ 1.3762560 sec
RG 3.25
DW 21.000 usec
DE 19.29 usec
TE 298.0 K
D1 3.00000000 sec
D11 0.03000000 sec
TD0 1
SFO1 100.6655806 MHz
NUC1 13C
P0 3.33 usec
P1 10.00 usec
PLW1 39.31399918 W
SFO2 400.3016012 MHz
NUC2 1H
CPDPRG[2 waltz64
PCPD2 80.00 usec
PLW2 8.80000019 W
PLW12 0.20176961 W
PLW13 0.10112690 W

F2 - Processing parameters
SI 131072
SF 100.6555021 MHz
WDW EM
SSB 0
LB 1.00 Hz
GB 0
PC 1.40

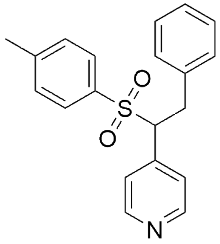

3ca

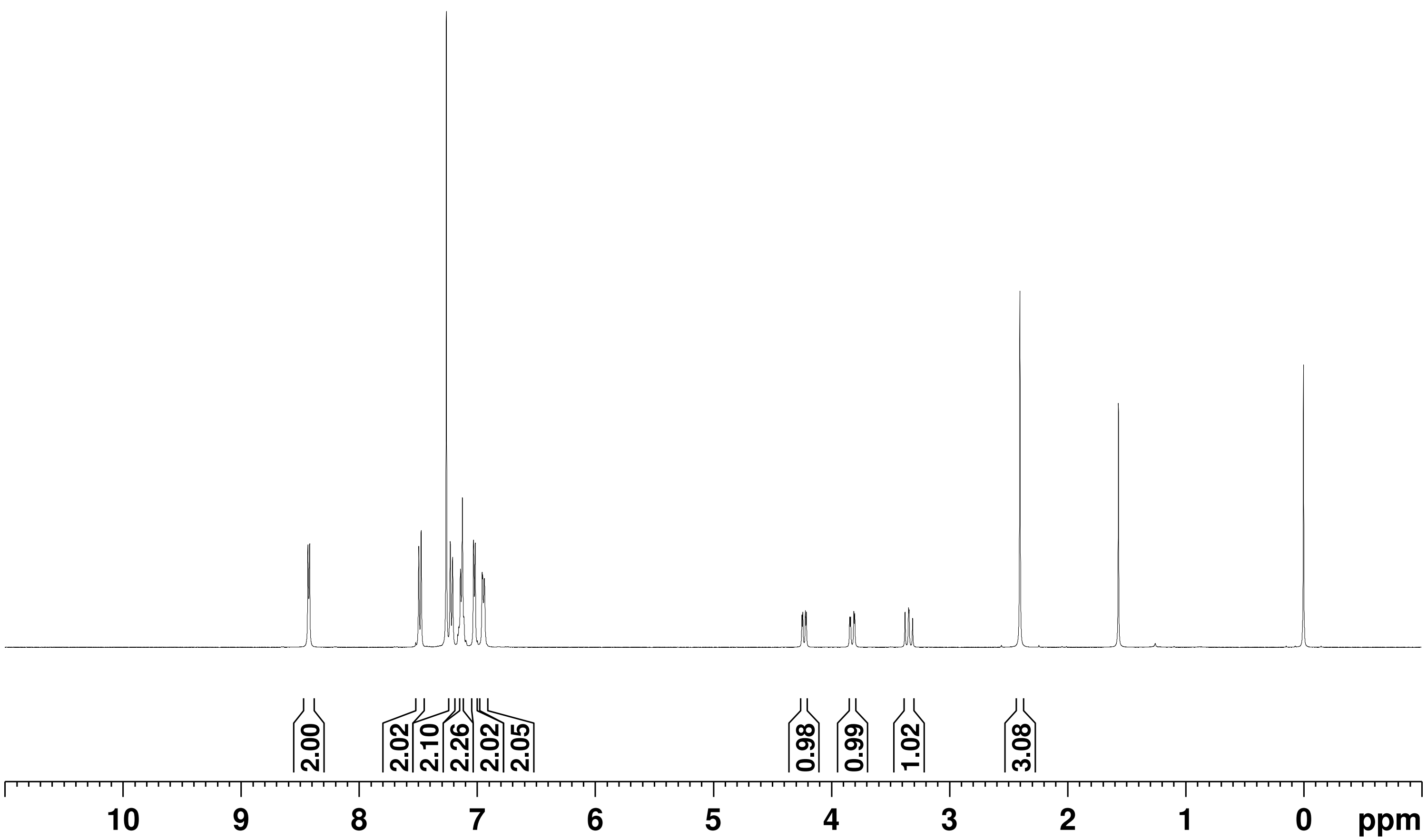

Current Data Parameters

NAME	1H_ST-7-227-again
EXPNO	1
PROCNO	1

F2 - Acquisition Parameters

Date_	20220601
Time	9.29 h
INSTRUM	Avance
PROBHD	Z167430_0032 (
PULPROG	zg30
TD	65536
SOLVENT	CDCl3
NS	16
DS	0
SWH	8196.722 Hz
FIDRES	0.250144 Hz
AQ	3.9976959 sec
RG	101
DW	61.000 usec
DE	13.20 usec
TE	298.0 K
D1	0.10000000 sec
TD0	1
SFO1	400.3024719 MHz
NUC1	1H
P0	4.00 usec
P1	12.00 usec
PLW1	8.80000019 W

F2 - Processing parameters

SI		65536
SF		400.3000097 MHz
WDW		EM
SSB	0	
LB		0.30 Hz
GB	0	
PC		1.00

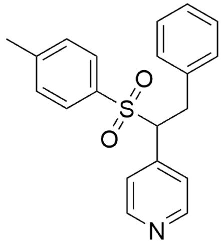

3ca

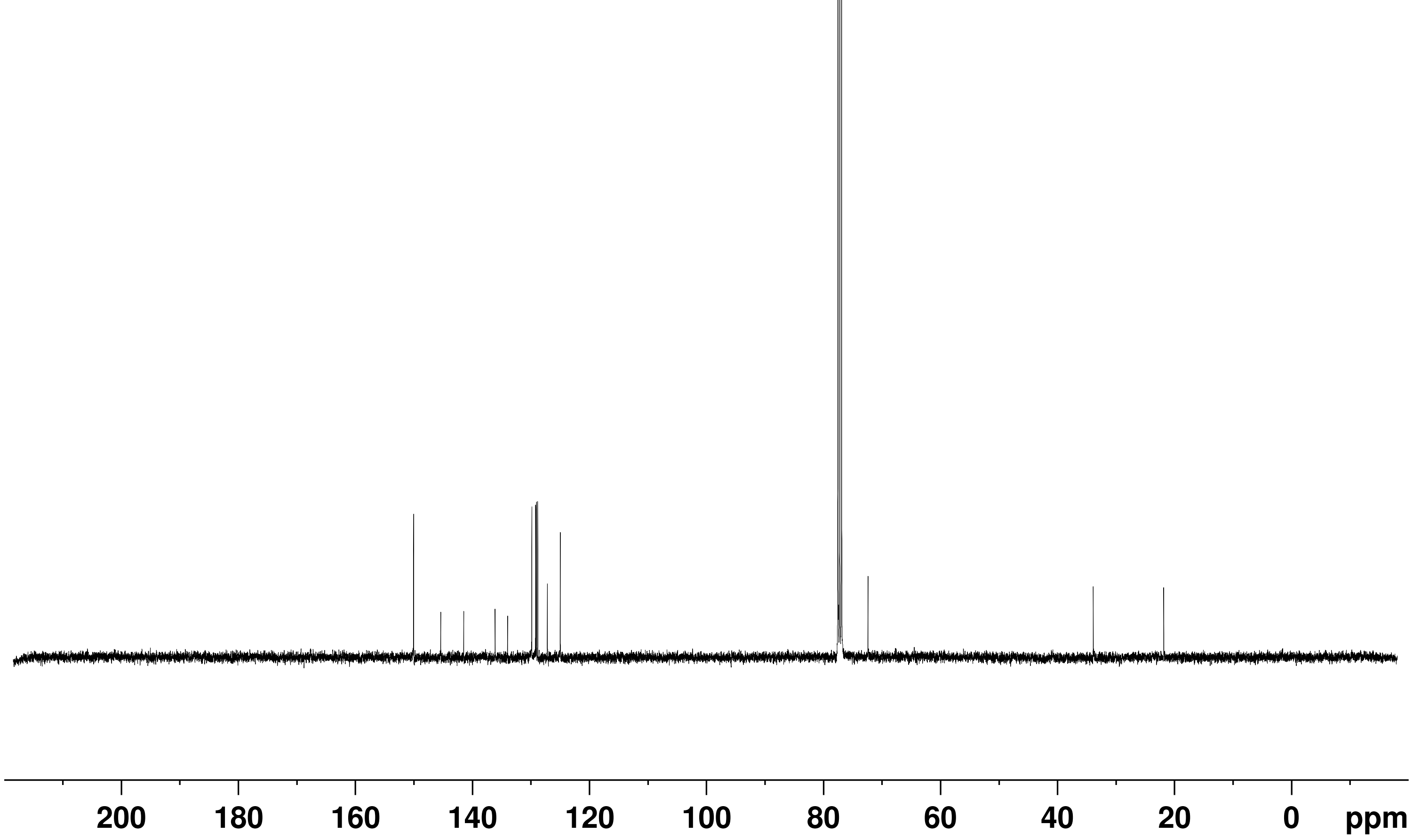

Current Data Parameters
NAME 13C_ST-7-227-real
EXPNO 1
PROCNO 1

F2 - Acquisition Parameters
Date_ 20220601
Time 18.19 h
INSTRUM Avance
PROBHD Z167430_0032 (
PULPROG zgpg30
TD 65536
SOLVENT CDCl3
NS 64
DS 4
SWH 23809.523 Hz
FIDRES 0.726609 Hz
AQ 1.3762560 sec
RG 3.25
DW 21.000 usec
DE 19.29 usec
TE 298.0 K
D1 3.00000000 sec
D11 0.03000000 sec
TD0 1
SFO1 100.6655806 MHz
NUC1 13C
P0 3.33 usec
P1 10.00 usec
PLW1 39.31399918 W
SFO2 400.3016012 MHz
NUC2 1H
CPDPRG[2 waltz64
PCPD2 80.00 usec
PLW2 8.80000019 W
PLW12 0.20176961 W
PLW13 0.10112690 W

F2 - Processing parameters
SI 131072
SF 100.6555028 MHz
WDW EM
SSB 0
LB 1.00 Hz
GB 0
PC 1.40

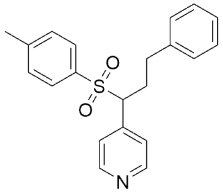

3da

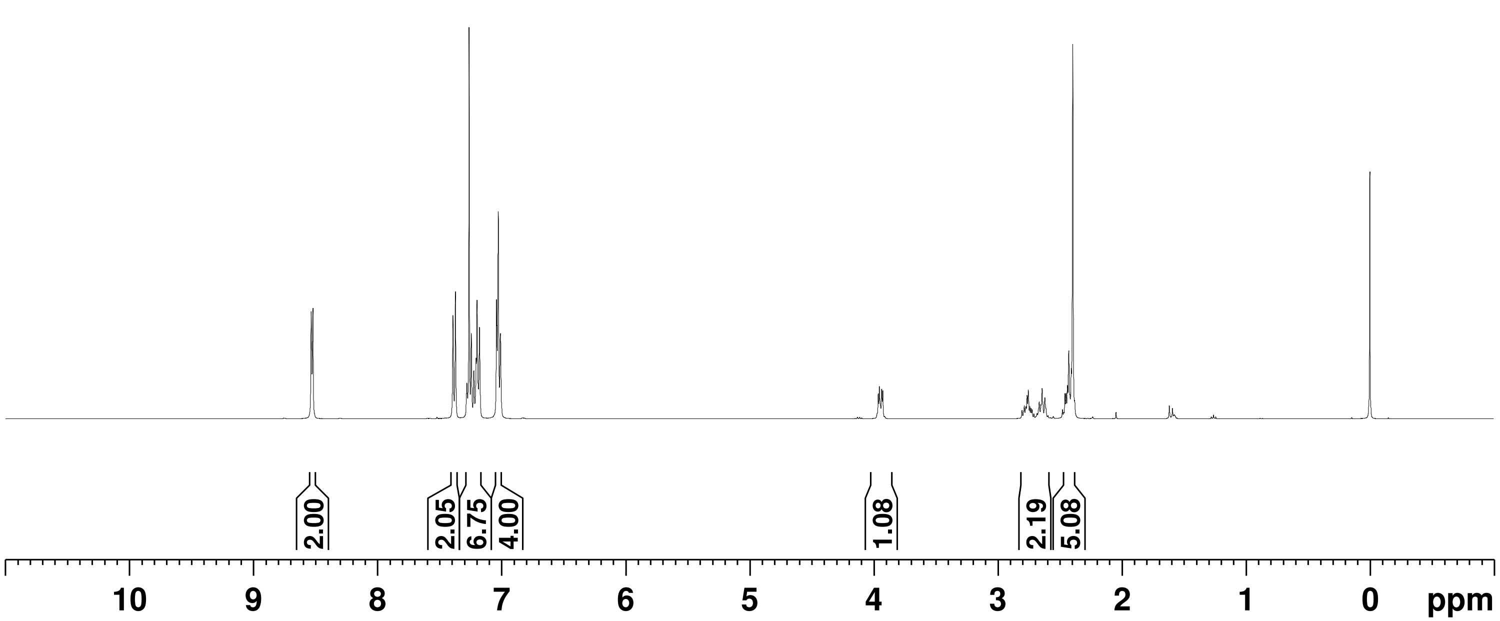

Current Data Parameters
NAME 1H_ST-07-259
EXPNO 1
PROCNO 1

F2 - Acquisition Parameters
Date_ 20220620
Time 17.44 h
INSTRUM Avance
PROBHD Z167430_0032 (
PULPROG zg30
TD 65536
SOLVENT CDCl3
NS 16
DS 0
SWH 8196.722 Hz
FIDRES 0.250144 Hz
AQ 3.9976959 sec
RG 101
DW 61.000 usec
DE 13.20 usec
TE 298.0 K
D1 0.10000000 sec
TD0 1
SFO1 400.3024719 MHz
NUC1 1H
P0 4.00 usec
P1 12.00 usec
PLW1 8.80000019 W

F2 - Processing parameters
SI 65536
SF 400.3000094 MHz
WDW EM
SSB 0
LB 0.30 Hz
GB 0
PC 1.00

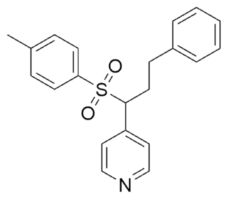

3da

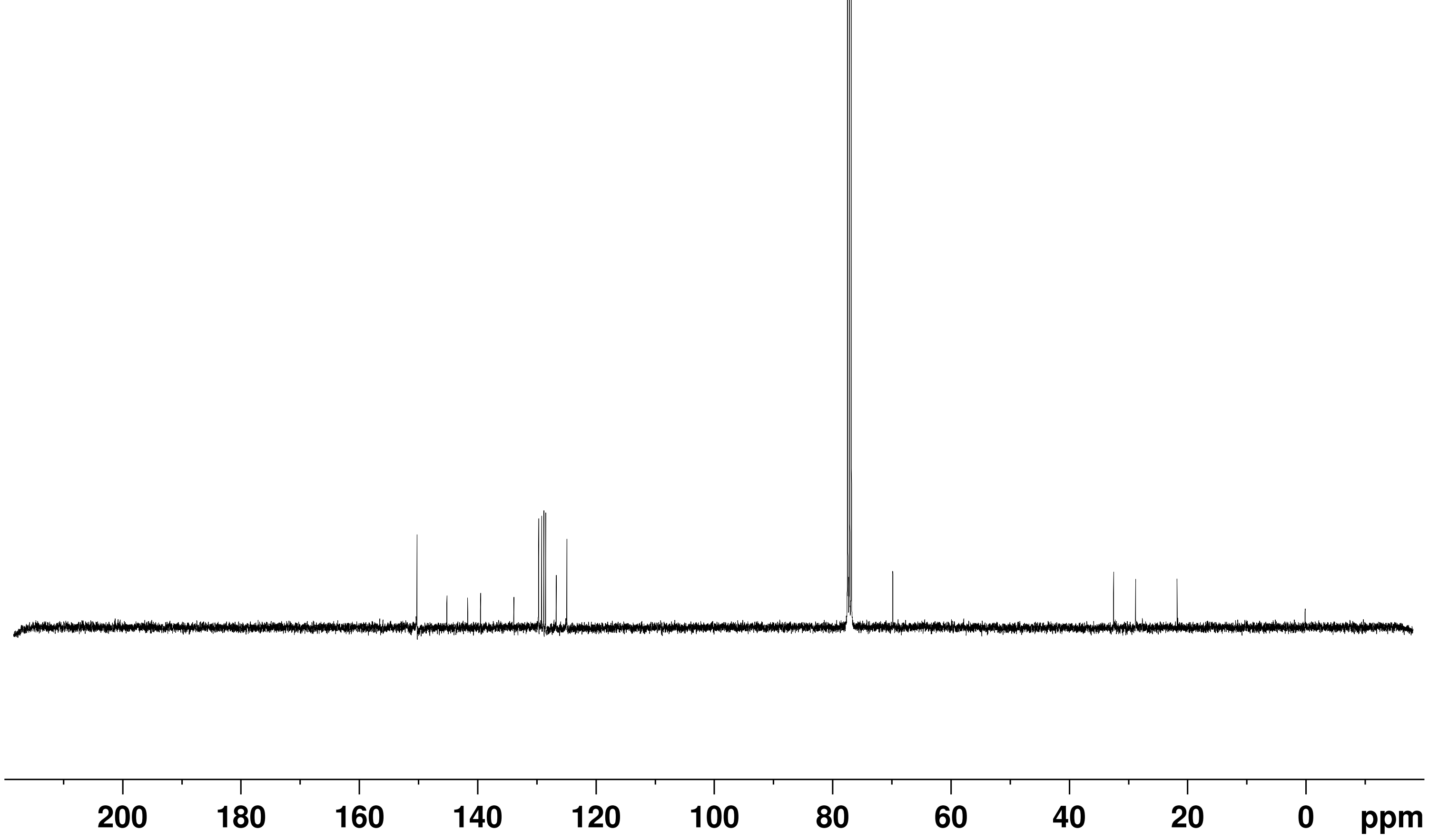

BRUKER

Current Data Parameters
NAME 13C_ST-7-259
EXPNO 1
PROCNO 1

F2 - Acquisition Parameters
Date_ 20220620
Time 17.54 h
INSTRUM Avance
PROBHD Z167430_0032 (
PULPROG zgpg30
TD 65536
SOLVENT CDCl3
NS 128
DS 4
SWH 23809.523 Hz
FIDRES 0.726609 Hz
AQ 1.3762560 sec
RG 3.25
DW 21.000 usec
DE 19.29 usec
TE 298.0 K
D1 3.00000000 sec
D11 0.03000000 sec
TD0 1
SFO1 100.6655806 MHz
NUC1 13C
P0 3.33 usec
P1 10.00 usec
PLW1 39.31399918 W
SFO2 400.3016012 MHz
NUC2 1H
CPDPRG[2 waltz64
PCPD2 80.00 usec
PLW2 8.80000019 W
PLW12 0.20176961 W
PLW13 0.10112690 W

F2 - Processing parameters
SI 131072
SF 100.6555022 MHz
WDW EM
SSB 0
LB 1.00 Hz
GB 0
PC 1.40

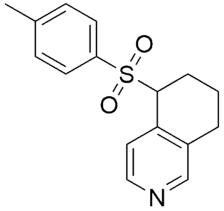

3ea

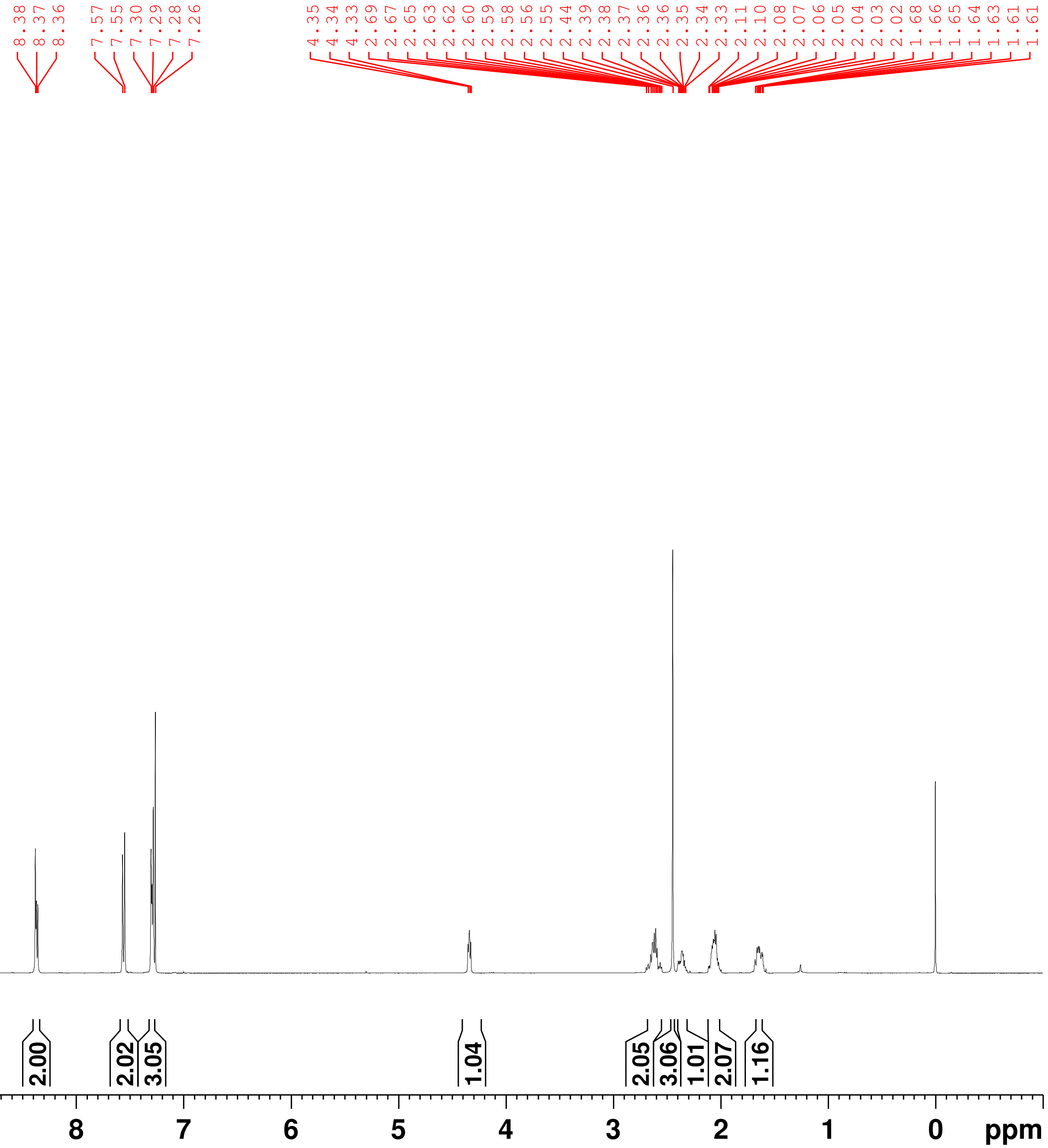

Current Data Parameters

NAME	1H ST-07-189
EXPNO	4
PROCNO	1

F2 - Acquisition Parameters

Date_	20220508
Time	14.11 h
INSTRUM	Avance
PROBHD	Z167430_0032 (
PULPROG	zg30
TD	65536
SOLVENT	CDCl3
NS	16
DS	0
SWH	8196.722 Hz
FIDRES	0.250144 Hz
AQ	3.9976959 sec
RG	101
DW	61.000 usec
DE	13.20 usec
TE	298.0 K
D1	0.10000000 sec
TD0	1
SFO1	400.3024719 MHz
NUC1	1H
P0	4.00 usec
P1	12.00 usec
PLW1	8.80000019 W

F2 - Processing parameters

SI	65536
SF	400.3000087 MHz
WDW	EM
SSB	0
LB	0.30 Hz
GB	0
PC	1.00

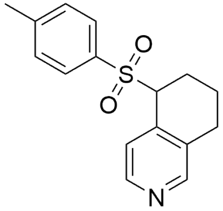

3ea

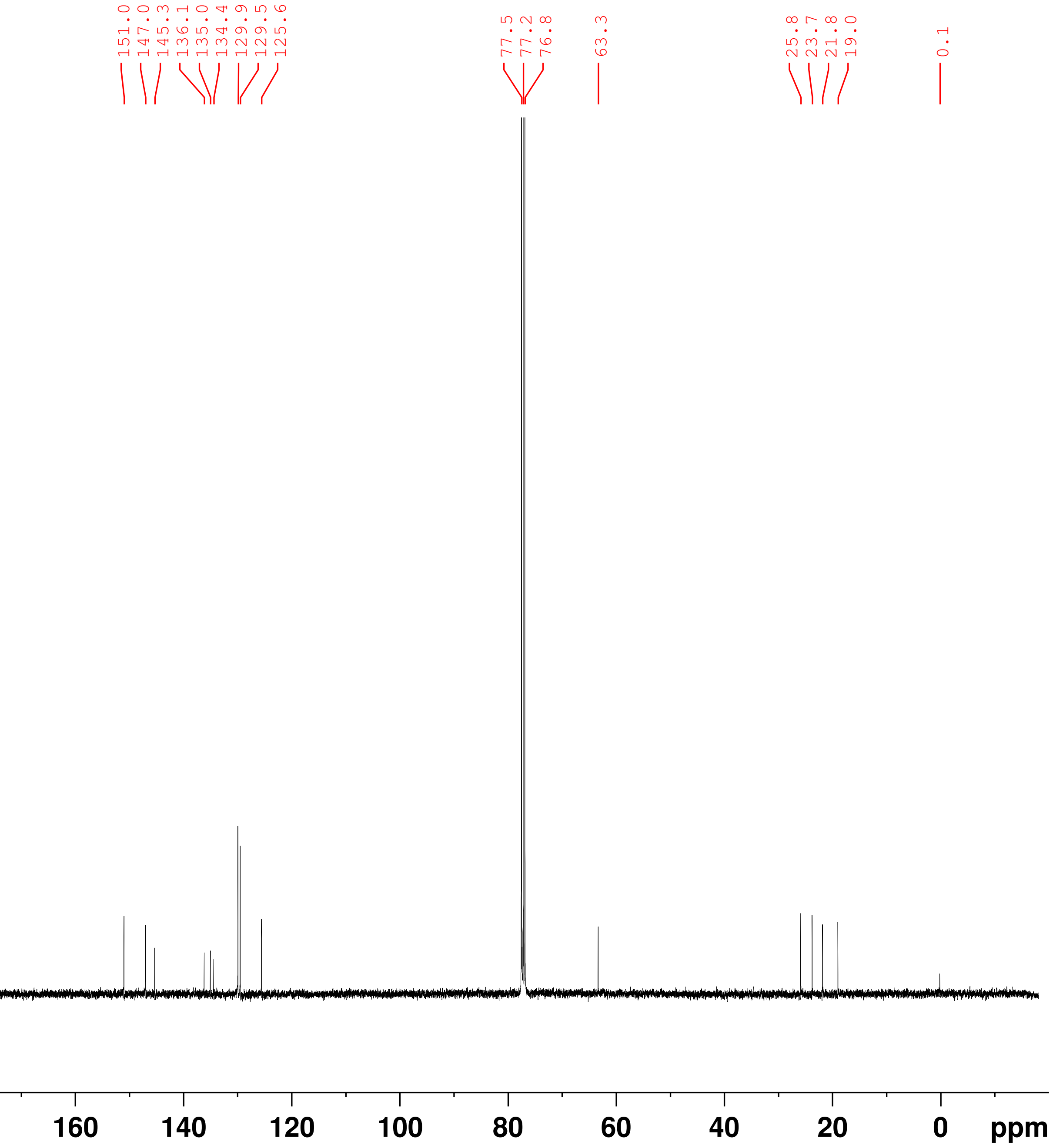


```
Current Data Parameters
NAME          13C_ST-7-189
EXPNO                    4
PROCNO                   1

F2 - Acquisition Parameters
Date_             20220508
Time                 14.27 h
INSTRUM             Avance
PROBHD     Z167430_0032 (
PULPROG            zgpg30
TD                   65536
SOLVENT              CDCl3
NS                     128
DS                       4
SWH              23809.523 Hz
FIDRES            0.726609 Hz
AQ               1.3762560 sec
RG                    3.25
DW                  21.000 usec
DE                   19.29 usec
TE                   298.0 K
D1              3.00000000 sec
D11             0.03000000 sec
TD0                      1
SFO1           100.6655806 MHz
NUC1                   13C
P0                    3.33 usec
P1                   10.00 usec
PLW1           39.31399918 W
SFO2           400.3016012 MHz
NUC2                    1H
CPDPRG[2           waltz64
PCPD2                80.00 usec
PLW2            8.80000019 W
PLW12           0.20176961 W
PLW13           0.10112690 W

F2 - Processing parameters
SI                  131072
SF             100.6555023 MHz
WDW                     EM
SSB         0
LB                    1.00 Hz
GB          0
PC                    1.40
```

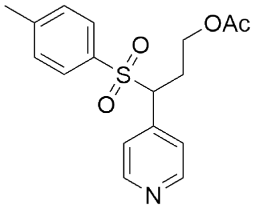

3fa

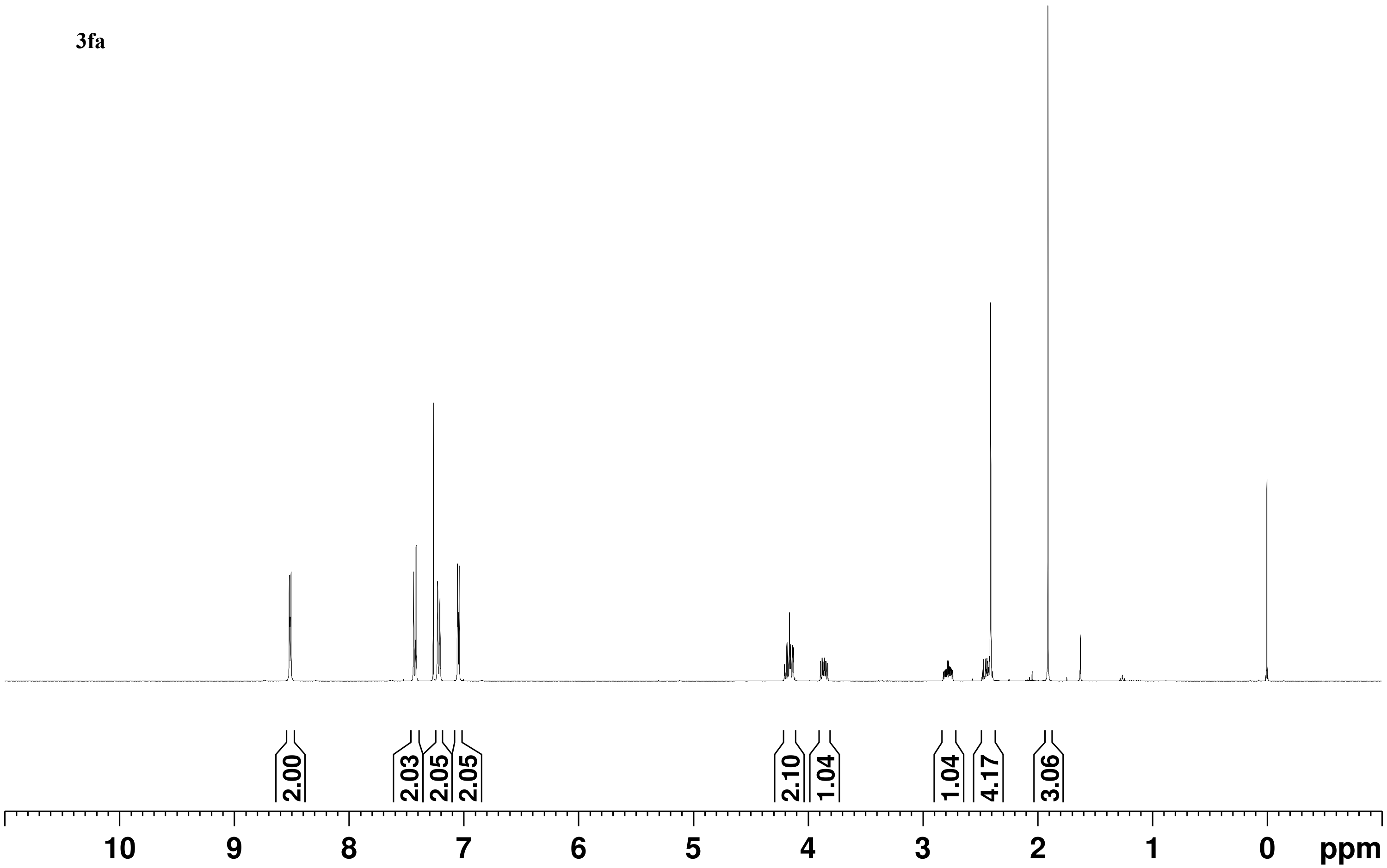


```
Current Data Parameters
NAME          1H_ST-7-229
EXPNO                   1
PROCNO                  1

F2 - Acquisition Parameters
Date_            20220525
Time                 9.15 h
INSTRUM            Avance
PROBHD    Z167430_0032 (
PULPROG              zg30
TD                  65536
SOLVENT             CDCl3
NS                     16
DS                      0
SWH              8196.722 Hz
FIDRES           0.250144 Hz
AQ              3.9976959 sec
RG                    101
DW                 61.000 usec
DE                  13.20 usec
TE                  298.0 K
D1             0.10000000 sec
TD0                     1
SFO1          400.3024719 MHz
NUC1                   1H
P0                   4.00 usec
P1                  12.00 usec
PLW1           8.80000019 W

F2 - Processing parameters
SI                  65536
SF            400.3000080 MHz
WDW                    no
SSB       0
LB        0 Hz
GB        0
PC                   1.00
```

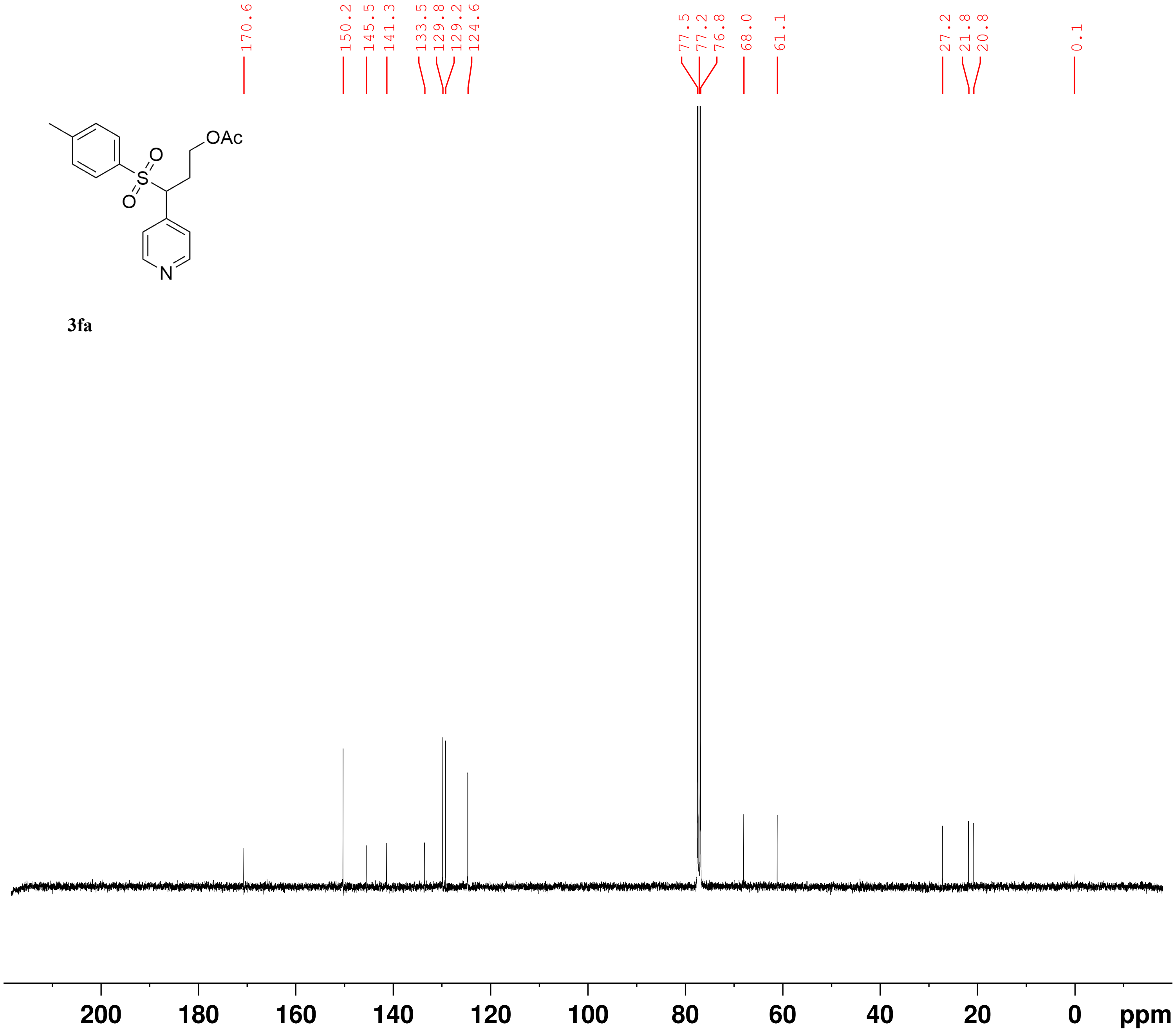

Current Data Parameters
NAME 13C_ST-7-229
EXPNO 2
PROCNO 1

F2 - Acquisition Parameters
Date_ 20220525
Time 9.29 h
INSTRUM Avance
PROBHD Z167430_0032 (
PULPROG zgpg30
TD 65536
SOLVENT CDCl3
NS 128
DS 4
SWH 23809.523 Hz
FIDRES 0.726609 Hz
AQ 1.3762560 sec
RG 3.25
DW 21.000 usec
DE 19.29 usec
TE 298.0 K
D1 3.00000000 sec
D11 0.03000000 sec
TD0 1
SFO1 100.6655806 MHz
NUC1 13C
P0 3.33 usec
P1 10.00 usec
PLW1 39.31399918 W
SFO2 400.3016012 MHz
NUC2 1H
CPDPRG[2 waltz64
PCPD2 80.00 usec
PLW2 8.80000019 W
PLW12 0.20176961 W
PLW13 0.10112690 W

F2 - Processing parameters
SI 131072
SF 100.6555023 MHz
WDW EM
SSB 0
LB 1.00 Hz
GB 0
PC 1.40

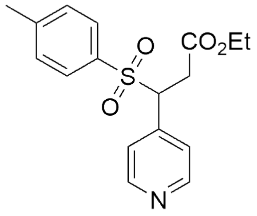

3ga

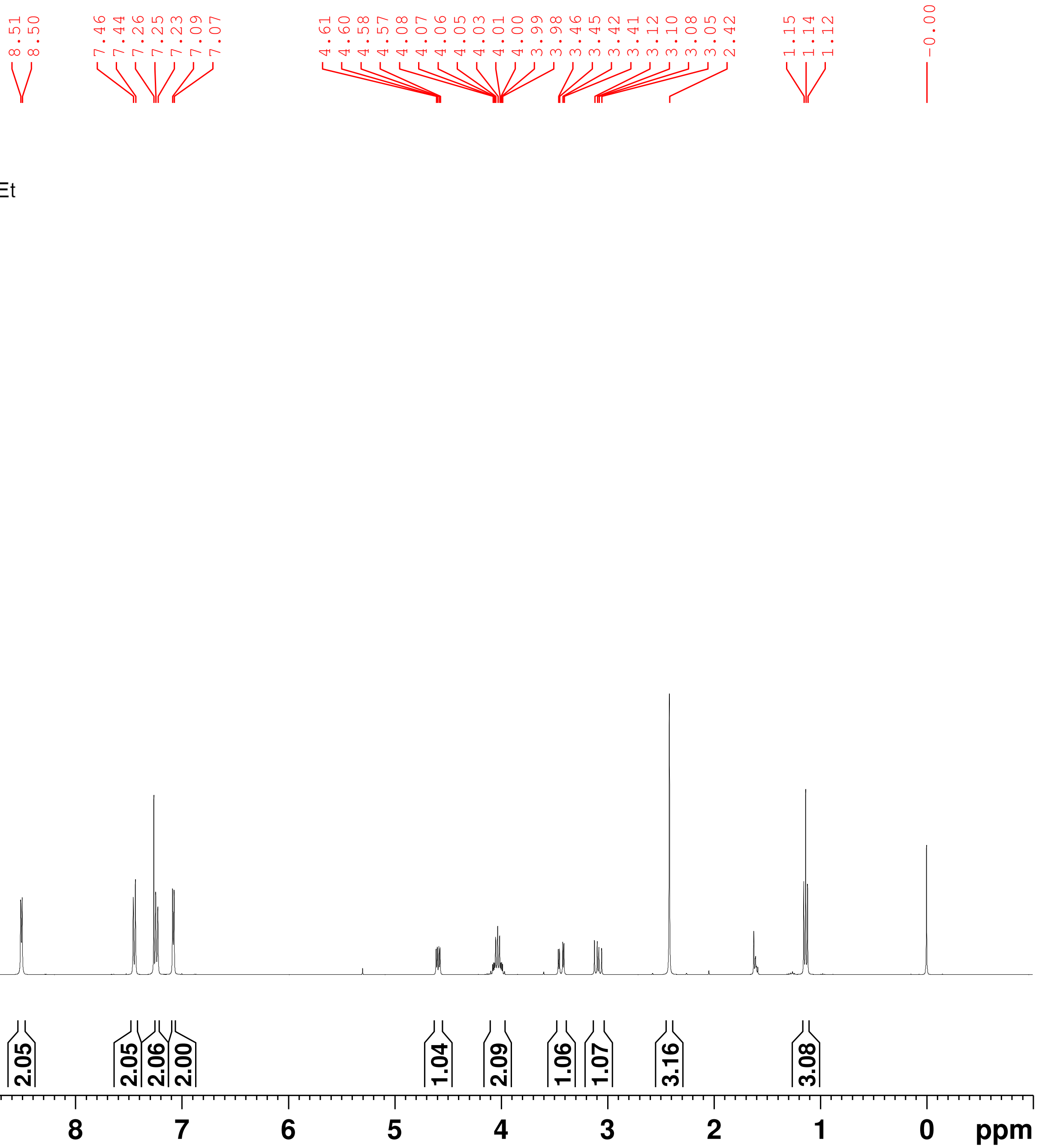


```
Current Data Parameters
NAME        1H_ST-7-223-real
EXPNO                      3
PROCNO                     1

F2 - Acquisition Parameters
Date_               20220521
Time                   11.04 h
INSTRUM               Avance
PROBHD     Z167430_0032 (
PULPROG                 zg30
TD                     65536
SOLVENT                CDCl3
NS                        16
DS                         0
SWH                 8196.722 Hz
FIDRES              0.250144 Hz
AQ                 3.9976959 sec
RG                       101
DW                    61.000 usec
DE                     13.20 usec
TE                     298.0 K
D1                0.10000000 sec
TD0                        1
SFO1             400.3024719 MHz
NUC1                      1H
P0                      4.00 usec
P1                     12.00 usec
PLW1              8.80000019 W

F2 - Processing parameters
SI                     65536
SF               400.3000084 MHz
WDW                       EM
SSB      0
LB                      0.30 Hz
GB       0
PC                      1.00
```

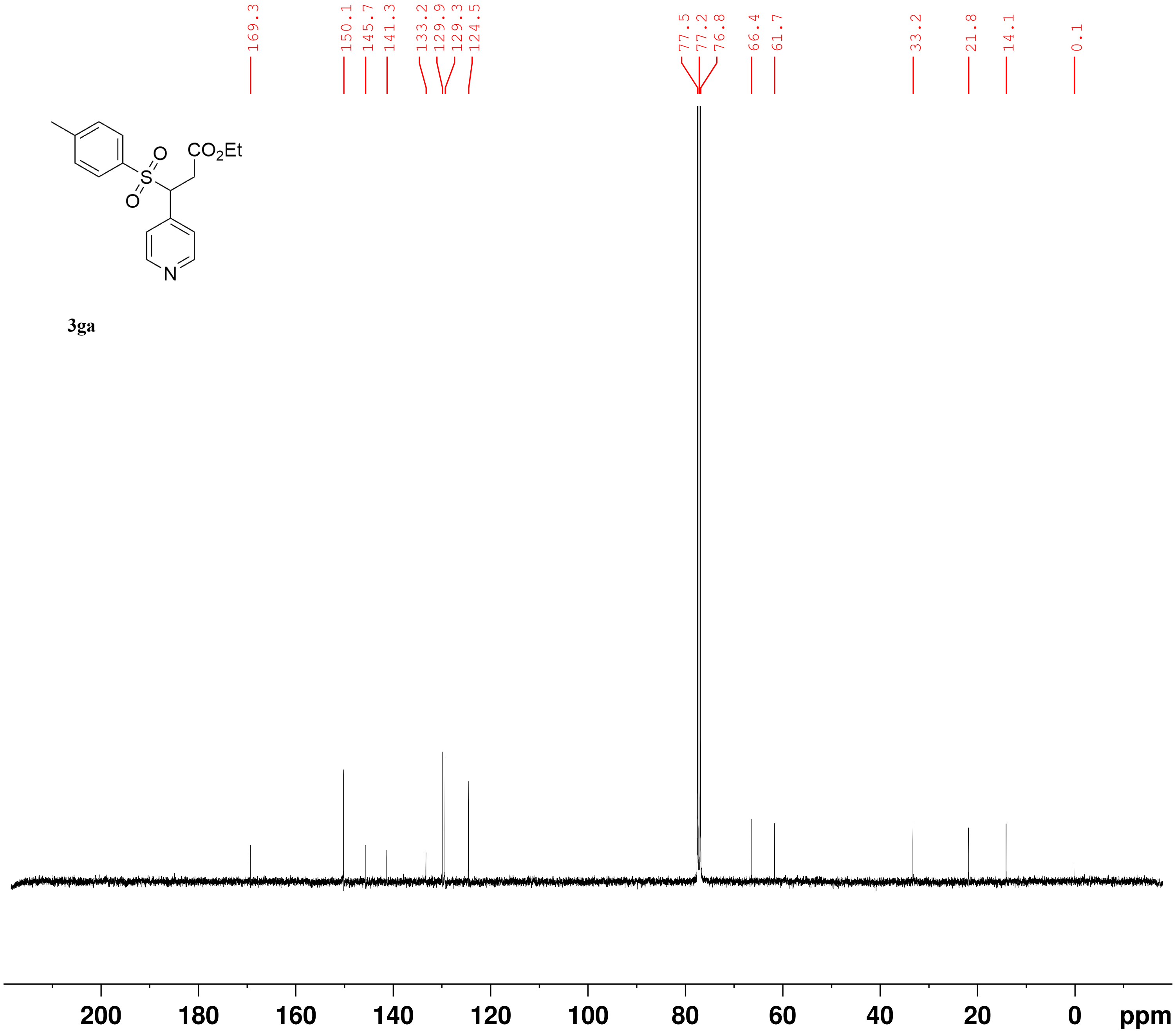


```
Current Data Parameters
NAME      13C_ST-7-223-real
EXPNO                     3
PROCNO                    1

F2 - Acquisition Parameters
Date_              20220521
Time                  11.16 h
INSTRUM              Avance
PROBHD     Z167430_0032 (
PULPROG              zgpg30
TD                    65536
SOLVENT               CDCl3
NS                      128
DS                        4
SWH               23809.523 Hz
FIDRES             0.726609 Hz
AQ                1.3762560 sec
RG                     3.25
DW                   21.000 usec
DE                    19.29 usec
TE                    298.0 K
D1               2.00000000 sec
D11              0.03000000 sec
TD0                       1
SFO1            100.6655806 MHz
NUC1                    13C
P0                     3.33 usec
P1                    10.00 usec
PLW1            39.31399918 W
SFO2            400.3016012 MHz
NUC2                     1H
CPDPRG[2            waltz64
PCPD2                 80.00 usec
PLW2             8.80000019 W
PLW12            0.20176961 W
PLW13            0.10112690 W

F2 - Processing parameters
SI                   131072
SF              100.6555021 MHz
WDW                      EM
SSB        0
LB                     1.00 Hz
GB         0
PC                     1.40
```

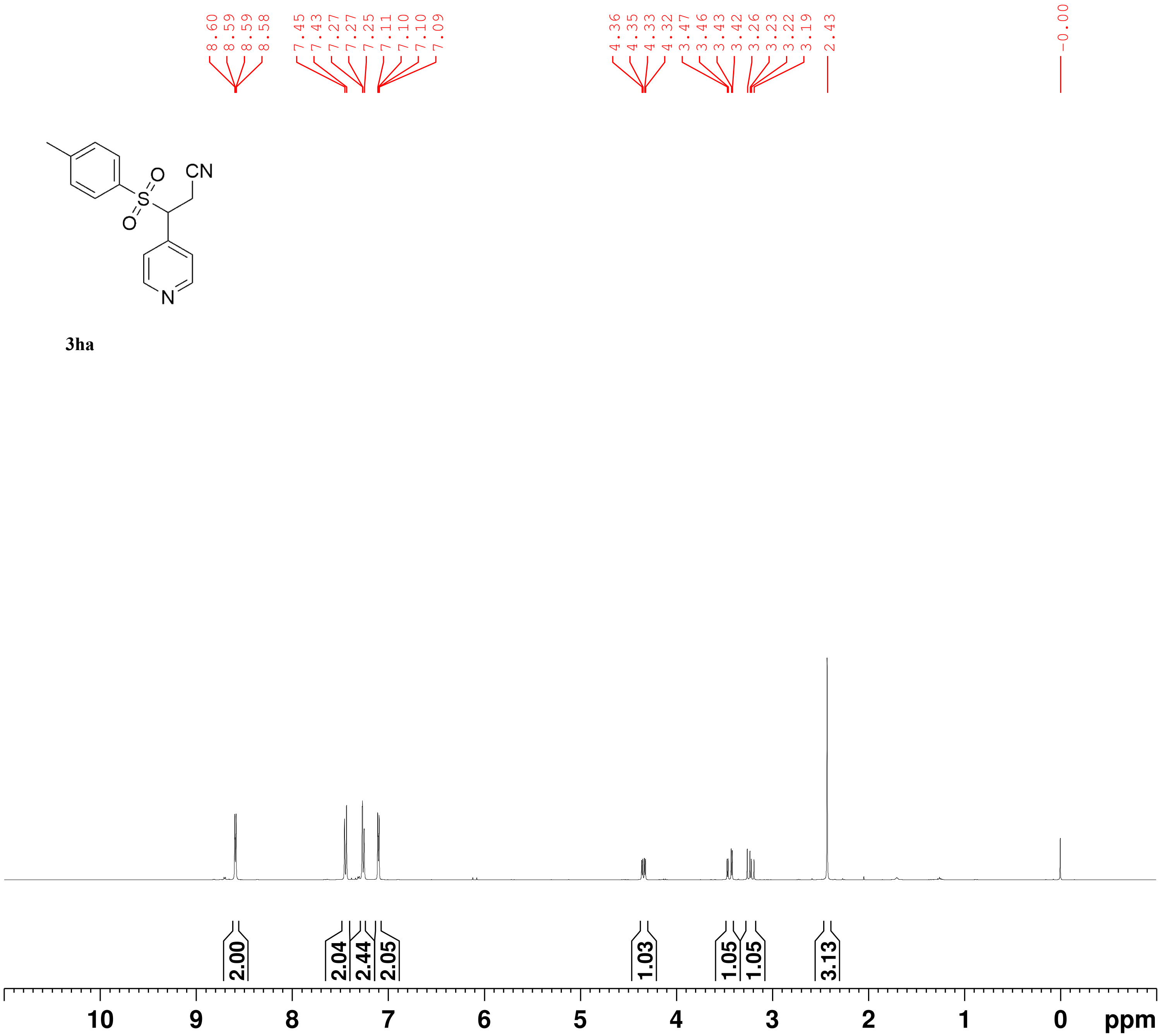

3ha

```
Current Data Parameters
NAME          1H ST-07-249
EXPNO                    3
PROCNO                   1

F2 - Acquisition Parameters
Date_             20220605
Time                 18.38 h
INSTRUM             Avance
PROBHD      Z167430_0032 (
PULPROG               zg30
TD                   65536
SOLVENT              CDCl3
NS                      16
DS                       0
SWH               8196.722 Hz
FIDRES            0.250144 Hz
AQ               3.9976959 sec
RG                     101
DW                  61.000 usec
DE                   13.20 usec
TE                   298.0 K
D1              0.10000000 sec
TD0                      1
SFO1           400.3024719 MHz
NUC1                    1H
P0                    4.00 usec
P1                   12.00 usec
PLW1            8.80000019 W

F2 - Processing parameters
SI                   65536
SF             400.3000070 MHz
WDW                     EM
SSB      0
LB                    0.30 Hz
GB       0
PC                    1.00
```

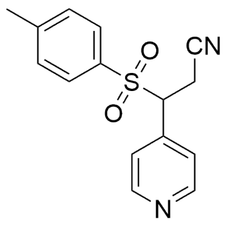

3ha

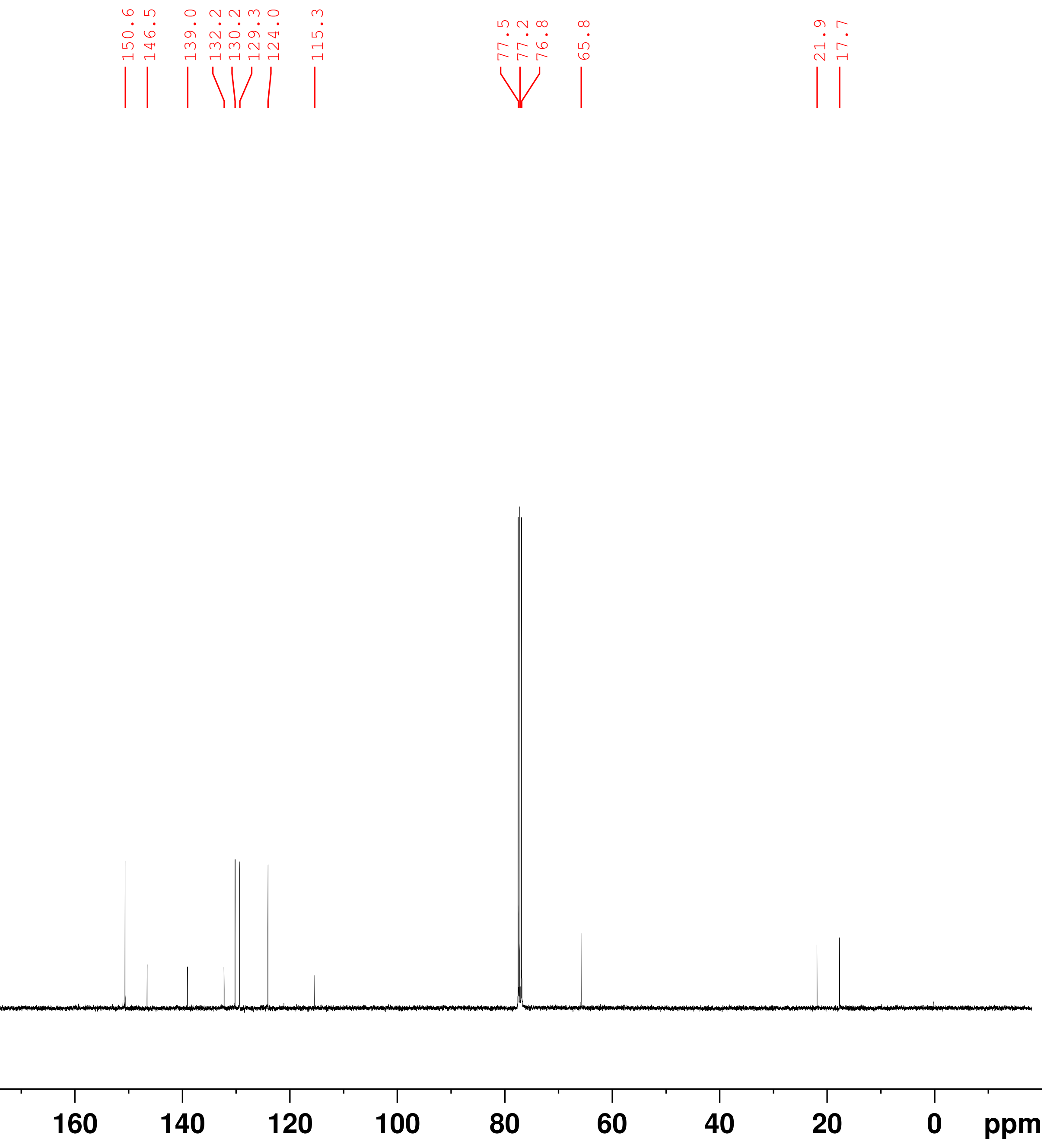

Current Data Parameters
NAME 13C_ST-7-249
EXPNO 2
PROCNO 1

F2 - Acquisition Parameters
Date_ 20220605
Time 18.43 h
INSTRUM Avance
PROBHD Z167430_0032 (
PULPROG zgpg30
TD 65536
SOLVENT CDCl3
NS 64
DS 4
SWH 23809.523 Hz
FIDRES 0.726609 Hz
AQ 1.3762560 sec
RG 3.25
DW 21.000 usec
DE 19.29 usec
TE 298.0 K
D1 3.00000000 sec
D11 0.03000000 sec
TD0 1
SFO1 100.6655806 MHz
NUC1 13C
P0 3.33 usec
P1 10.00 usec
PLW1 39.31399918 W
SFO2 400.3016012 MHz
NUC2 1H
CPDPRG[2 waltz64
PCPD2 80.00 usec
PLW2 8.80000019 W
PLW12 0.20176961 W
PLW13 0.10112690 W

F2 - Processing parameters
SI 131072
SF 100.6555041 MHz
WDW EM
SSB 0
LB 1.00 Hz
GB 0
PC 1.40

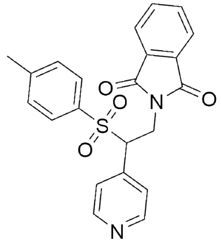

3ia

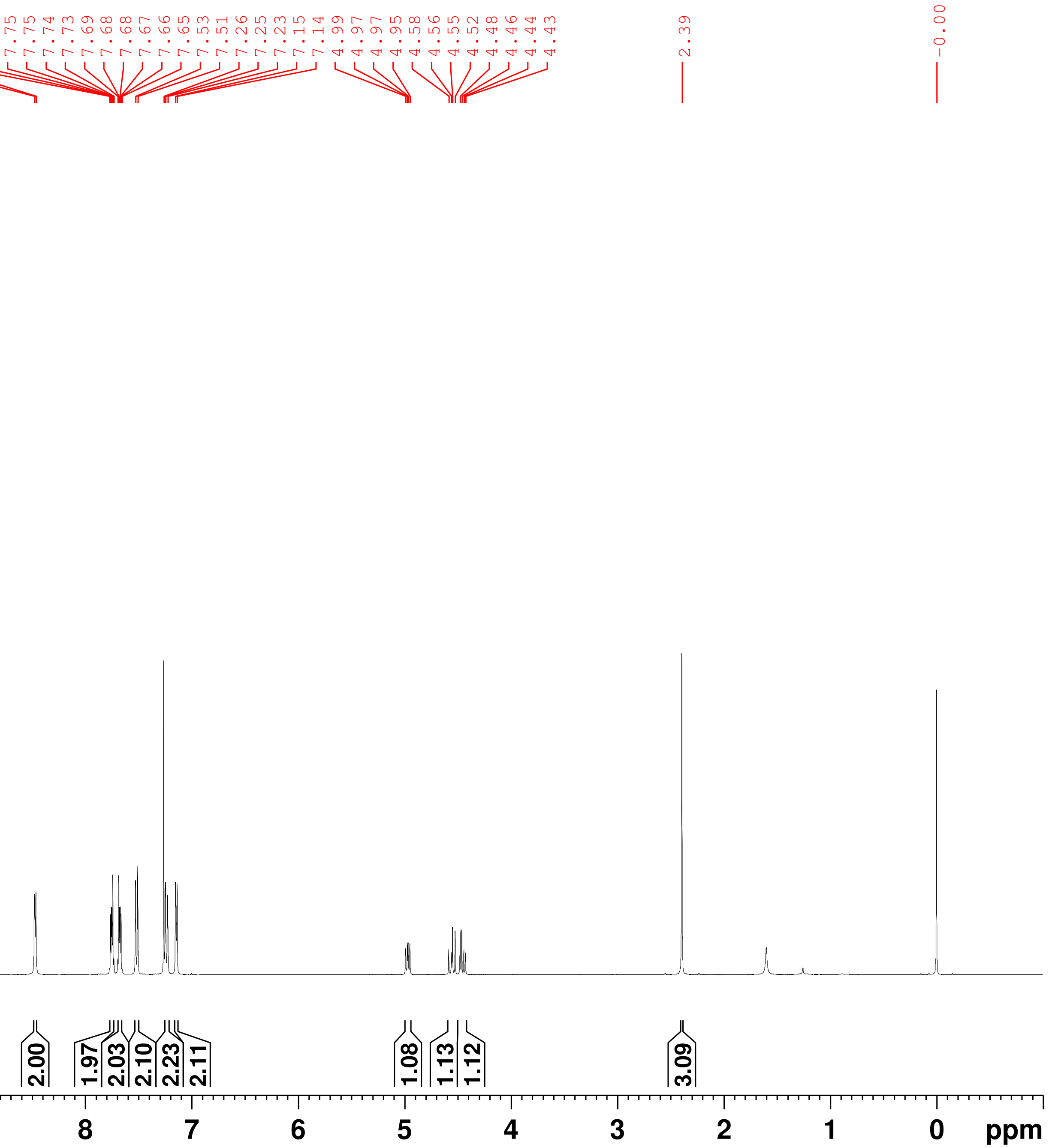


```
Current Data Parameters
NAME      1H ST-07-247-re
EXPNO                   3
PROCNO                  1

F2 - Acquisition Parameters
Date_            20220602
Time                12.26 h
INSTRUM            Avance
PROBHD     Z167430_0032 (
PULPROG              zg30
TD                  65536
SOLVENT             CDCl3
NS                     16
DS                      0
SWH              8196.722 Hz
FIDRES           0.250144 Hz
AQ              3.9976959 sec
RG                    101
DW                 61.000 usec
DE                  13.20 usec
TE                  298.0 K
D1             0.10000000 sec
TD0                     1
SFO1          400.3024719 MHz
NUC1                   1H
P0                   4.00 usec
P1                  12.00 usec
PLW1           8.80000019 W

F2 - Processing parameters
SI                  65536
SF            400.3000087 MHz
WDW                    EM
SSB      0
LB                   0.30 Hz
GB       0
PC                   1.00
```

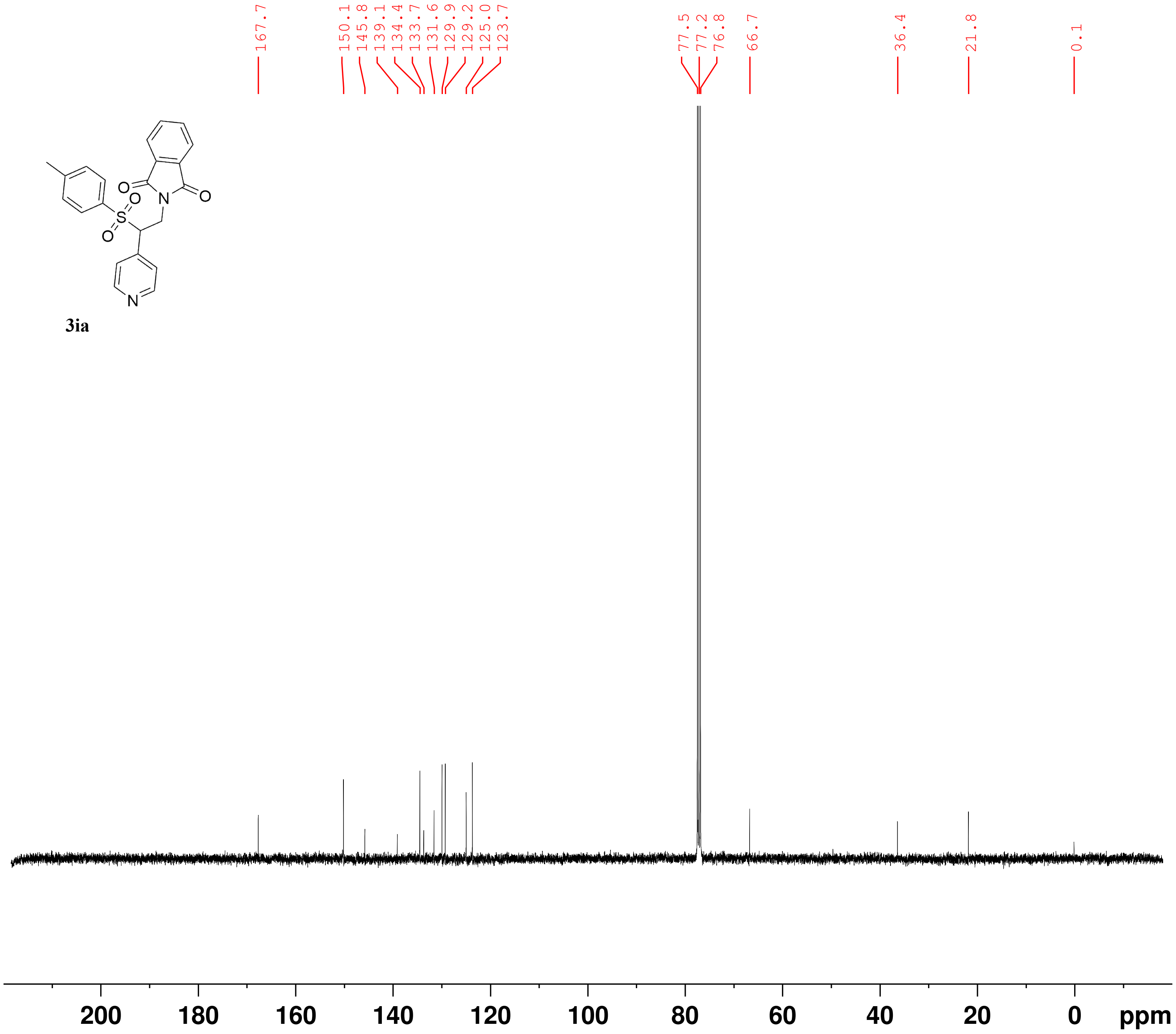

3ia

BRUKER

Current Data Parameters

NAME	13C_ST-7-247
EXPNO	3
PROCNO	1

F2 - Acquisition Parameters

Date_	20220601
Time	17.49 h
INSTRUM	Avance
PROBHD	Z167430_0032 (
PULPROG	zgpg30
TD	65536
SOLVENT	CDCl3
NS	64
DS	4
SWH	23809.523 Hz
FIDRES	0.726609 Hz
AQ	1.3762560 sec
RG	3.25
DW	21.000 usec
DE	19.29 usec
TE	298.0 K
D1	3.00000000 sec
D11	0.03000000 sec
TD0	1
SFO1	100.6655806 MHz
NUC1	13C
P0	3.33 usec
P1	10.00 usec
PLW1	39.31399918 W
SFO2	400.3016012 MHz
NUC2	1H
CPDPRG[2	waltz64
PCPD2	80.00 usec
PLW2	8.80000019 W
PLW12	0.20176961 W
PLW13	0.10112690 W

F2 - Processing parameters

SI	131072
SF	100.6555023 MHz
WDW	EM
SSB	0
LB	1.00 Hz
GB	0
PC	1.40

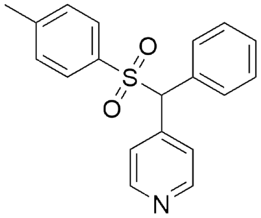

3ja

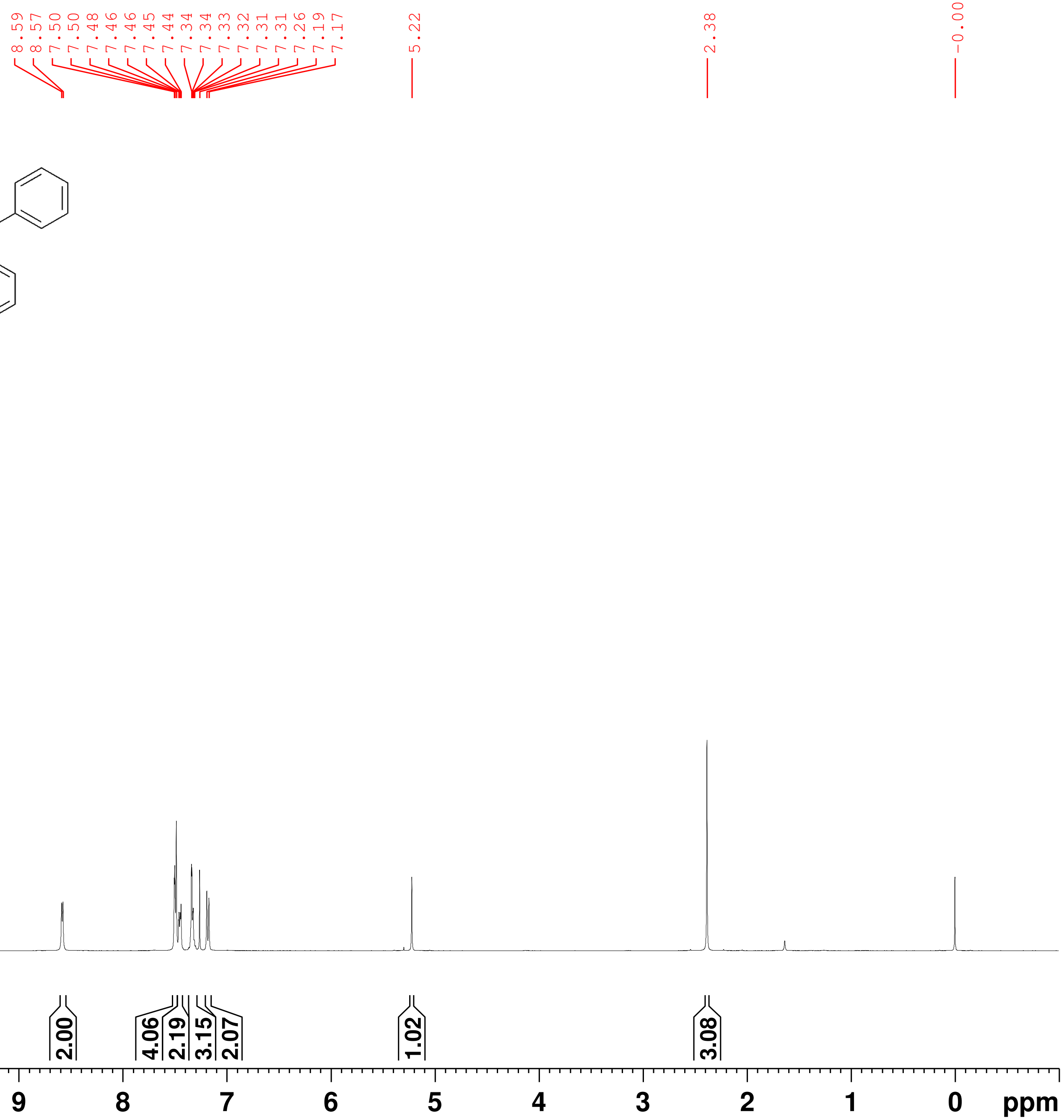

Current Data Parameters
NAME 1H ST-07-251
EXPNO 5
PROCNO 1

F2 - Acquisition Parameters
Date_ 20220605
Time 19.18 h
INSTRUM Avance
PROBHD Z167430_0032 (
PULPROG zg30
TD 65536
SOLVENT CDCl3
NS 16
DS 0
SWH 8196.722 Hz
FIDRES 0.250144 Hz
AQ 3.9976959 sec
RG 101
DW 61.000 usec
DE 13.20 usec
TE 298.0 K
D1 0.10000000 sec
TD0 1
SFO1 400.3024719 MHz
NUC1 1H
P0 4.00 usec
P1 12.00 usec
PLW1 8.80000019 W

F2 - Processing parameters
SI 65536
SF 400.3000092 MHz
WDW EM
SSB 0
LB 0.30 Hz
GB 0
PC 1.00

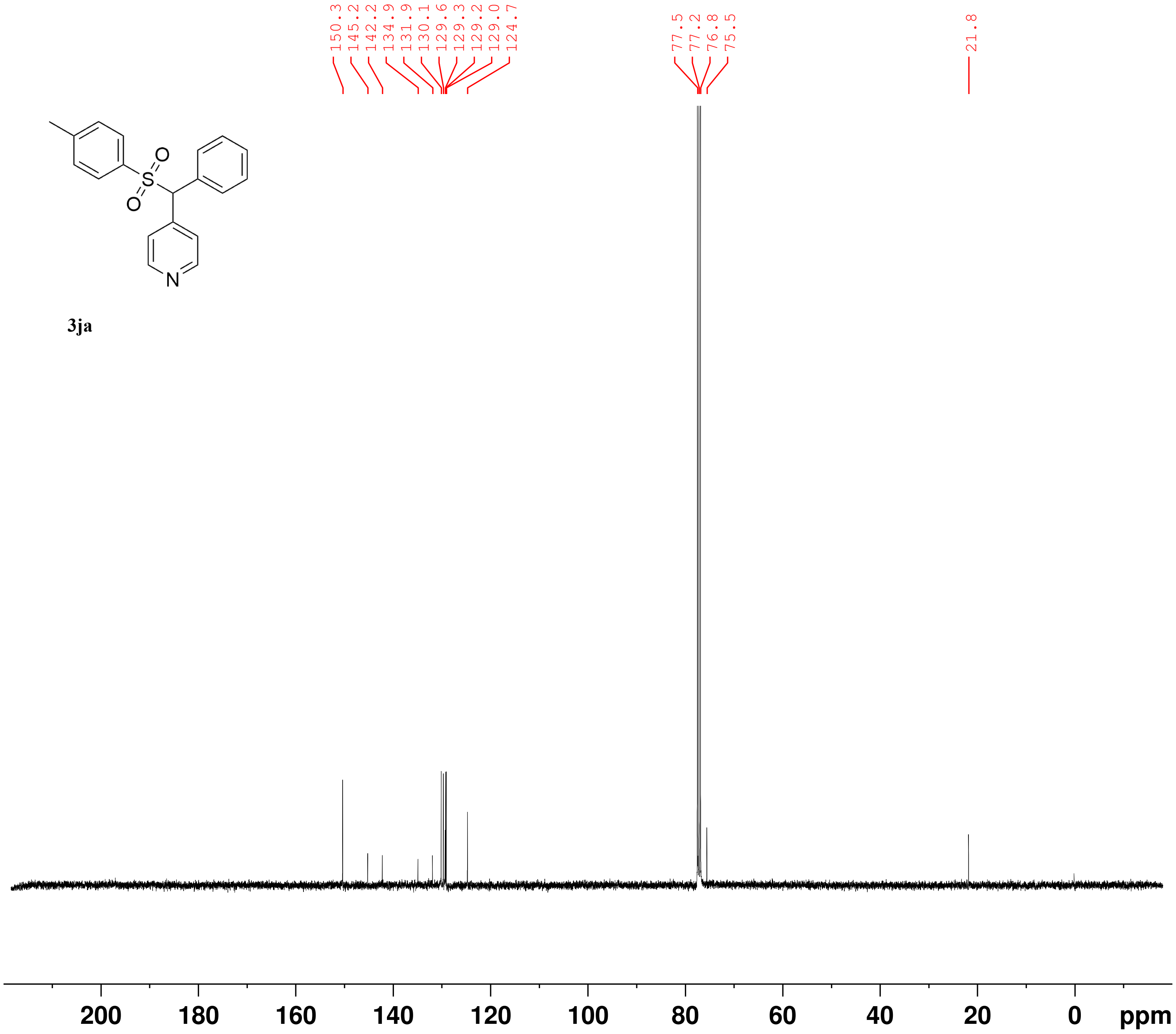

3ja

BRUKER

```
Current Data Parameters
NAME          13C_ST-7-251
EXPNO                    3
PROCNO                   1

F2 - Acquisition Parameters
Date_             20220605
Time                 19.24 h
INSTRUM             Avance
PROBHD     Z167430_0032 (
PULPROG            zgpg30
TD                   65536
SOLVENT              CDCl3
NS                      64
DS                       4
SWH              23809.523 Hz
FIDRES            0.726609 Hz
AQ               1.3762560 sec
RG                    3.25
DW                  21.000 usec
DE                   19.29 usec
TE                   298.0 K
D1              3.00000000 sec
D11             0.03000000 sec
TD0                      1
SFO1           100.6655806 MHz
NUC1                   13C
P0                    3.33 usec
P1                   10.00 usec
PLW1           39.31399918 W
SFO2           400.3016012 MHz
NUC2                    1H
CPDPRG[2           waltz64
PCPD2                80.00 usec
PLW2            8.80000019 W
PLW12           0.20176961 W
PLW13           0.10112690 W

F2 - Processing parameters
SI                  131072
SF             100.6555026 MHz
WDW                     EM
SSB        0
LB                    1.00 Hz
GB         0
PC                    1.40
```

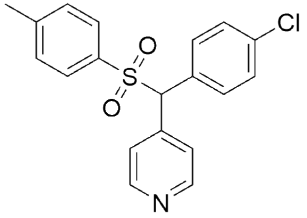

3ka

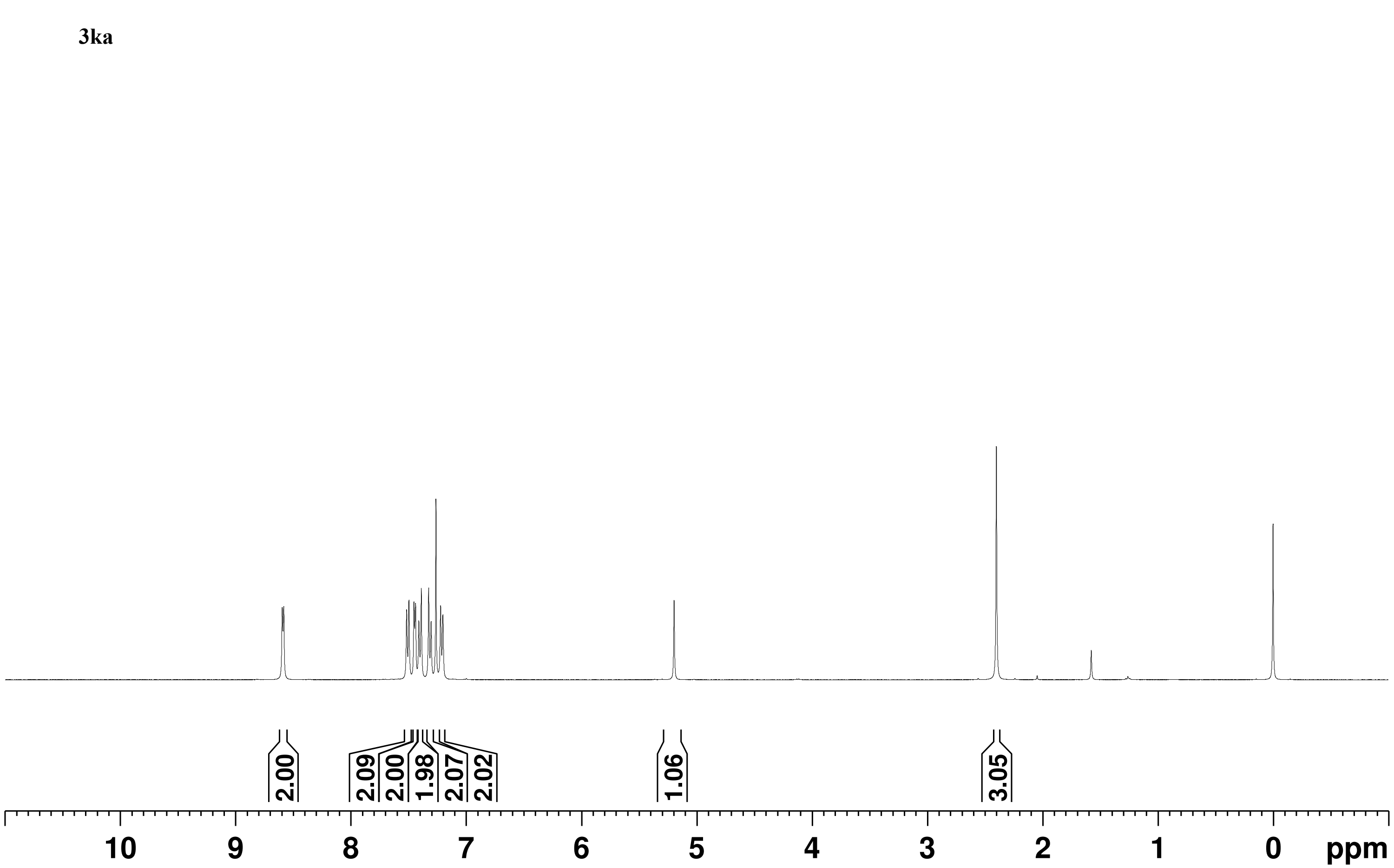


```
Current Data Parameters
NAME         1H ST-07-255
EXPNO                   6
PROCNO                  1

F2 - Acquisition Parameters
Date_            20220612
Time                16.37 h
INSTRUM            Avance
PROBHD    Z167430_0032 (
PULPROG              zg30
TD                  65536
SOLVENT             CDCl3
NS                     16
DS                      0
SWH              8196.722 Hz
FIDRES           0.250144 Hz
AQ              3.9976959 sec
RG                    101
DW                 61.000 usec
DE                  13.20 usec
TE                  298.0 K
D1             0.10000000 sec
TD0                     1
SFO1          400.3024719 MHz
NUC1                   1H
P0                   4.00 usec
P1                  12.00 usec
PLW1           8.80000019 W

F2 - Processing parameters
SI                  65536
SF            400.3000095 MHz
WDW                    EM
SSB      0
LB                   0.30 Hz
GB       0
PC                   1.00
```

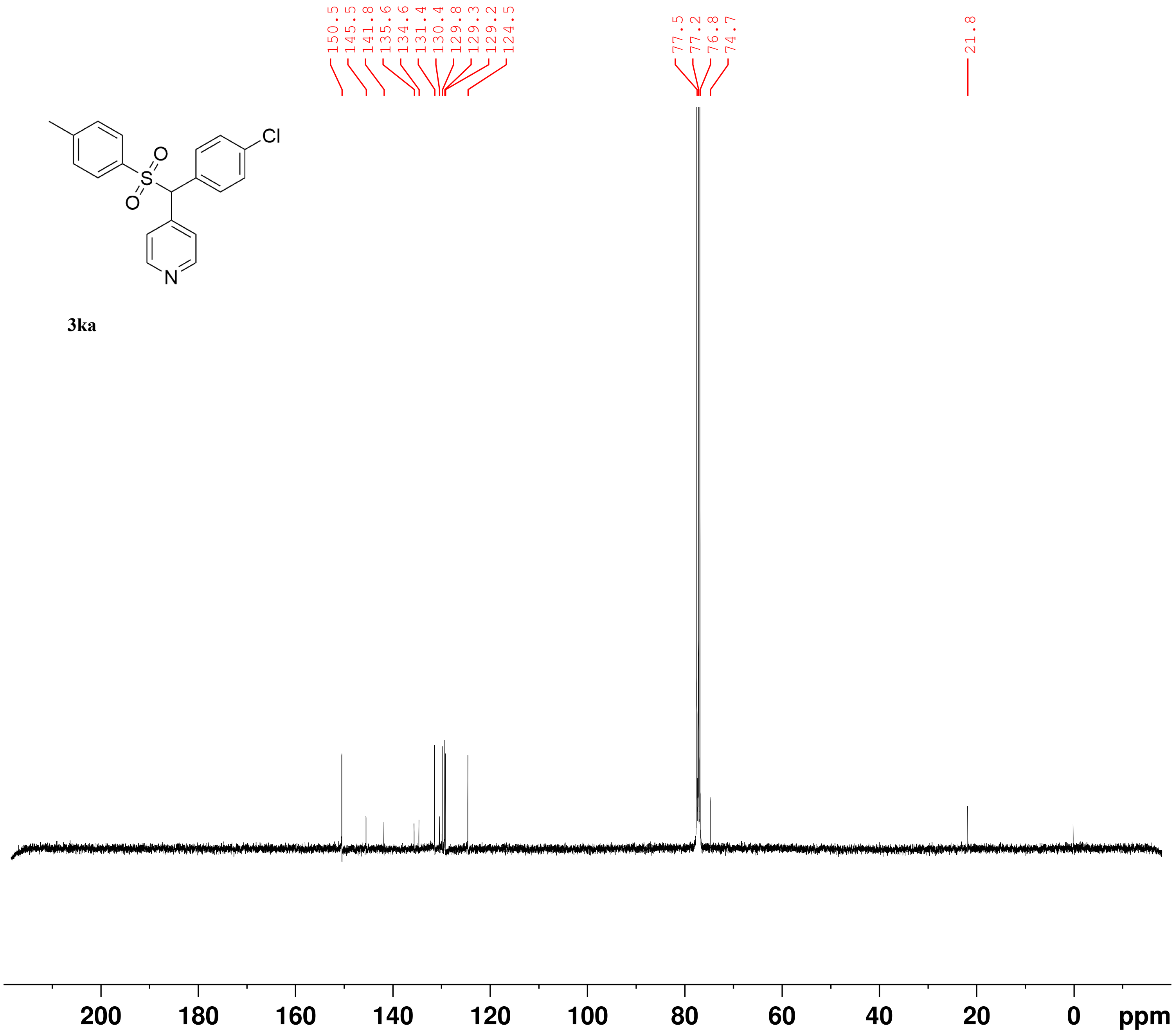

3ka

```
Current Data Parameters
NAME       13C_ST-7-255-re
EXPNO                    3
PROCNO                   1

F2 - Acquisition Parameters
Date_             20220612
Time                 17.04 h
INSTRUM             Avance
PROBHD     Z167430_0032 (
PULPROG            zgpg30
TD                   65536
SOLVENT              CDCl3
NS                     250
DS                       4
SWH              23809.523 Hz
FIDRES            0.726609 Hz
AQ               1.3762560 sec
RG                    3.25
DW                  21.000 usec
DE                   19.29 usec
TE                   298.0 K
D1              3.00000000 sec
D11             0.03000000 sec
TD0                      1
SFO1           100.6655806 MHz
NUC1                   13C
P0                    3.33 usec
P1                   10.00 usec
PLW1           39.31399918 W
SFO2           400.3016012 MHz
NUC2                    1H
CPDPRG[2           waltz64
PCPD2                80.00 usec
PLW2            8.80000019 W
PLW12           0.20176961 W
PLW13           0.10112690 W

F2 - Processing parameters
SI                  131072
SF             100.6555019 MHz
WDW                     EM
SSB        0
LB                    1.00 Hz
GB         0
PC                    1.40
```

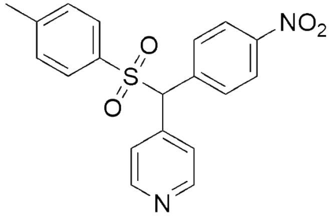

3la

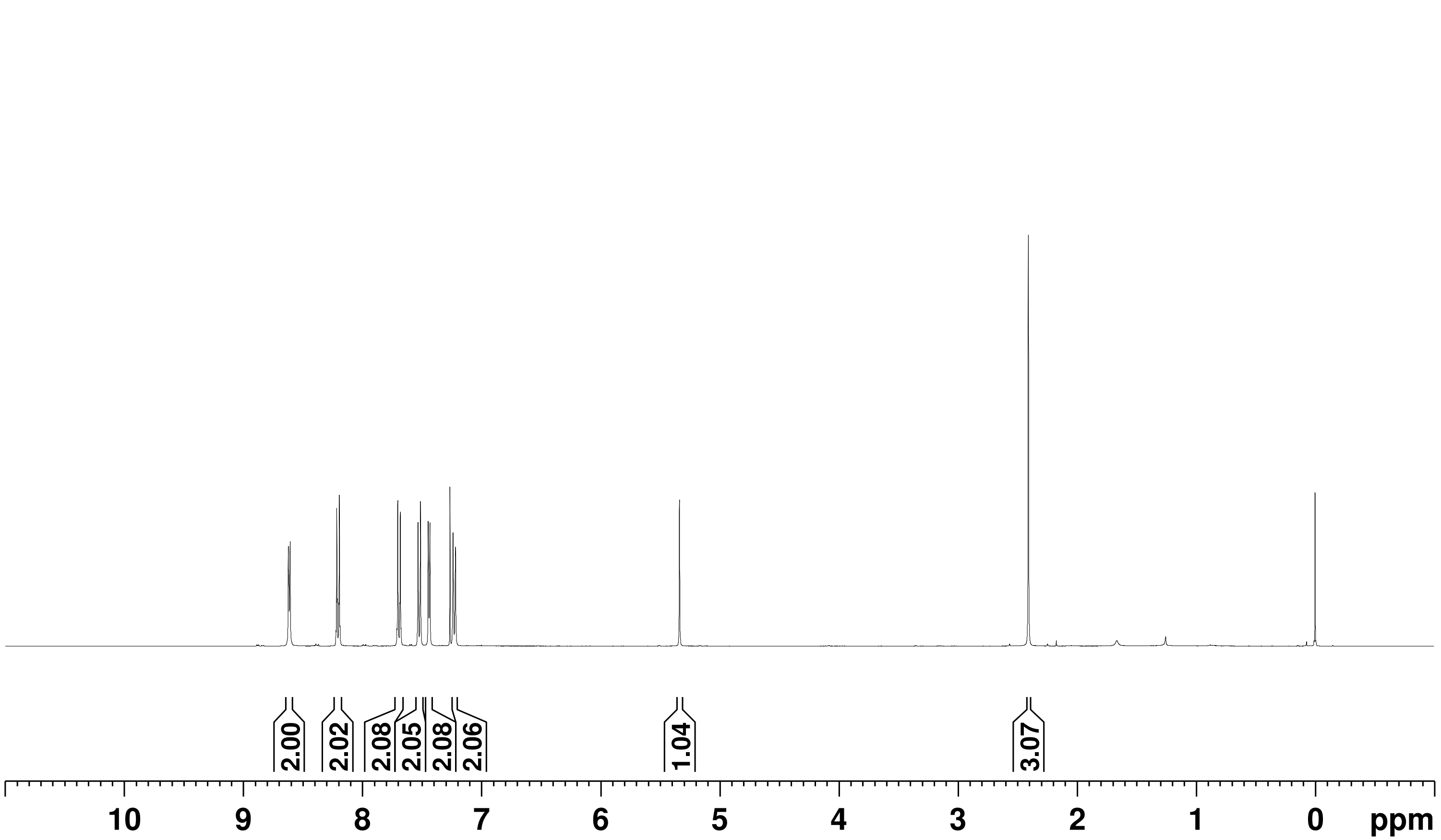


```
Current Data Parameters
NAME          1H ST-07-231-re2
EXPNO                        3
PROCNO                       1

F2 - Acquisition Parameters
Date_                 20220602
Time                    9.07 h
INSTRUM                 Avance
PROBHD       Z167430_0032 (
PULPROG                   zg30
TD                       65536
SOLVENT                  CDCl3
NS                          16
DS                           0
SWH                   8196.722 Hz
FIDRES                0.250144 Hz
AQ                   3.9976959 sec
RG                         101
DW                      61.000 usec
DE                       13.20 usec
TE                       298.0 K
D1                  0.10000000 sec
TD0                          1
SFO1               400.3024719 MHz
NUC1                        1H
P0                        4.00 usec
P1                       12.00 usec
PLW1                8.80000019 W

F2 - Processing parameters
SI                       65536
SF                 400.3000081 MHz
WDW                         EM
SSB       0
LB                        0.30 Hz
GB        0
PC                        1.00
```

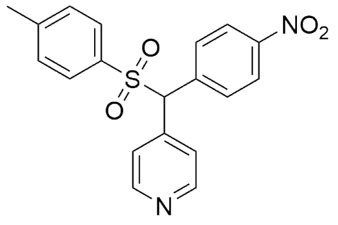

3la

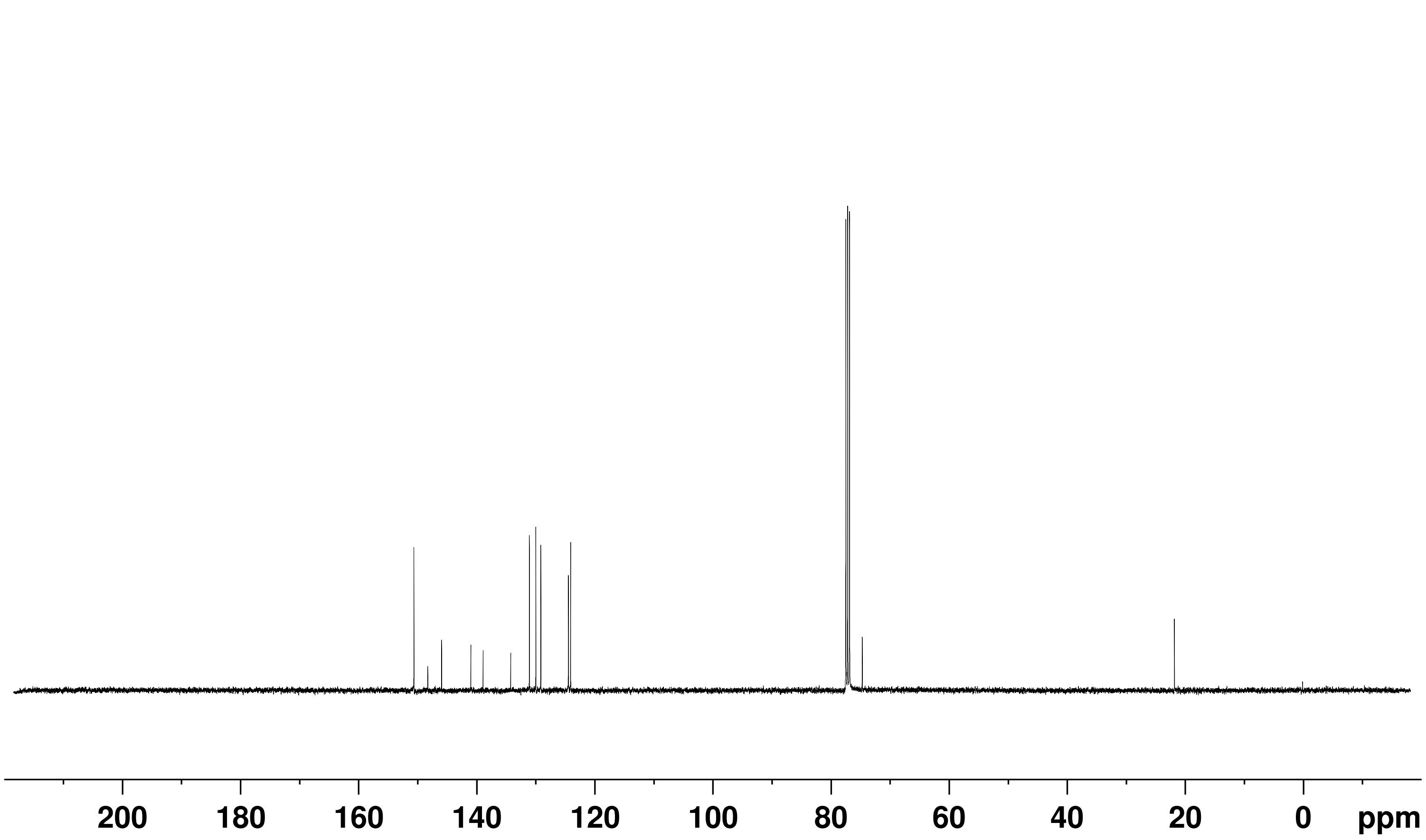


```
Current Data Parameters
NAME          13C_ST-7-231
EXPNO                    1
PROCNO                   1

F2 - Acquisition Parameters
Date_             20220601
Time                 18.08 h
INSTRUM             Avance
PROBHD     Z167430_0032 (
PULPROG            zgpg30
TD                   65536
SOLVENT              CDCl3
NS                      64
DS                       4
SWH              23809.523 Hz
FIDRES            0.726609 Hz
AQ               1.3762560 sec
RG                    3.25
DW                  21.000 usec
DE                   19.29 usec
TE                   298.0 K
D1              3.00000000 sec
D11             0.03000000 sec
TD0                      1
SFO1           100.6655806 MHz
NUC1                   13C
P0                    3.33 usec
P1                   10.00 usec
PLW1           39.31399918 W
SFO2           400.3016012 MHz
NUC2                    1H
CPDPRG[2           waltz64
PCPD2                80.00 usec
PLW2            8.80000019 W
PLW12           0.20176961 W
PLW13           0.10112690 W

F2 - Processing parameters
SI                  131072
SF             100.6555034 MHz
WDW                     EM
SSB       0
LB                    1.00 Hz
GB        0
PC                    1.40
```

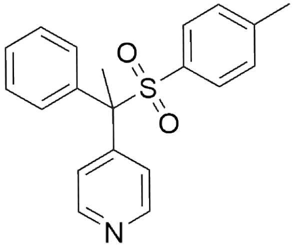

3ma

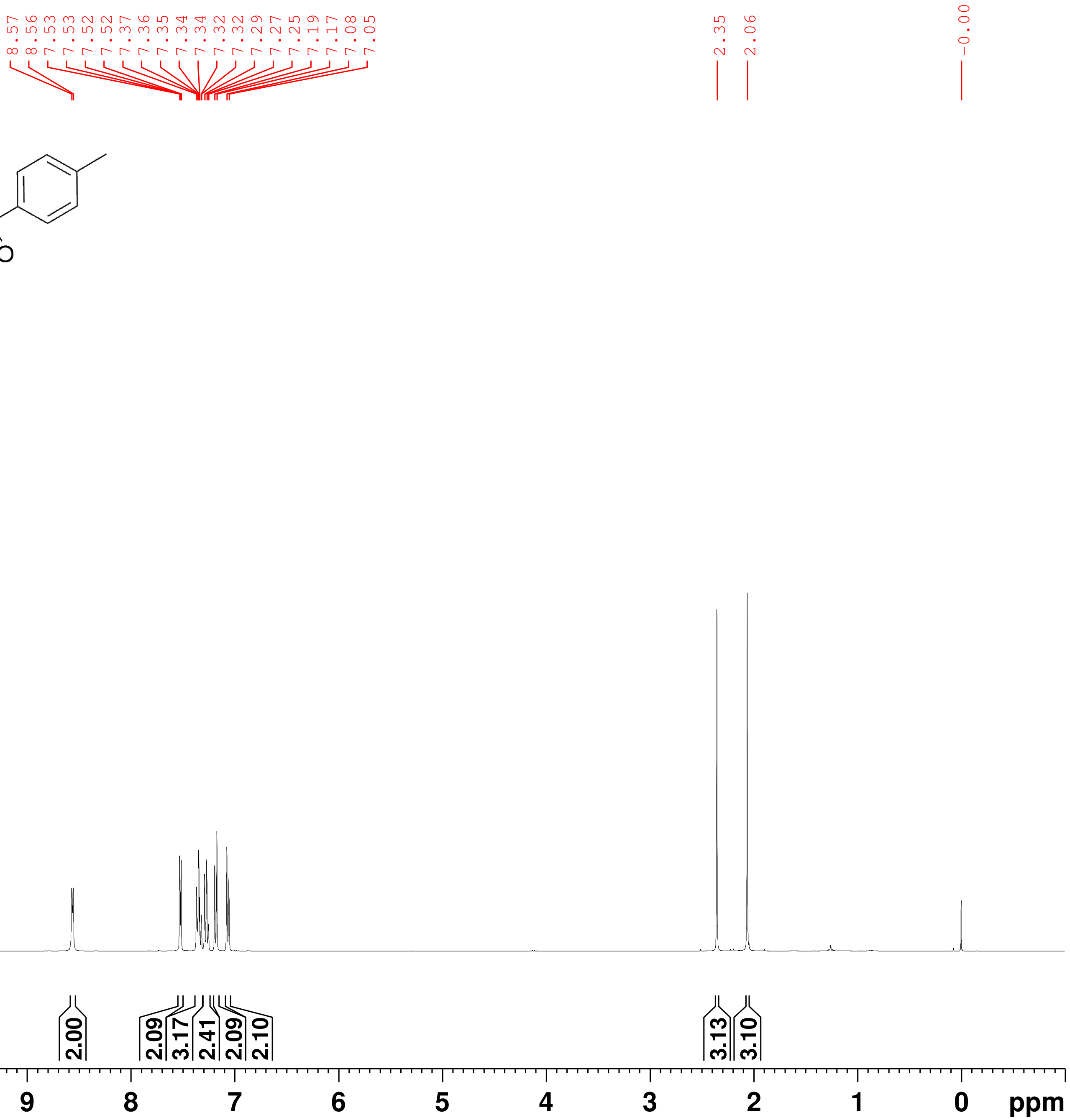


```
Current Data Parameters
NAME          1H_ST-8-113
EXPNO                   3
PROCNO                  1

F2 - Acquisition Parameters
Date_            20221201
Time                 9.17 h
INSTRUM            Avance
PROBHD    Z167430_0032 (
PULPROG              zg30
TD                  65536
SOLVENT             CDCl3
NS                     16
DS                      0
SWH              8196.722 Hz
FIDRES           0.250144 Hz
AQ              3.9976959 sec
RG                    101
DW                 61.000 usec
DE                  13.20 usec
TE                  298.0 K
D1             0.10000000 sec
TD0                     1
SFO1          400.3024719 MHz
NUC1                   1H
P0                   4.00 usec
P1                  12.00 usec
PLW1           8.80000019 W

F2 - Processing parameters
SI                  65536
SF            400.3000069 MHz
WDW                    EM
SSB      0
LB                   0.30 Hz
GB       0
PC                   1.00
```

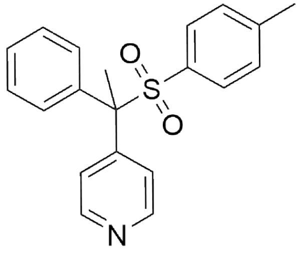

3ma

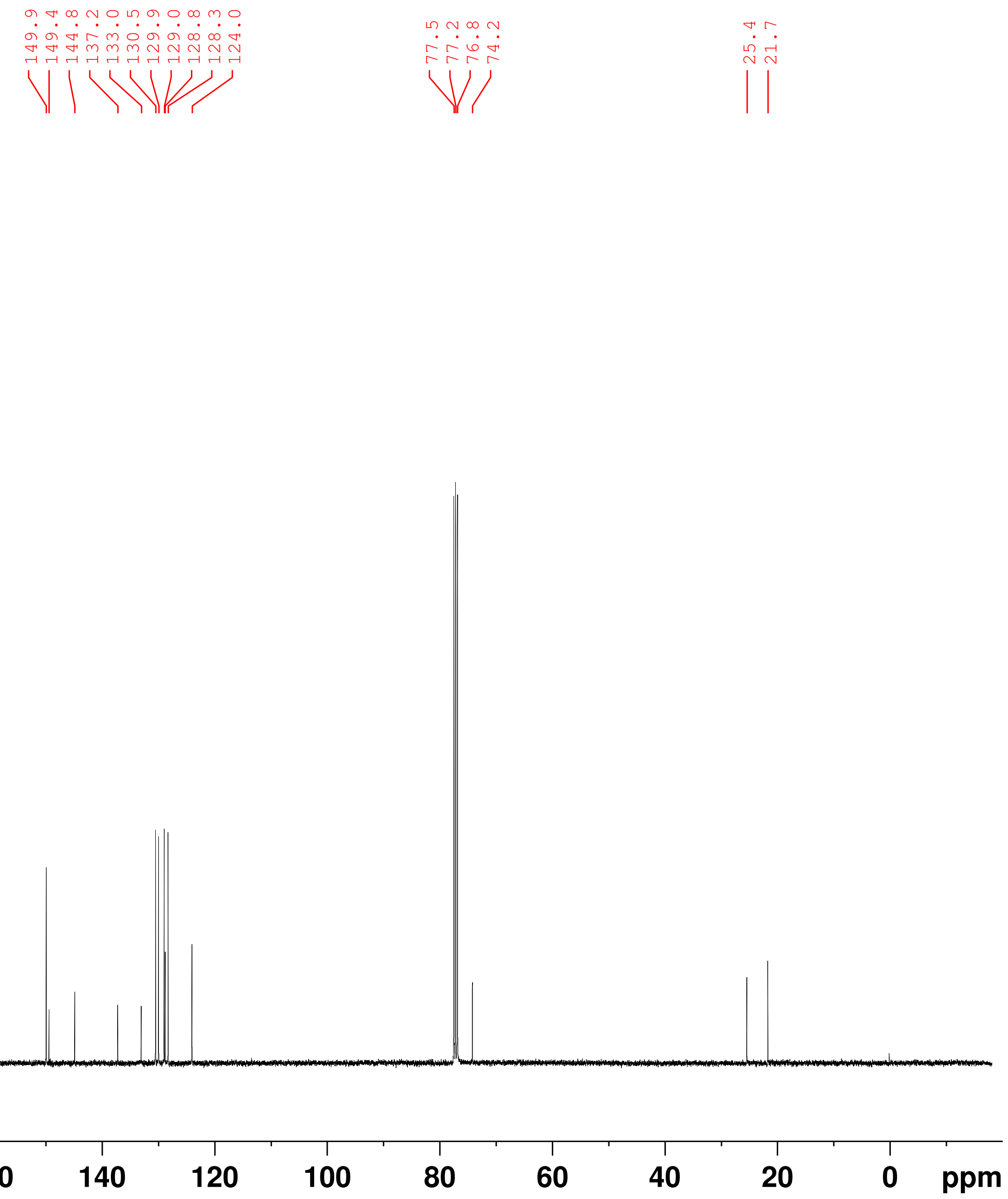

BRUKER

Current Data Parameters
NAME 13C_ST-8-113
EXPNO 1
PROCNO 1

F2 - Acquisition Parameters
Date_ 20221201
Time 9.22 h
INSTRUM Avance
PROBHD Z167430_0032 (
PULPROG zgpg30
TD 65536
SOLVENT CDCl3
NS 64
DS 4
SWH 23809.523 Hz
FIDRES 0.726609 Hz
AQ 1.3762560 sec
RG 3.25
DW 21.000 usec
DE 19.29 usec
TE 298.0 K
D1 3.00000000 sec
D11 0.03000000 sec
TD0 1
SFO1 100.6655806 MHz
NUC1 13C
P0 3.33 usec
P1 10.00 usec
PLW1 39.31399918 W
SFO2 400.3016012 MHz
NUC2 1H
CPDPRG[2 waltz64
PCPD2 80.00 usec
PLW2 8.80000019 W
PLW12 0.20176961 W
PLW13 0.10112690 W

F2 - Processing parameters
SI 131072
SF 100.6555044 MHz
WDW EM
SSB 0
LB 1.00 Hz
GB 0
PC 1.40

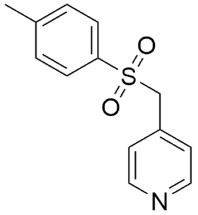

3na

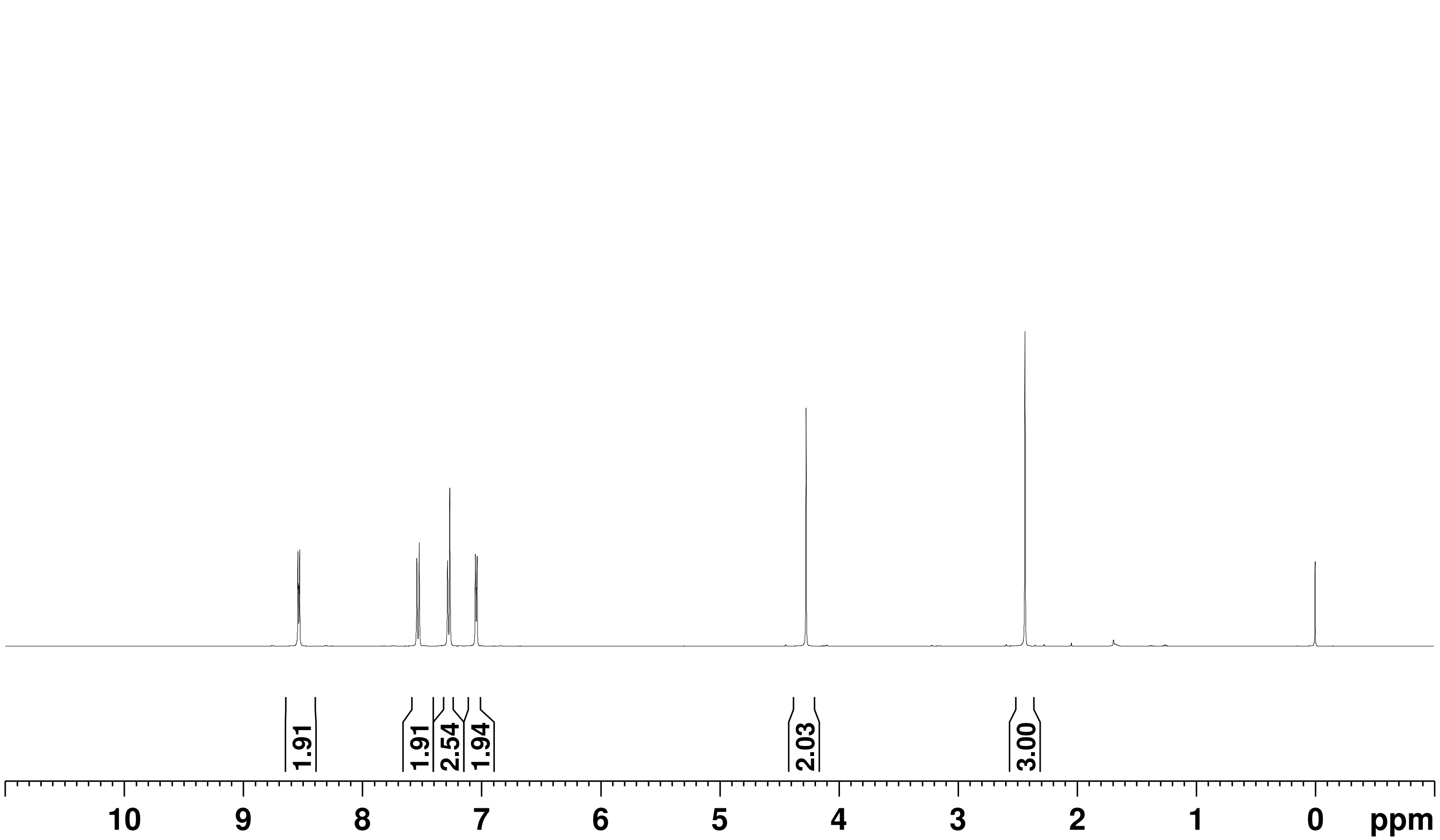

Current Data Parameters	
NAME	1H_JH-1-21
EXPNO	2
PROCNO	1
F2 - Acquisition Parameters	
Date_	20220705
Time	9.20 h
INSTRUM	Avance
PROBHD	Z167430_0032 (
PULPROG	zg30
TD	65536
SOLVENT	CDCl3
NS	16
DS	0
SWH	8196.722 Hz
FIDRES	0.250144 Hz
AQ	3.9976959 sec
RG	101
DW	61.000 usec
DE	13.20 usec
TE	298.0 K
D1	0.10000000 sec
TD0	1
SFO1	400.3024719 MHz
NUC1	1H
P0	4.00 usec
P1	12.00 usec
PLW1	8.80000019 W
F2 - Processing parameters	
SI	65536
SF	400.3000074 MHz
WDW	EM
SSB	0
LB	0.30 Hz
GB	0
PC	1.00

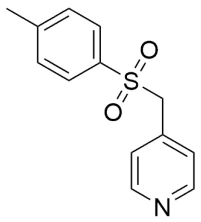

3na

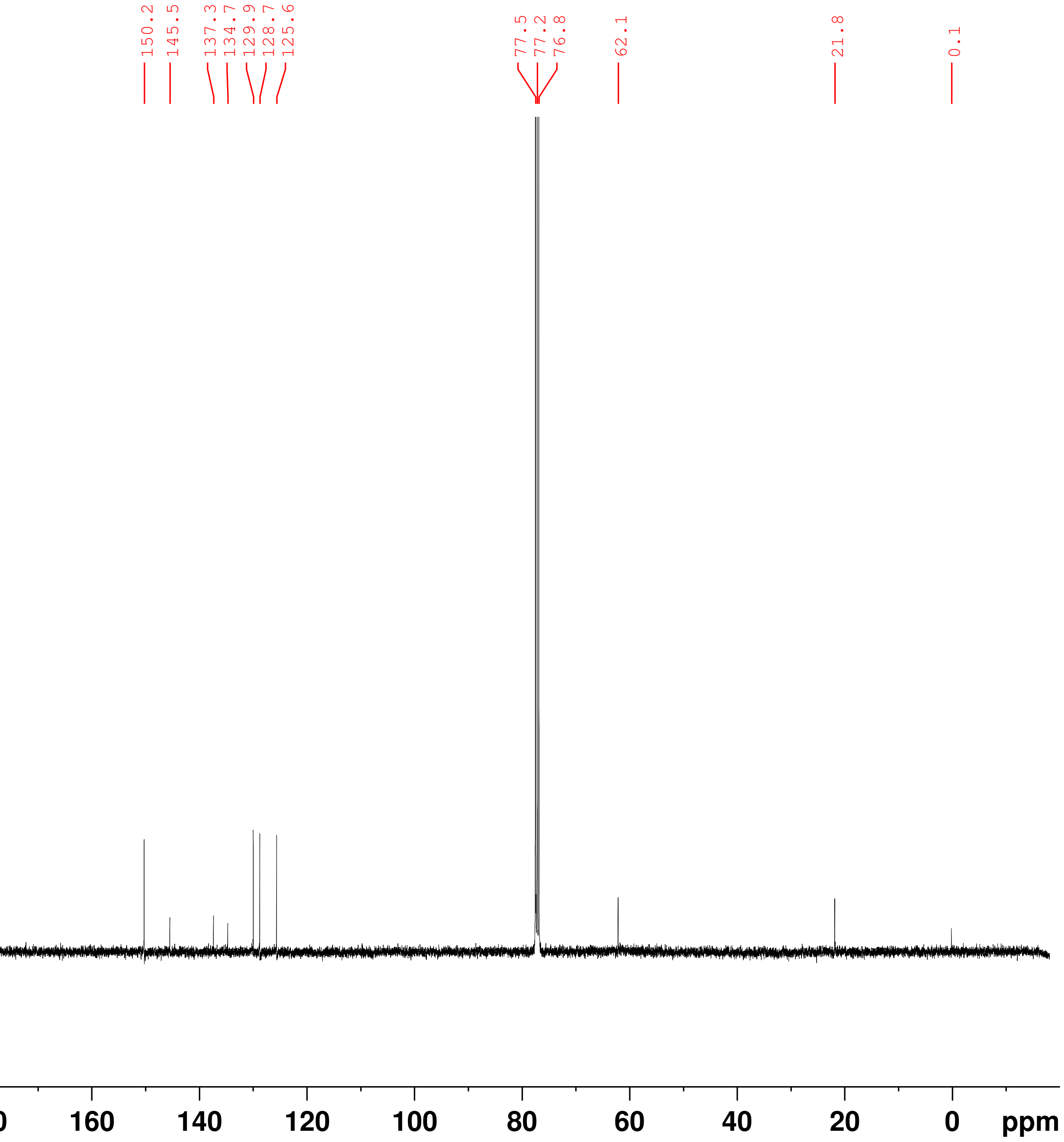

Current Data Parameters
NAME 13C_ST-7-187
EXPNO 5
PROCNO 1

F2 - Acquisition Parameters
Date_ 20220508
Time 15.02 h
INSTRUM Avance
PROBHD Z167430_0032 (
PULPROG zgpg30
TD 65536
SOLVENT CDCl3
NS 128
DS 4
SWH 23809.523 Hz
FIDRES 0.726609 Hz
AQ 1.3762560 sec
RG 3.25
DW 21.000 usec
DE 19.29 usec
TE 298.0 K
D1 3.00000000 sec
D11 0.03000000 sec
TD0 1
SFO1 100.6655806 MHz
NUC1 13C
P0 3.33 usec
P1 10.00 usec
PLW1 39.31399918 W
SFO2 400.3016012 MHz
NUC2 1H
CPDPRG[2 waltz64
PCPD2 80.00 usec
PLW2 8.80000019 W
PLW12 0.20176961 W
PLW13 0.10112690 W

F2 - Processing parameters
SI 131072
SF 100.6555019 MHz
WDW EM
SSB 0
LB 1.00 Hz
GB 0
PC 1.40

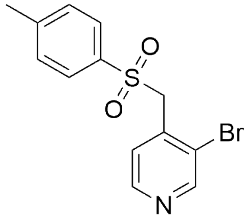

3oa

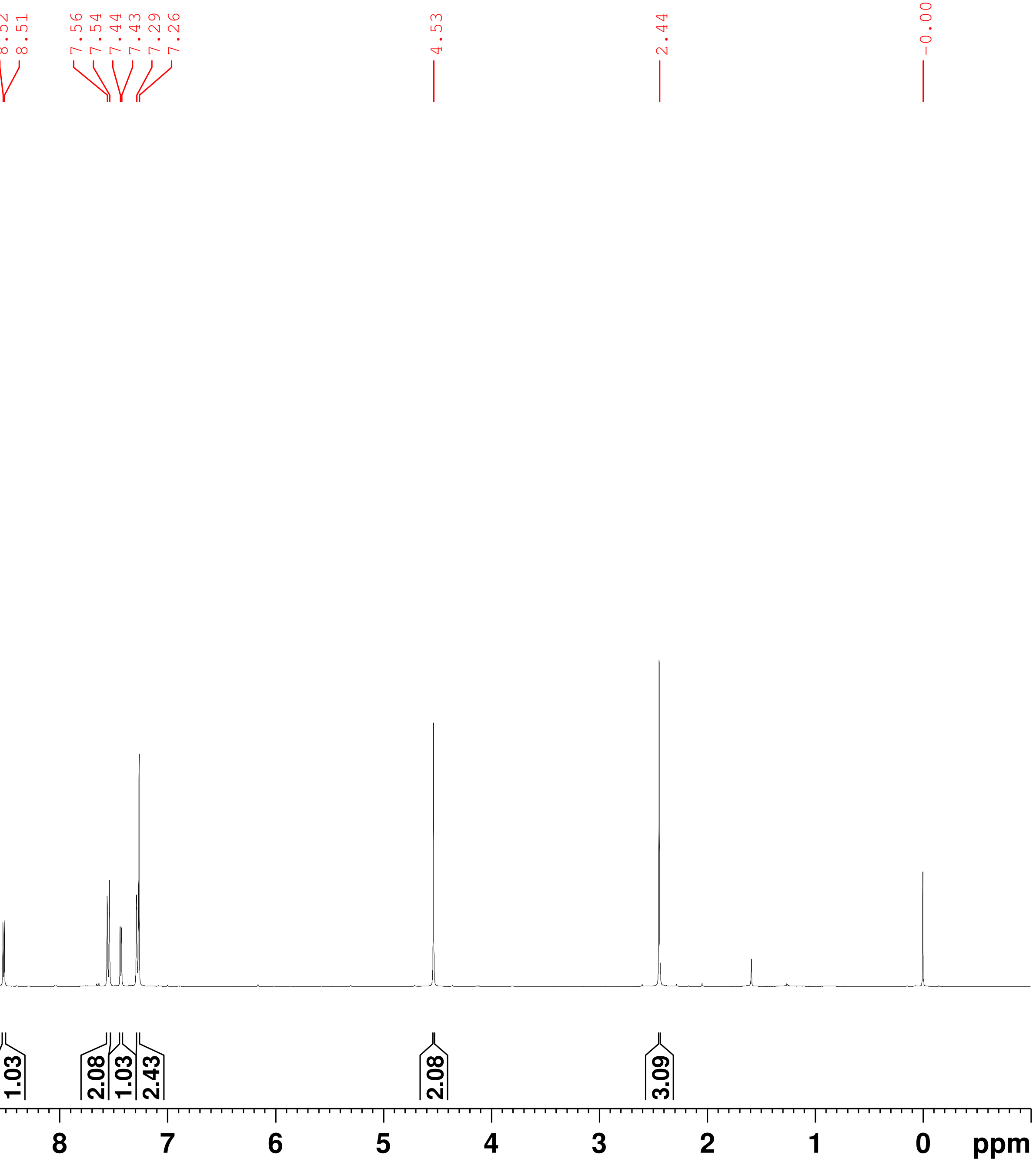

Current Data Parameters

NAME	1H_ST-7-241
EXPNO	1
PROCNO	1

F2 - Acquisition Parameters

Date_	20220531
Time	17.32 h
INSTRUM	Avance
PROBHD	Z167430_0032 (
PULPROG	zg30
TD	65536
SOLVENT	CDCl3
NS	16
DS	0
SWH	8196.722 Hz
FIDRES	0.250144 Hz
AQ	3.9976959 sec
RG	101
DW	61.000 usec
DE	13.20 usec
TE	298.0 K
D1	0.10000000 sec
TD0	1
SFO1	400.3024719 MHz
NUC1	1H
P0	4.00 usec
P1	12.00 usec
PLW1	8.80000019 W

F2 - Processing parameters

SI	65536
SF	400.3000086 MHz
WDW	EM
SSB	0
LB	0.30 Hz
GB	0
PC	1.00

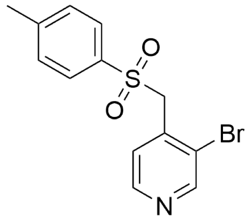

3oa

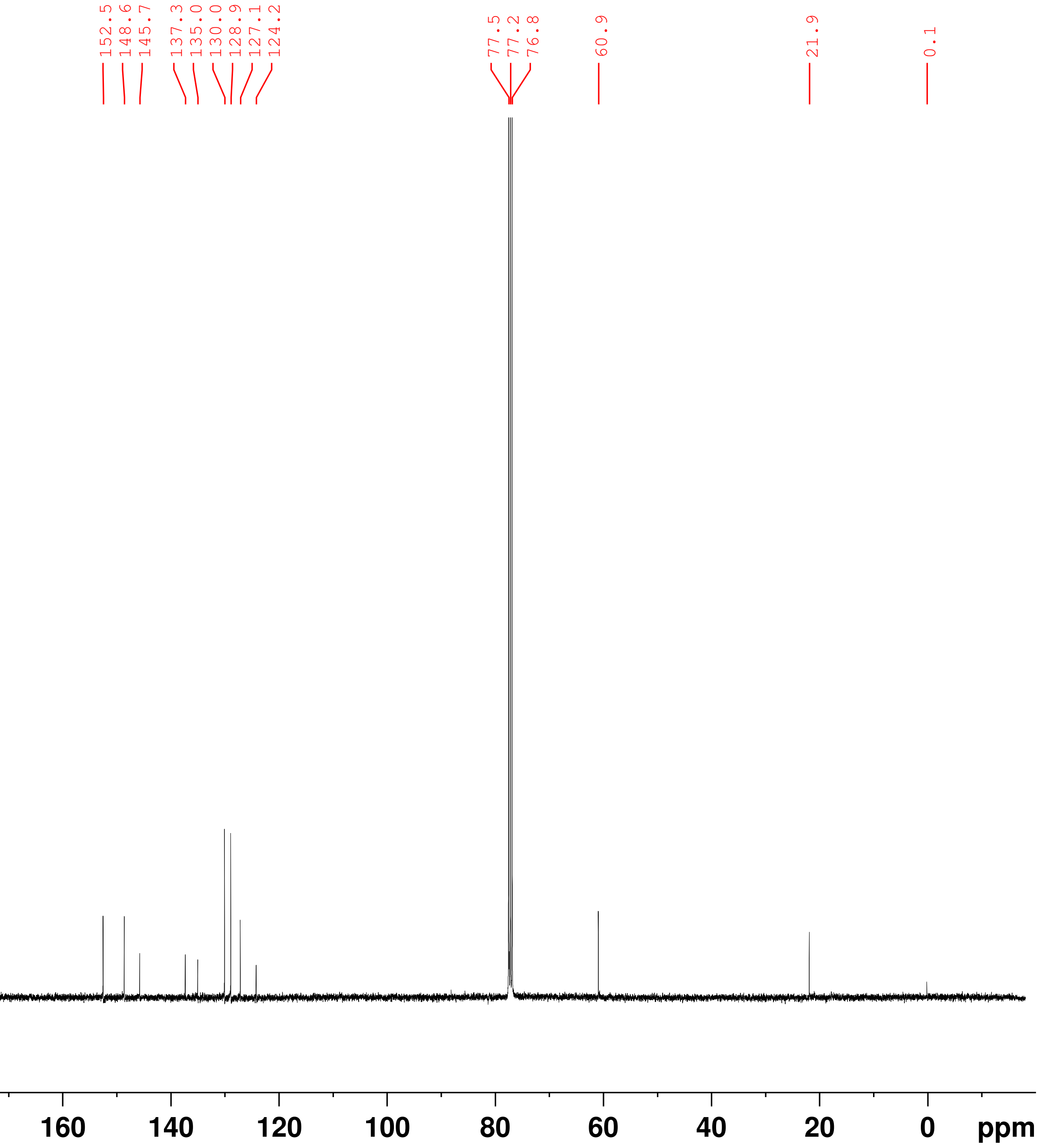

Current Data Parameters
NAME 13C_ST-7-241
EXPNO 1
PROCNO 1

F2 - Acquisition Parameters
Date_ 20220531
Time 17.48 h
INSTRUM Avance
PROBHD Z167430_0032 (
PULPROG zgpg30
TD 65536
SOLVENT CDCl3
NS 128
DS 4
SWH 23809.523 Hz
FIDRES 0.726609 Hz
AQ 1.3762560 sec
RG 3.25
DW 21.000 usec
DE 19.29 usec
TE 298.0 K
D1 3.00000000 sec
D11 0.03000000 sec
TD0 1
SFO1 100.6655806 MHz
NUC1 13C
P0 3.33 usec
P1 10.00 usec
PLW1 39.31399918 W
SFO2 400.3016012 MHz
NUC2 1H
CPDPRG[2 waltz64
PCPD2 80.00 usec
PLW2 8.80000019 W
PLW12 0.20176961 W
PLW13 0.10112690 W

F2 - Processing parameters
SI 131072
SF 100.6555024 MHz
WDW EM
SSB 0
LB 1.00 Hz
GB 0
PC 1.40

3pa

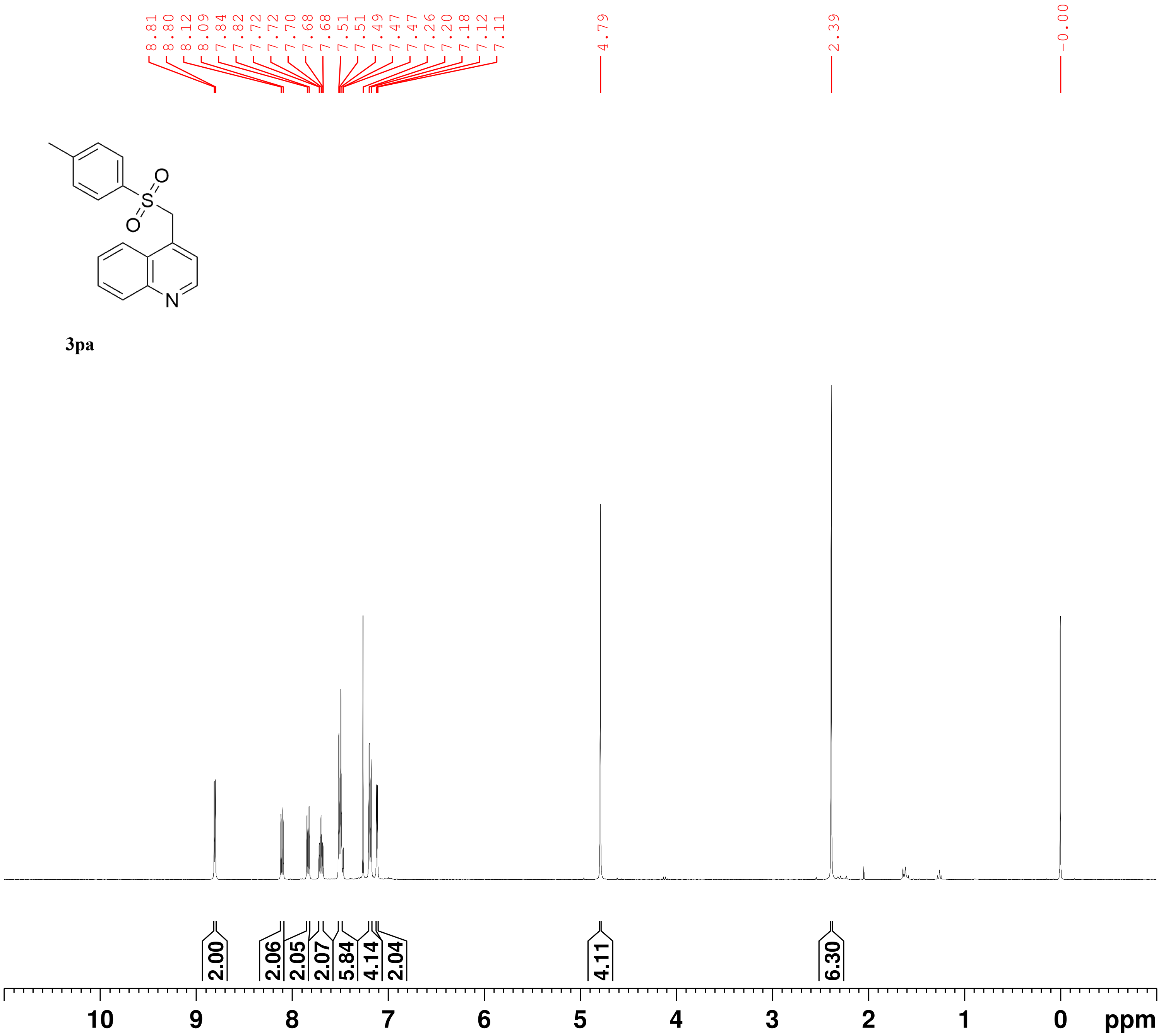


```
Current Data Parameters
NAME        1H ST-07-243
EXPNO                  1
PROCNO                 1

F2 - Acquisition Parameters
Date_           20220601
Time               17.20 h
INSTRUM           Avance
PROBHD   Z167430_0032 (
PULPROG             zg30
TD                 65536
SOLVENT            CDCl3
NS                    16
DS                     0
SWH             8196.722 Hz
FIDRES          0.250144 Hz
AQ             3.9976959 sec
RG                   101
DW                61.000 usec
DE                 13.20 usec
TE                 298.0 K
D1            0.10000000 sec
TD0                    1
SFO1         400.3024719 MHz
NUC1                  1H
P0                  4.00 usec
P1                 12.00 usec
PLW1          8.80000019 W

F2 - Processing parameters
SI                 65536
SF           400.3000089 MHz
WDW                   EM
SSB       0
LB                  0.30 Hz
GB        0
PC                  1.00
```

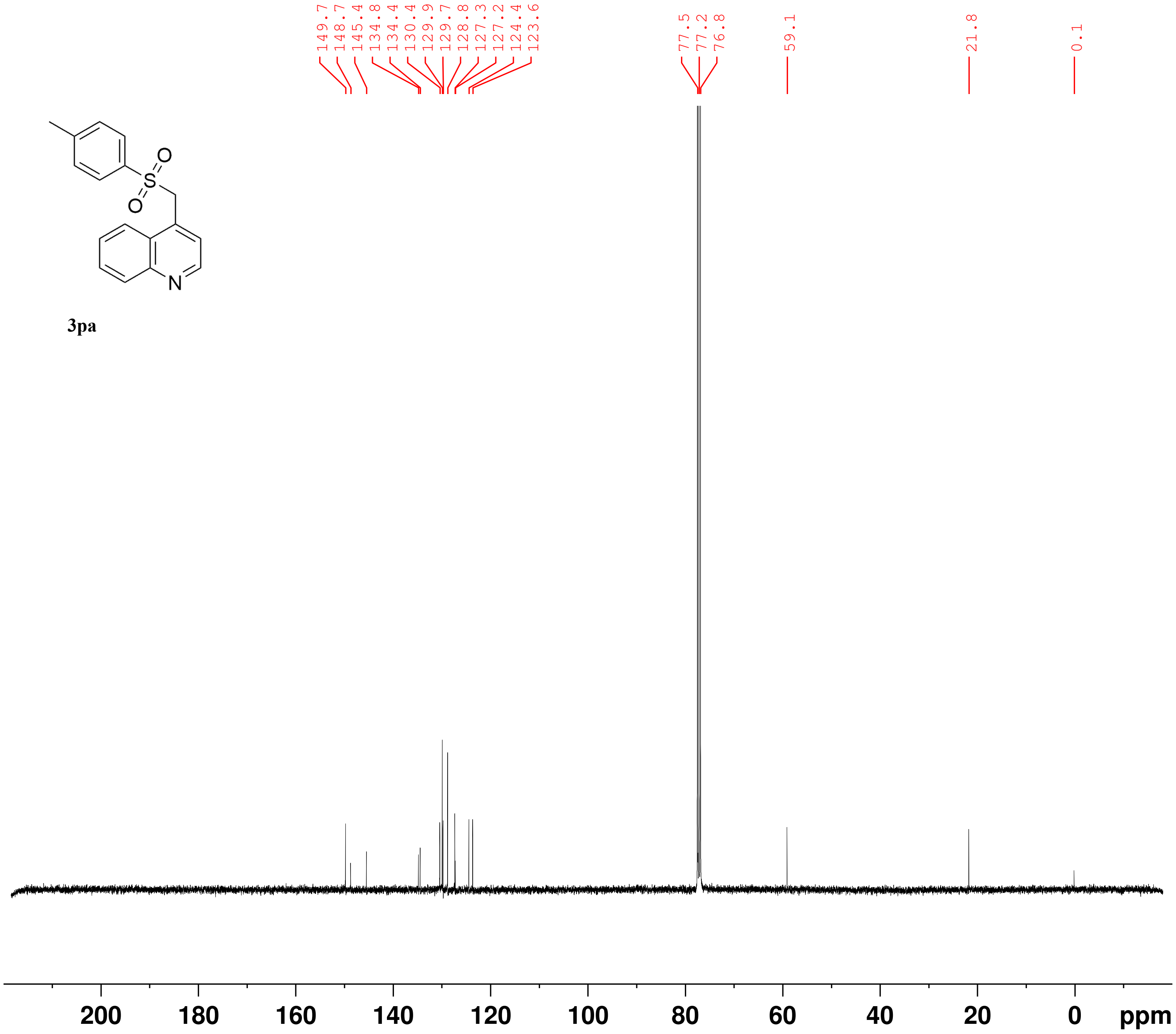

3pa

Current Data Parameters
NAME 13C_ST-7-243
EXPNO 2
PROCNO 1

F2 - Acquisition Parameters
Date_ 20220601
Time 17.32 h
INSTRUM Avance
PROBHD Z167430_0032 (
PULPROG zgpg30
TD 65536
SOLVENT CDCl3
NS 128
DS 4
SWH 23809.523 Hz
FIDRES 0.726609 Hz
AQ 1.3762560 sec
RG 3.25
DW 21.000 usec
DE 19.29 usec
TE 298.0 K
D1 3.00000000 sec
D11 0.03000000 sec
TD0 1
SFO1 100.6655806 MHz
NUC1 13C
P0 3.33 usec
P1 10.00 usec
PLW1 39.31399918 W
SFO2 400.3016012 MHz
NUC2 1H
CPDPRG[2 waltz64
PCPD2 80.00 usec
PLW2 8.80000019 W
PLW12 0.20176961 W
PLW13 0.10112690 W

F2 - Processing parameters
SI 131072
SF 100.6555023 MHz
WDW EM
SSB 0
LB 1.00 Hz
GB 0
PC 1.40

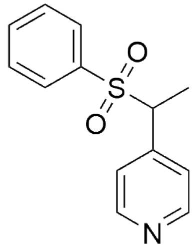

3bb

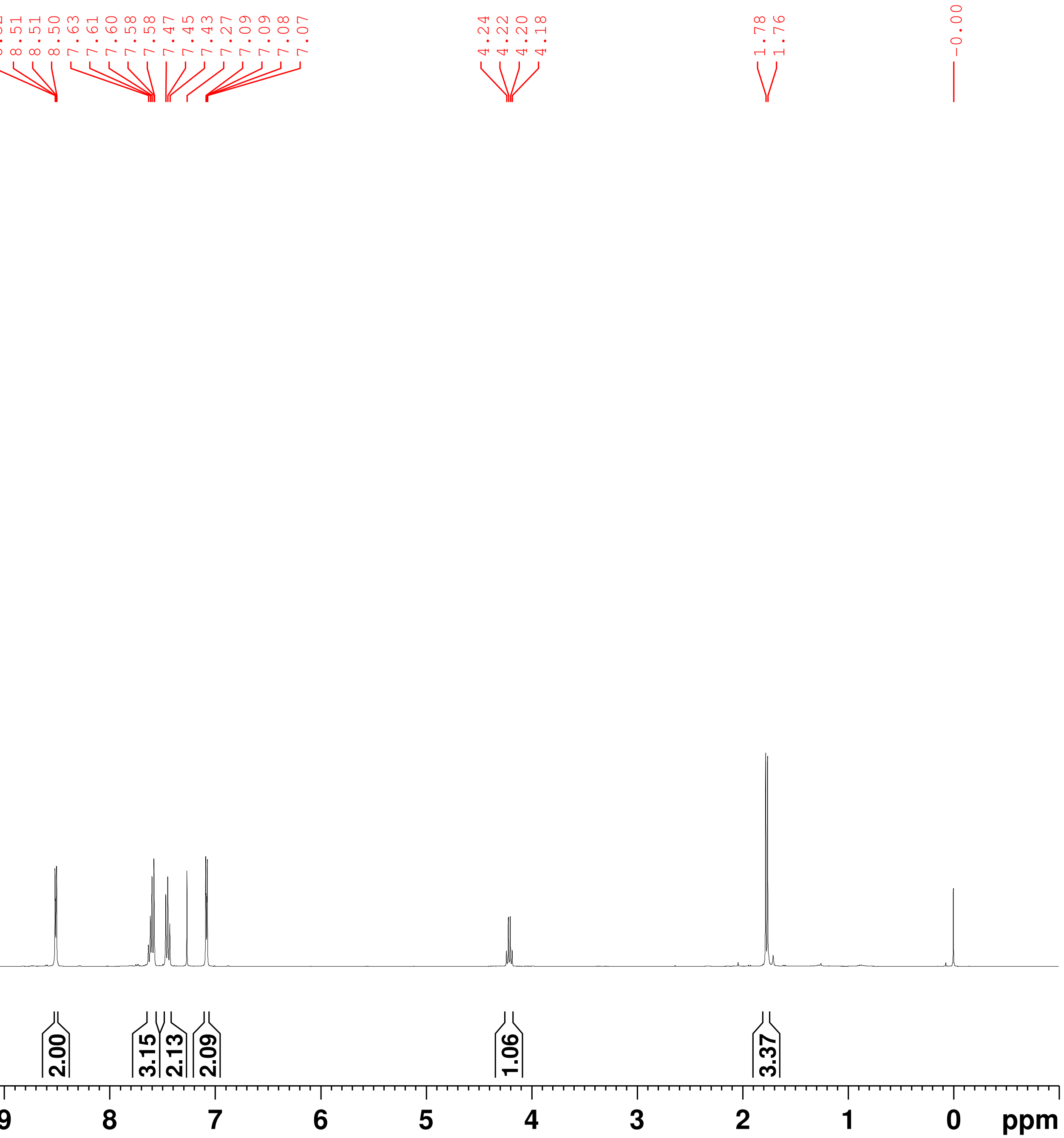


```
Current Data Parameters
NAME         1H ST-07-253
EXPNO                   5
PROCNO                  1

F2 - Acquisition Parameters
Date_            20220612
Time                16.23 h
INSTRUM            Avance
PROBHD     Z167430_0032 (
PULPROG              zg30
TD                  65536
SOLVENT             CDCl3
NS                     16
DS                      0
SWH              8196.722 Hz
FIDRES           0.250144 Hz
AQ              3.9976959 sec
RG                    101
DW                 61.000 usec
DE                  13.20 usec
TE                  298.0 K
D1             0.10000000 sec
TD0                     1
SFO1          400.3024719 MHz
NUC1                   1H
P0                   4.00 usec
P1                  12.00 usec
PLW1           8.80000019 W

F2 - Processing parameters
SI                  65536
SF            400.3000069 MHz
WDW                    EM
SSB      0
LB                   0.30 Hz
GB       0
PC                   1.00
```

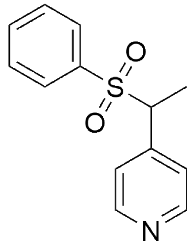

3bb

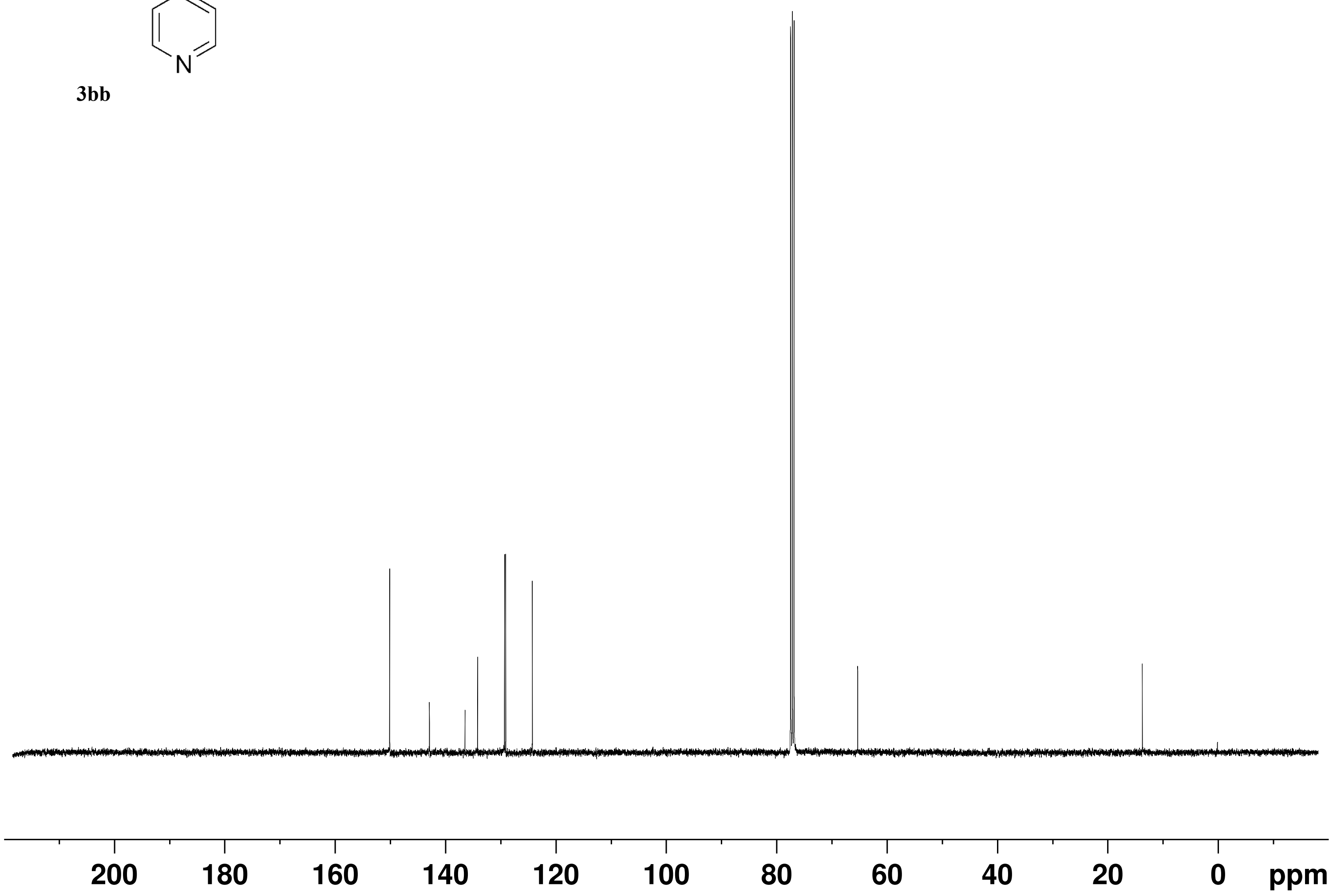

Current Data Parameters
NAME 13C_ST-7-253
EXPNO 1
PROCNO 1

F2 - Acquisition Parameters
Date_ 20220612
Time 16.29 h
INSTRUM Avance
PROBHD Z167430_0032 (
PULPROG zgpg30
TD 65536
SOLVENT CDCl3
NS 64
DS 4
SWH 23809.523 Hz
FIDRES 0.726609 Hz
AQ 1.3762560 sec
RG 3.25
DW 21.000 usec
DE 19.29 usec
TE 298.0 K
D1 3.00000000 sec
D11 0.03000000 sec
TD0 1
SFO1 100.6655806 MHz
NUC1 13C
P0 3.33 usec
P1 10.00 usec
PLW1 39.31399918 W
SFO2 400.3016012 MHz
NUC2 1H
CPDPRG[2 waltz64
PCPD2 80.00 usec
PLW2 8.80000019 W
PLW12 0.20176961 W
PLW13 0.10112690 W

F2 - Processing parameters
SI 131072
SF 100.6555032 MHz
WDW EM
SSB 0
LB 1.00 Hz
GB 0
PC 1.40

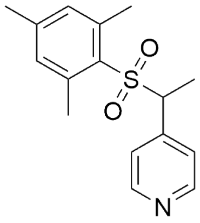

3bc

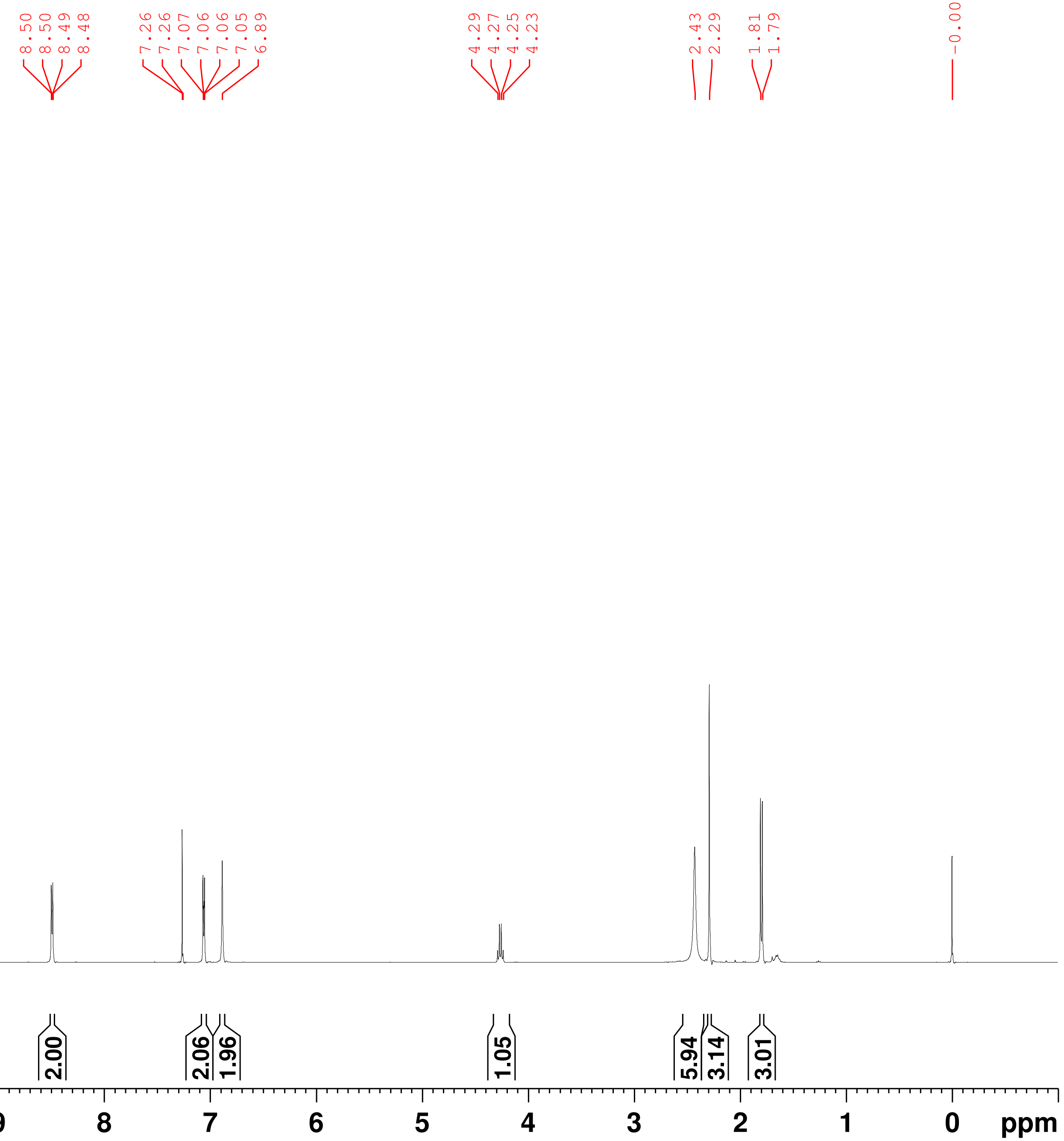

BRUKER

```
Current Data Parameters
NAME              1H_ST-8-9
EXPNO                     3
PROCNO                    1

F2 - Acquisition Parameters
Date_              20220824
Time                 9.00 h
INSTRUM              Avance
PROBHD     Z167430_0032 (
PULPROG                zg30
TD                    65536
SOLVENT               CDCl3
NS                       16
DS                        0
SWH                8196.722 Hz
FIDRES             0.250144 Hz
AQ                3.9976959 sec
RG                      101
DW                   61.000 usec
DE                    13.20 usec
TE                    298.0 K
D1               0.10000000 sec
TD0                       1
SFO1            400.3024719 MHz
NUC1                     1H
P0                     4.00 usec
P1                    12.00 usec
PLW1             8.80000019 W

F2 - Processing parameters
SI                    65536
SF              400.3000083 MHz
WDW                      EM
SSB      0
LB                     0.30 Hz
GB       0
PC                     1.00
```

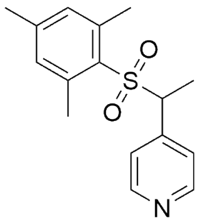

3bc

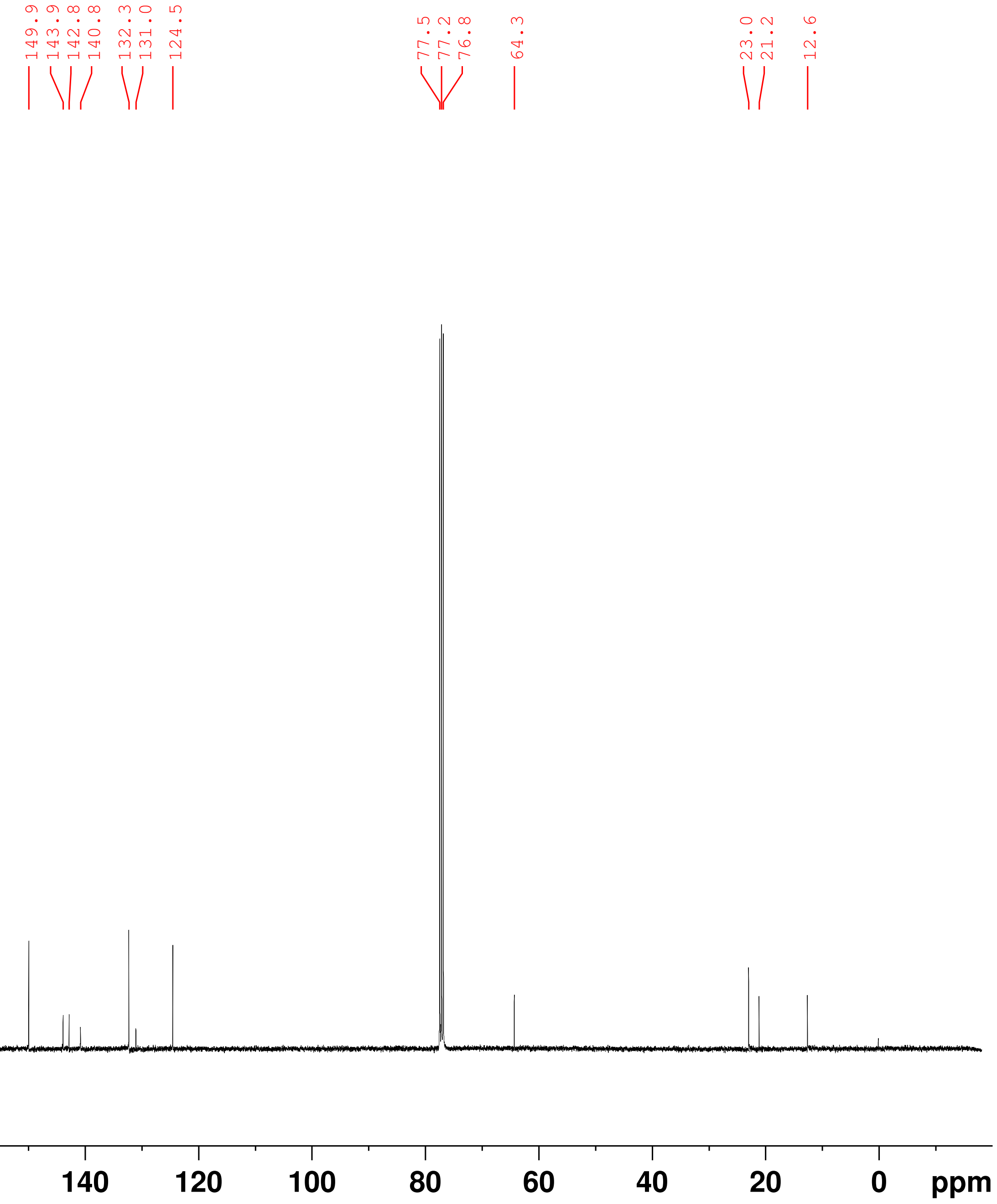

BRUKER

Current Data Parameters
NAME 13C_ST-8-9
EXPNO 1
PROCNO 1

F2 - Acquisition Parameters
Date_ 20220824
Time 9.17 h
INSTRUM Avance
PROBHD Z167430_0032 (
PULPROG zgpg30
TD 65536
SOLVENT CDCl3
NS 128
DS 4
SWH 23809.523 Hz
FIDRES 0.726609 Hz
AQ 1.3762560 sec
RG 3.25
DW 21.000 usec
DE 19.29 usec
TE 298.0 K
D1 3.00000000 sec
D11 0.03000000 sec
TD0 1
SFO1 100.6655806 MHz
NUC1 13C
P0 3.33 usec
P1 10.00 usec
PLW1 39.31399918 W
SFO2 400.3016012 MHz
NUC2 1H
CPDPRG[2 waltz64
PCPD2 80.00 usec
PLW2 8.80000019 W
PLW12 0.20176961 W
PLW13 0.10112690 W

F2 - Processing parameters
SI 131072
SF 100.6555026 MHz
WDW EM
SSB 0
LB 1.00 Hz
GB 0
PC 1.40

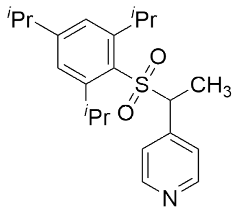

3bd

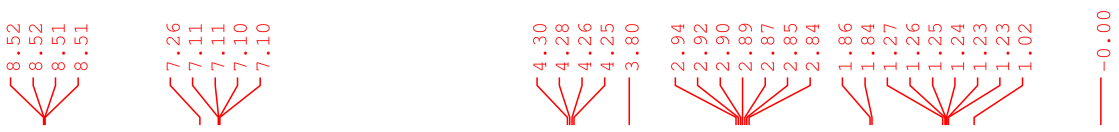

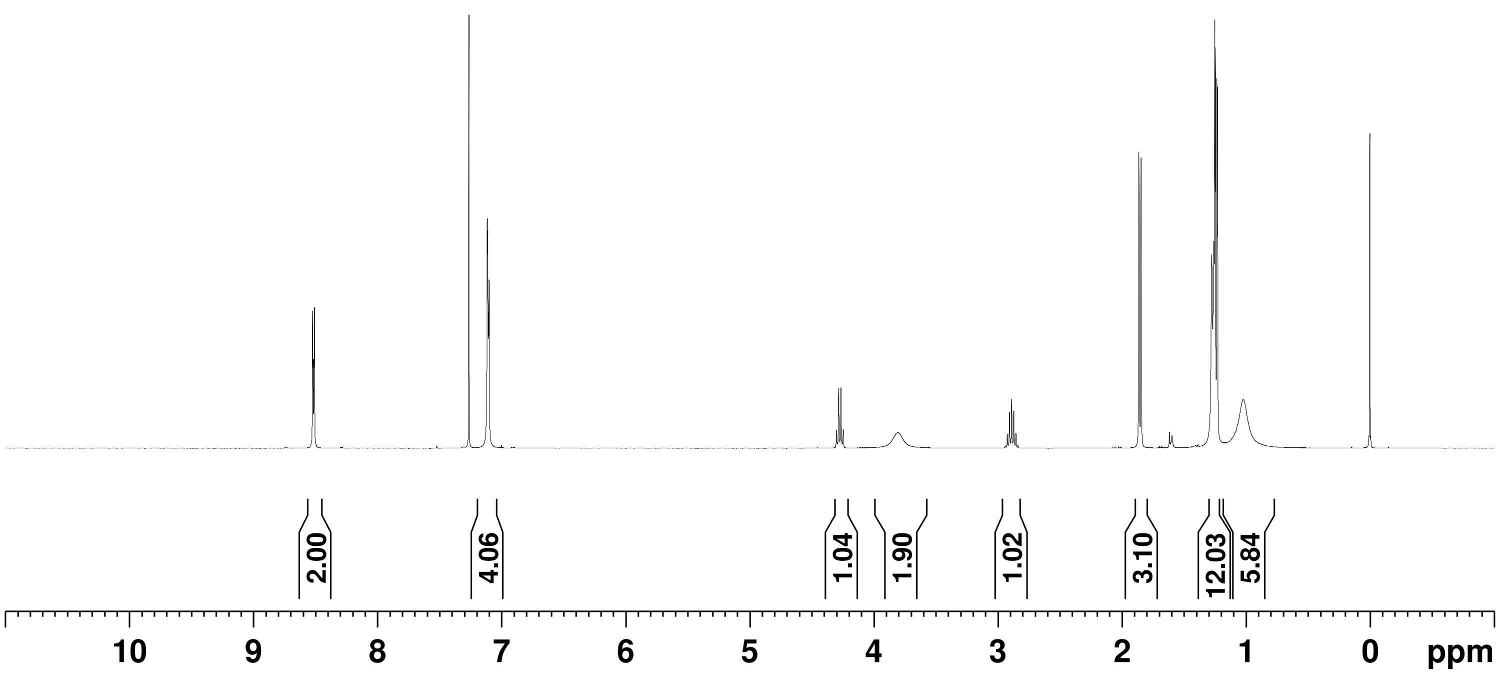

Current Data Parameters
NAME 1H_ST-07-269-re
EXPNO 4
PROCNO 1

F2 - Acquisition Parameters
Date_ 20220704
Time 11.12 h
INSTRUM Avance
PROBHD Z167430_0032 (
PULPROG zg30
TD 65536
SOLVENT CDCl3
NS 16
DS 0
SWH 8196.722 Hz
FIDRES 0.250144 Hz
AQ 3.9976959 sec
RG 101
DW 61.000 usec
DE 13.20 usec
TE 298.0 K
D1 0.10000000 sec
TD0 1
SFO1 400.3024719 MHz
NUC1 1H
P0 4.00 usec
P1 12.00 usec
PLW1 8.80000019 W

F2 - Processing parameters
SI 65536
SF 400.3000088 MHz
WDW EM
SSB 0
LB 0.30 Hz
GB 0
PC 1.00

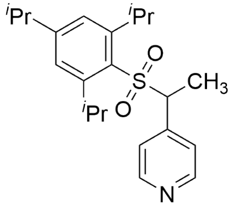

3bd

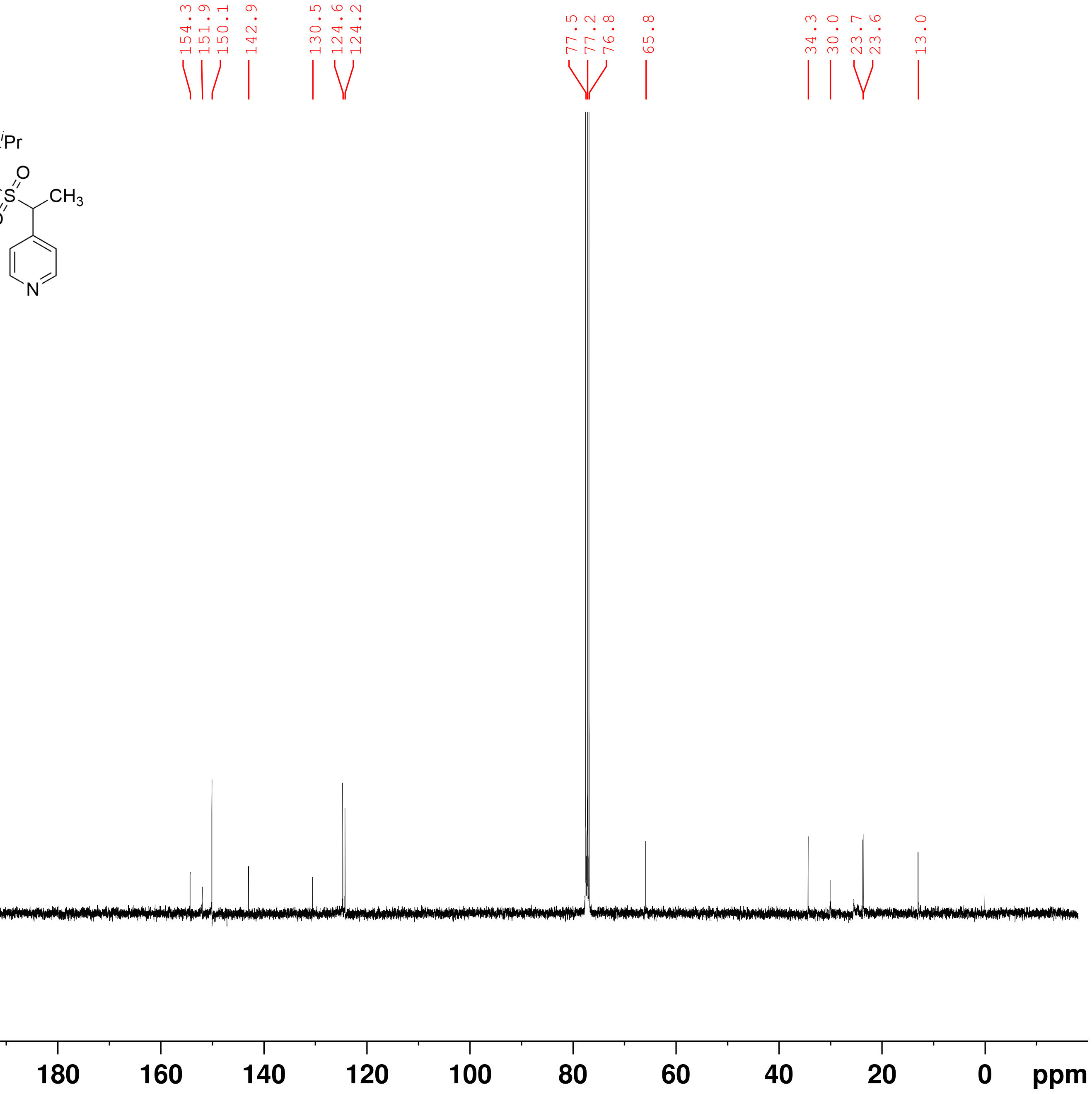

Current Data Parameters
NAME 13C_ST-7-269-re
EXPNO 4
PROCNO 1

F2 - Acquisition Parameters
Date_ 20220704
Time 11.25 h
INSTRUM Avance
PROBHD Z167430_0032 (
PULPROG zgpg30
TD 65536
SOLVENT CDCl3
NS 128
DS 4
SWH 23809.523 Hz
FIDRES 0.726609 Hz
AQ 1.3762560 sec
RG 3.25
DW 21.000 usec
DE 19.29 usec
TE 298.0 K
D1 3.00000000 sec
D11 0.03000000 sec
TD0 1
SFO1 100.6655806 MHz
NUC1 13C
P0 3.33 usec
P1 10.00 usec
PLW1 39.31399918 W
SFO2 400.3016012 MHz
NUC2 1H
CPDPRG[2 waltz64
PCPD2 80.00 usec
PLW2 8.80000019 W
PLW12 0.20176961 W
PLW13 0.10112690 W

F2 - Processing parameters
SI 131072
SF 100.6555019 MHz
WDW EM
SSB 0
LB 1.00 Hz
GB 0
PC 1.40

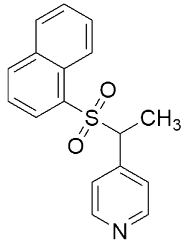

3be

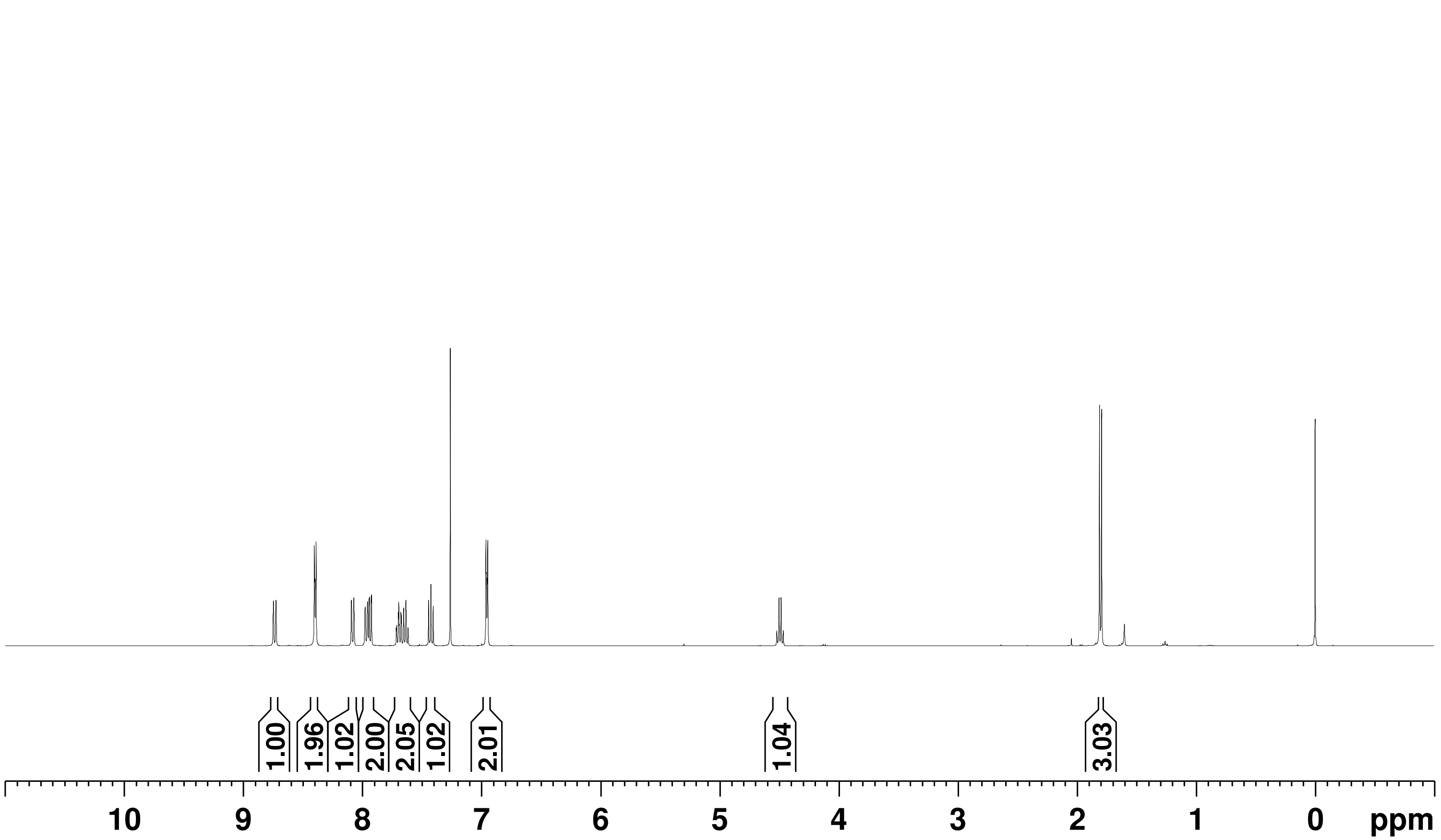


```
Current Data Parameters
NAME       1H_ST-07-271-re
EXPNO                    2
PROCNO                   1

F2 - Acquisition Parameters
Date_             20220704
Time                 11.31 h
INSTRUM             Avance
PROBHD      Z167430_0032 (
PULPROG               zg30
TD                   65536
SOLVENT              CDCl3
NS                      16
DS                       0
SWH               8196.722 Hz
FIDRES            0.250144 Hz
AQ               3.9976959 sec
RG                     101
DW                  61.000 usec
DE                   13.20 usec
TE                   298.0 K
D1              0.10000000 sec
TD0                      1
SFO1           400.3024719 MHz
NUC1                    1H
P0                    4.00 usec
P1                   12.00 usec
PLW1            8.80000019 W

F2 - Processing parameters
SI                   65536
SF             400.3000090 MHz
WDW                     EM
SSB      0
LB                    0.30 Hz
GB       0
PC                    1.00
```

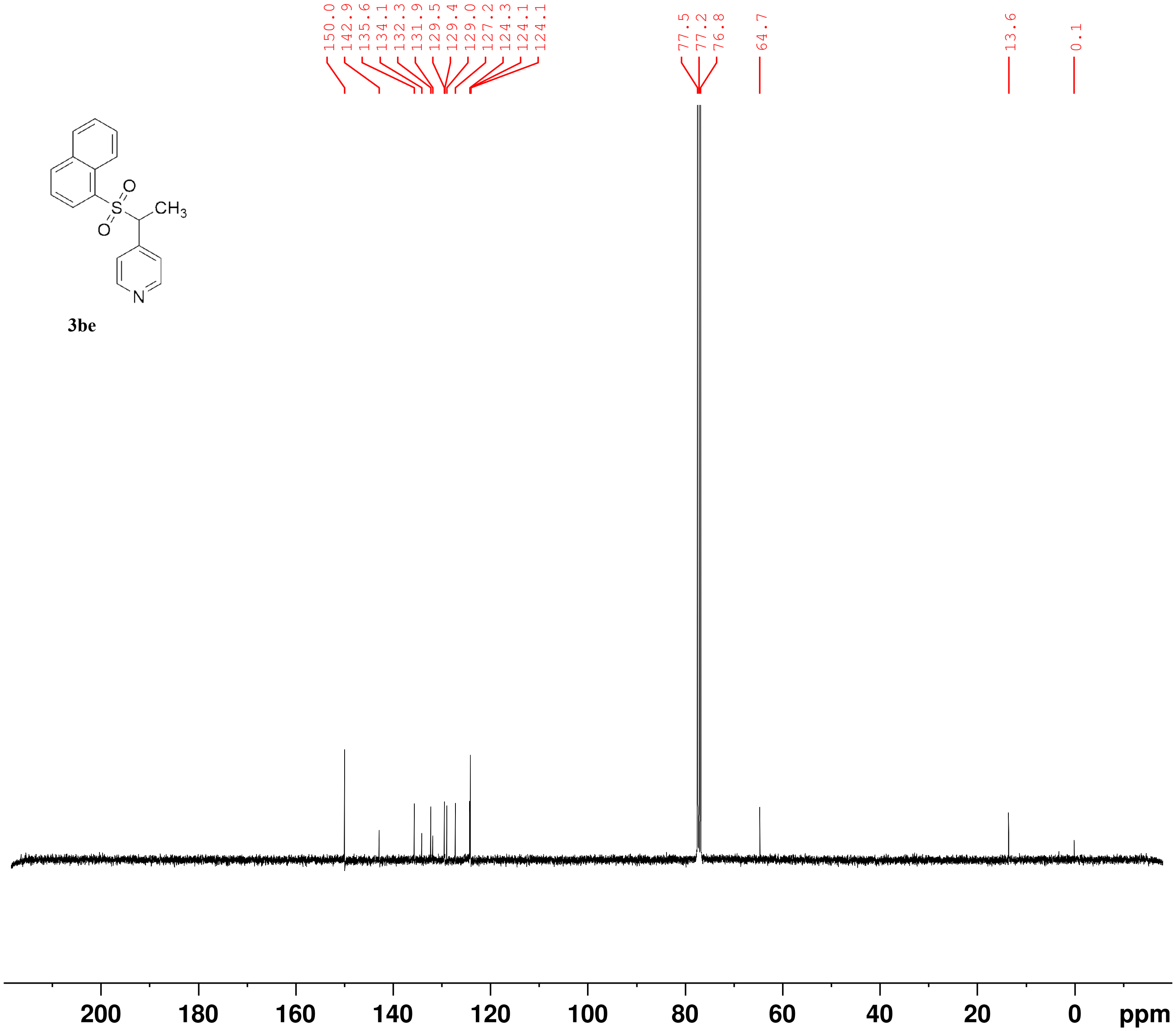

3be

BRUKER

Current Data Parameters
NAME 13C_ST-7-271
EXPNO 4
PROCNO 1

F2 - Acquisition Parameters
Date_ 20220620
Time 18.49 h
INSTRUM Avance
PROBHD Z167430_0032 (
PULPROG zgpg30
TD 65536
SOLVENT CDCl3
NS 128
DS 4
SWH 23809.523 Hz
FIDRES 0.726609 Hz
AQ 1.3762560 sec
RG 3.25
DW 21.000 usec
DE 19.29 usec
TE 298.0 K
D1 3.00000000 sec
D11 0.03000000 sec
TD0 1
SFO1 100.6655806 MHz
NUC1 13C
P0 3.33 usec
P1 10.00 usec
PLW1 39.31399918 W
SFO2 400.3016012 MHz
NUC2 1H
CPDPRG[2 waltz64
PCPD2 80.00 usec
PLW2 8.80000019 W
PLW12 0.20176961 W
PLW13 0.10112690 W

F2 - Processing parameters
SI 131072
SF 100.6555019 MHz
WDW EM
SSB 0
LB 1.00 Hz
GB 0
PC 1.40

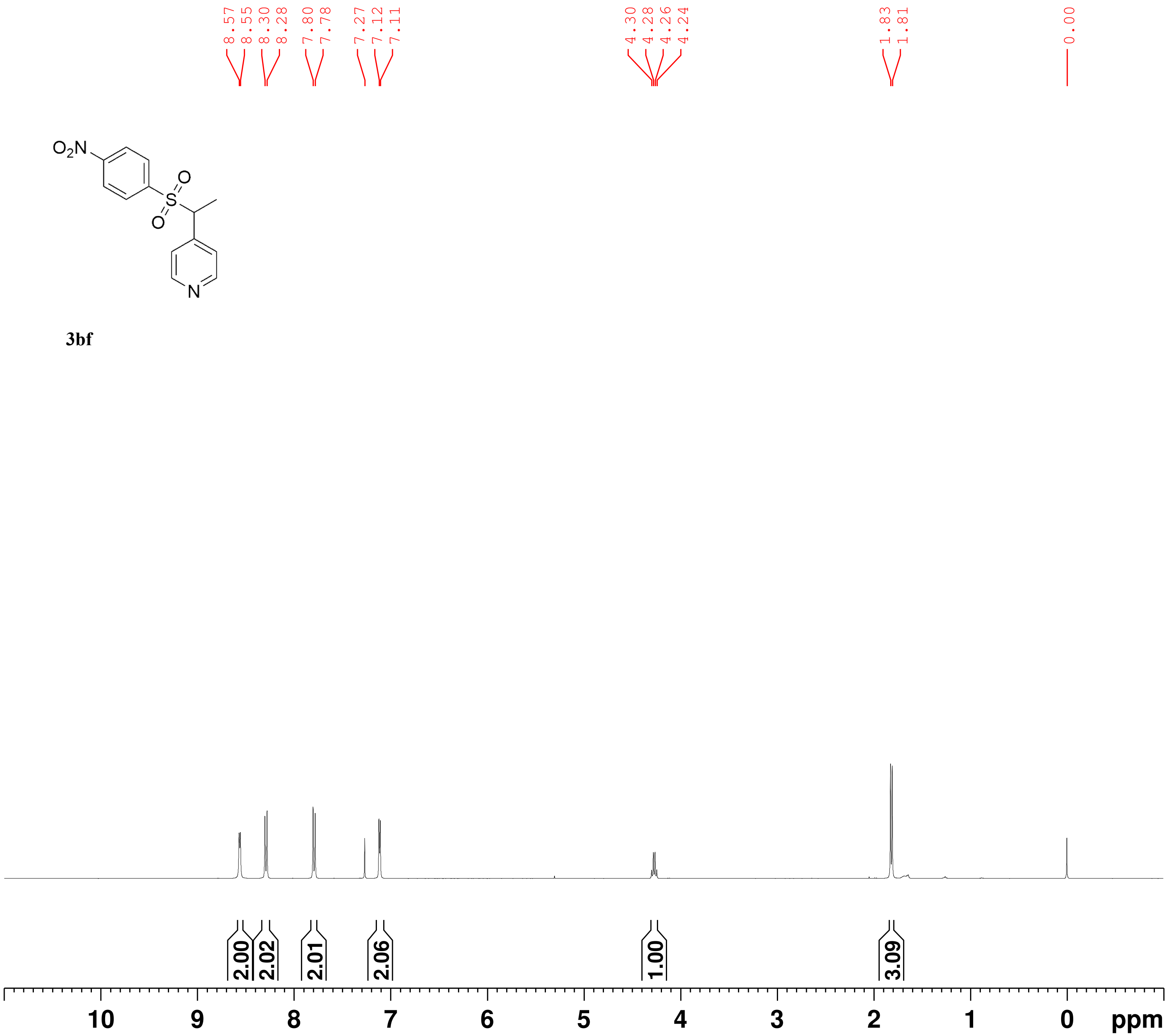

3bf

Current Data Parameters
NAME 1H_ST-8-7
EXPNO 4
PROCNO 1

F2 - Acquisition Parameters
Date_ 20220824
Time 9.22 h
INSTRUM Avance
PROBHD Z167430_0032 (
PULPROG zg30
TD 65536
SOLVENT CDCl3
NS 16
DS 0
SWH 8196.722 Hz
FIDRES 0.250144 Hz
AQ 3.9976959 sec
RG 101
DW 61.000 usec
DE 13.20 usec
TE 298.0 K
D1 0.10000000 sec
TD0 1
SFO1 400.3024719 MHz
NUC1 1H
P0 4.00 usec
P1 12.00 usec
PLW1 8.80000019 W

F2 - Processing parameters
SI 65536
SF 400.3000067 MHz
WDW EM
SSB 0
LB 0.30 Hz
GB 0
PC 1.00

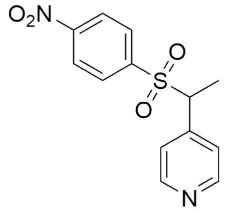

3bf

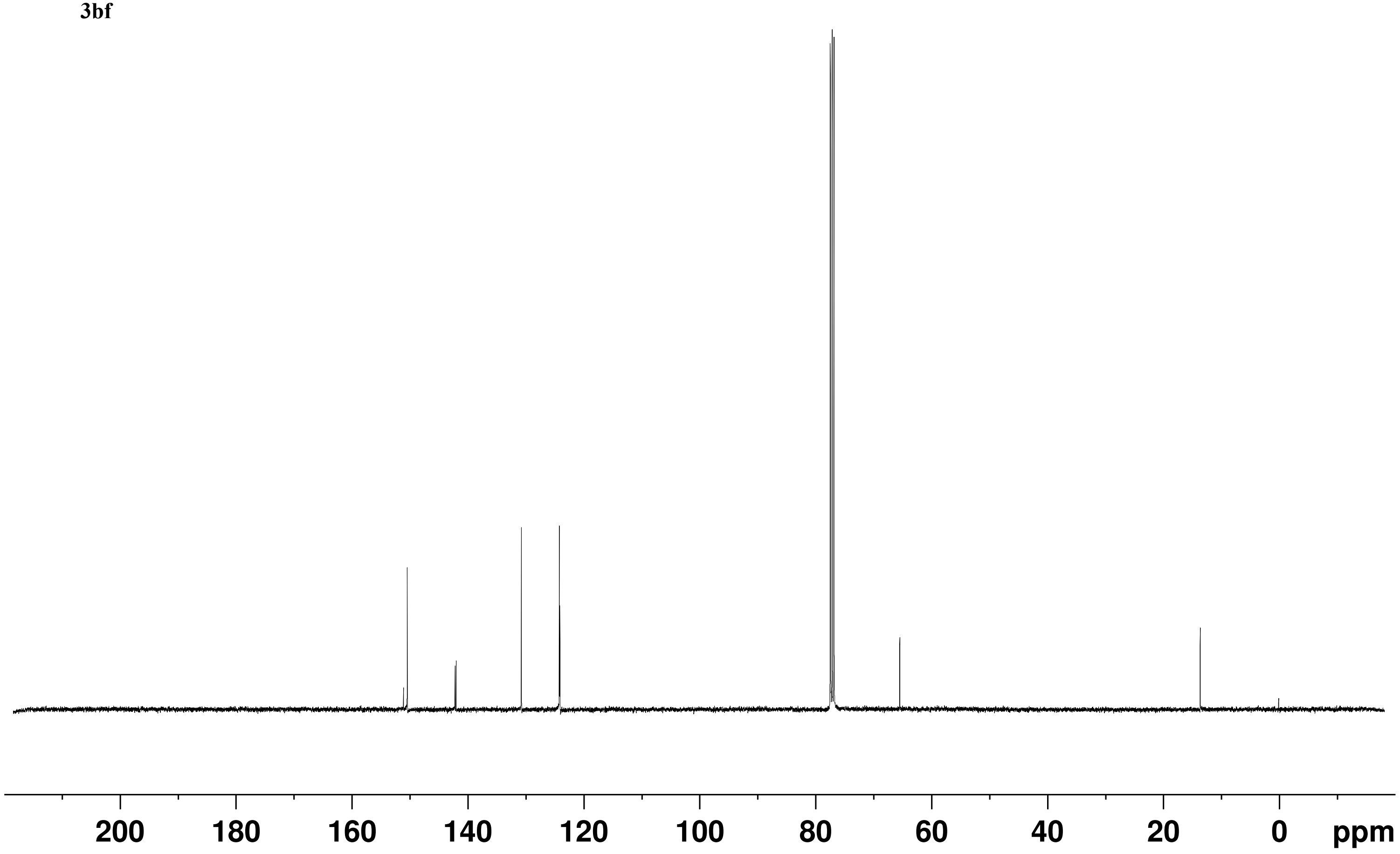

Current Data Parameters
NAME 13C_ST-8-7
EXPNO 2
PROCNO 1

F2 - Acquisition Parameters
Date_ 20220824
Time 9.32 h
INSTRUM Avance
PROBHD Z167430_0032 (
PULPROG zgpg30
TD 65536
SOLVENT CDCl3
NS 128
DS 4
SWH 23809.523 Hz
FIDRES 0.726609 Hz
AQ 1.3762560 sec
RG 3.25
DW 21.000 usec
DE 19.29 usec
TE 298.0 K
D1 3.00000000 sec
D11 0.03000000 sec
TD0 1
SFO1 100.6655806 MHz
NUC1 13C
P0 3.33 usec
P1 10.00 usec
PLW1 39.31399918 W
SFO2 400.3016012 MHz
NUC2 1H
CPDPRG[2 waltz64
PCPD2 80.00 usec
PLW2 8.80000019 W
PLW12 0.20176961 W
PLW13 0.10112690 W

F2 - Processing parameters
SI 131072
SF 100.6555033 MHz
WDW EM
SSB 0
LB 1.00 Hz
GB 0
PC 1.40

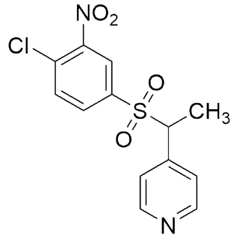

3bg

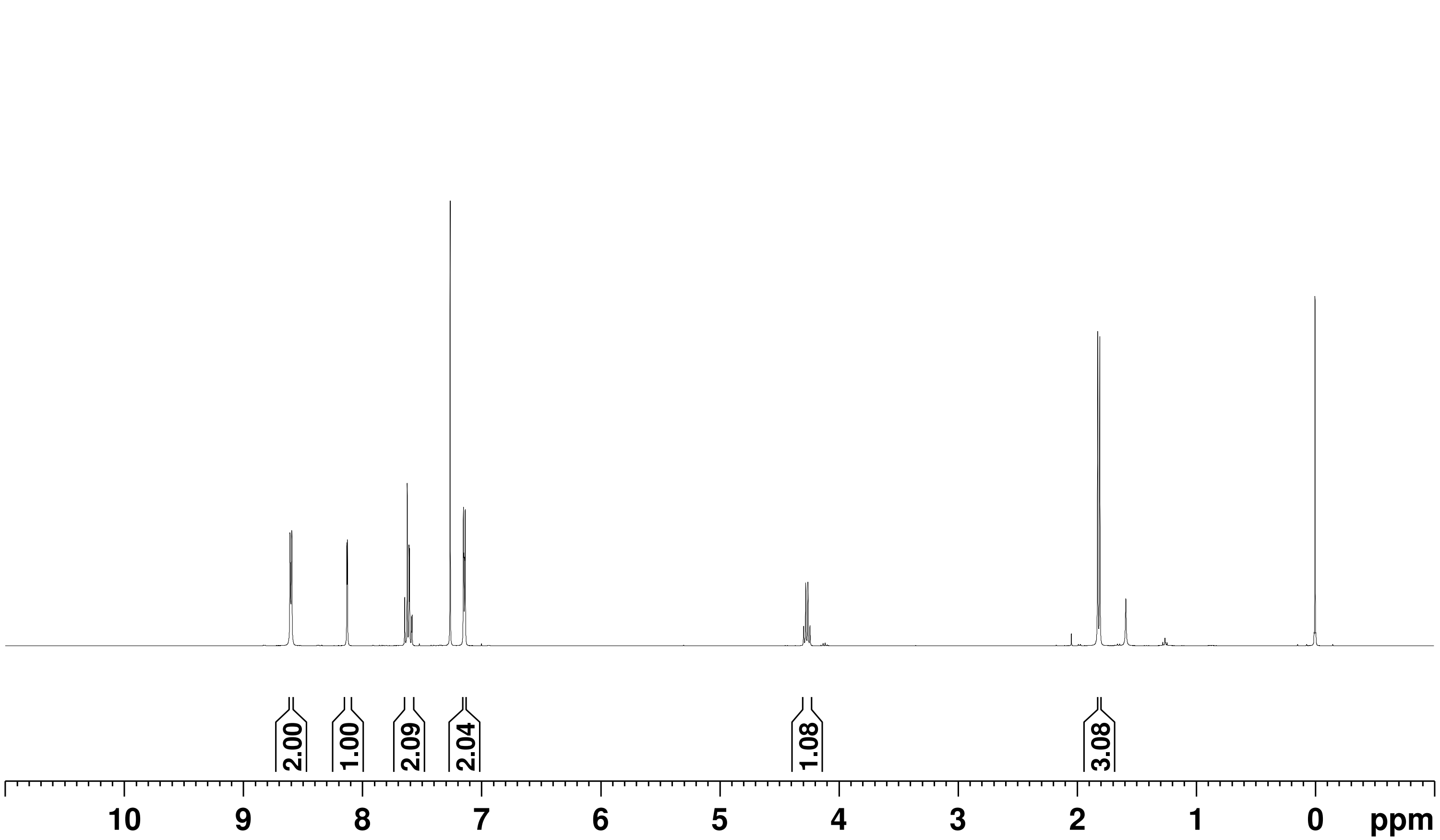

Current Data Parameters
NAME 1H_ST-07-265
EXPNO 2
PROCNO 1

F2 - Acquisition Parameters

Date_	20220620
Time	18.02 h
INSTRUM	Avance
PROBHD	Z167430_0032 (
PULPROG	zg30
TD	65536
SOLVENT	CDCl3
NS	16
DS	0
SWH	8196.722 Hz
FIDRES	0.250144 Hz
AQ	3.9976959 sec
RG	101
DW	61.000 usec
DE	13.20 usec
TE	298.0 K
D1	0.10000000 sec
TD0	1
SFO1	400.3024719 MHz
NUC1	1H
P0	4.00 usec
P1	12.00 usec
PLW1	8.80000019 W

F2 - Processing parameters

SI	65536
SF	400.3000087 MHz
WDW	EM
SSB	0
LB	0.30 Hz
GB	0
PC	1.00

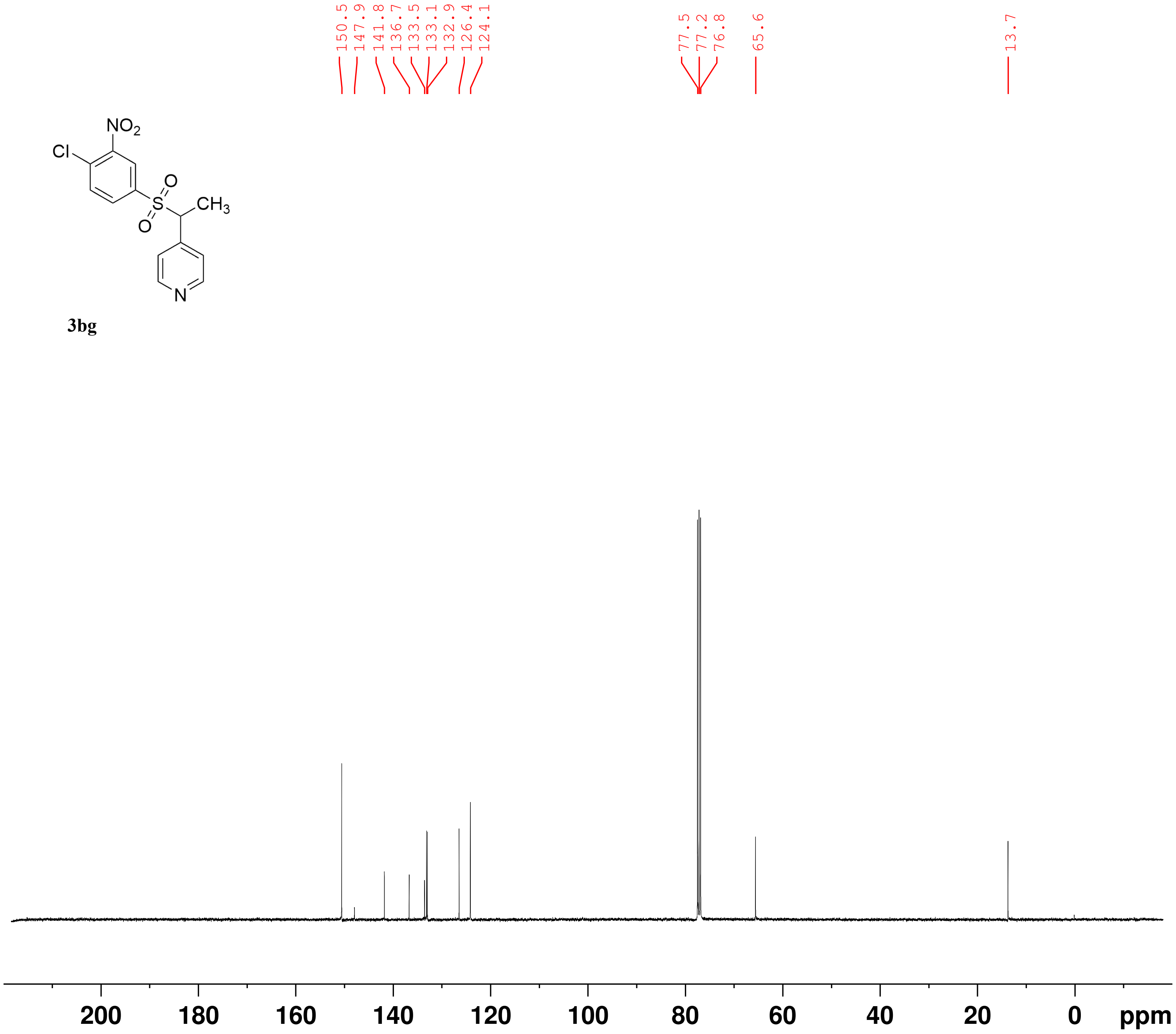

BRUKER

Current Data Parameters
NAME 13C_ST-7-265-re
EXPNO 3
PROCNO 1

F2 - Acquisition Parameters
Date_ 20220704
Time 11.03 h
INSTRUM Avance
PROBHD Z167430_0032 (
PULPROG zgpg30
TD 65536
SOLVENT CDCl3
NS 128
DS 4
SWH 23809.523 Hz
FIDRES 0.726609 Hz
AQ 1.3762560 sec
RG 3.25
DW 21.000 usec
DE 19.29 usec
TE 298.0 K
D1 3.00000000 sec
D11 0.03000000 sec
TD0 1
SFO1 100.6655806 MHz
NUC1 13C
P0 3.33 usec
P1 10.00 usec
PLW1 39.31399918 W
SFO2 400.3016012 MHz
NUC2 1H
CPDPRG[2 waltz64
PCPD2 80.00 usec
PLW2 8.80000019 W
PLW12 0.20176961 W
PLW13 0.10112690 W

F2 - Processing parameters
SI 131072
SF 100.6555045 MHz
WDW EM
SSB 0
LB 1.00 Hz
GB 0
PC 1.40

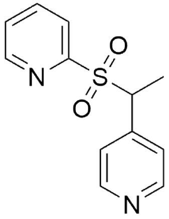

3bh

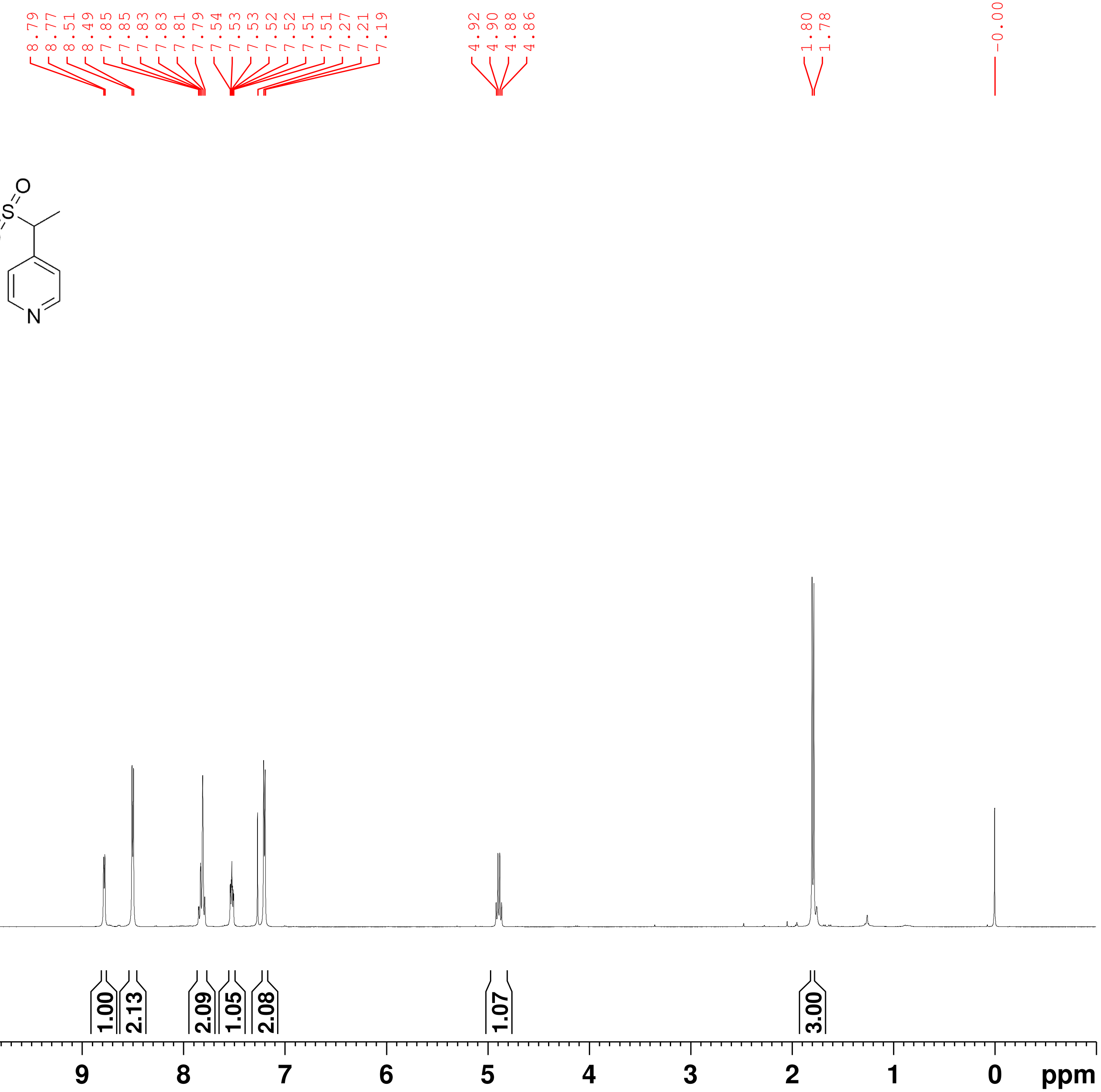


```
Current Data Parameters
NAME       1H_ST-8-49-real
EXPNO                    1
PROCNO                   1

F2 - Acquisition Parameters
Date_             20220907
Time                 16.29 h
INSTRUM             Avance
PROBHD    Z167430_0032 (
PULPROG               zg30
TD                   65536
SOLVENT              CDCl3
NS                      16
DS                       0
SWH               8196.722 Hz
FIDRES            0.250144 Hz
AQ               3.9976959 sec
RG                     101
DW                  61.000 usec
DE                   13.20 usec
TE                   298.0 K
D1              0.10000000 sec
TD0                      1
SFO1           400.3024719 MHz
NUC1                    1H
P0                    4.00 usec
P1                   12.00 usec
PLW1            8.80000019 W

F2 - Processing parameters
SI                   65536
SF             400.3000062 MHz
WDW                     EM
SSB      0
LB                    0.30 Hz
GB       0
PC                    1.00
```

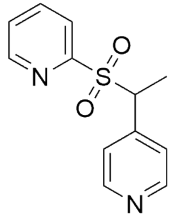

3bh

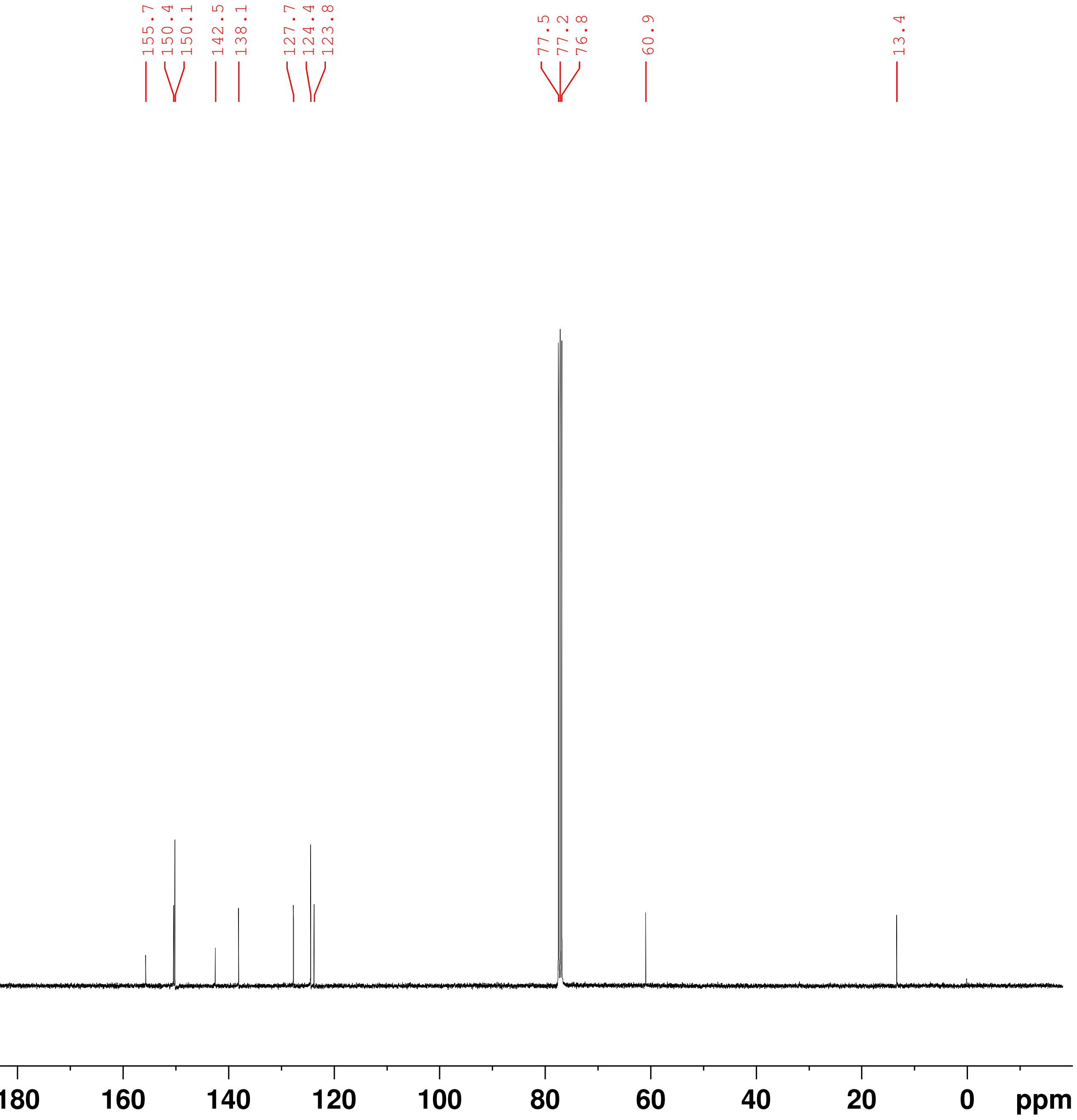

Current Data Parameters
NAME 13C_ST-8-49-real
EXPNO 1
PROCNO 1

F2 - Acquisition Parameters
Date_ 20220907
Time 16.38 h
INSTRUM Avance
PROBHD Z167430_0032 (
PULPROG zgpg30
TD 65536
SOLVENT CDCl3
NS 128
DS 4
SWH 23809.523 Hz
FIDRES 0.726609 Hz
AQ 1.3762560 sec
RG 3.25
DW 21.000 usec
DE 19.29 usec
TE 298.0 K
D1 2.00000000 sec
D11 0.03000000 sec
TD0 1
SFO1 100.6655806 MHz
NUC1 13C
P0 3.33 usec
P1 10.00 usec
PLW1 39.31399918 W
SFO2 400.3016012 MHz
NUC2 1H
CPDPRG[2 waltz64
PCPD2 80.00 usec
PLW2 8.80000019 W
PLW12 0.20176961 W
PLW13 0.10112690 W

F2 - Processing parameters
SI 131072
SF 100.6555035 MHz
WDW EM
SSB 0
LB 1.00 Hz
GB 0
PC 1.40

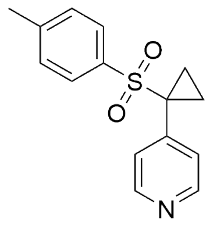

10

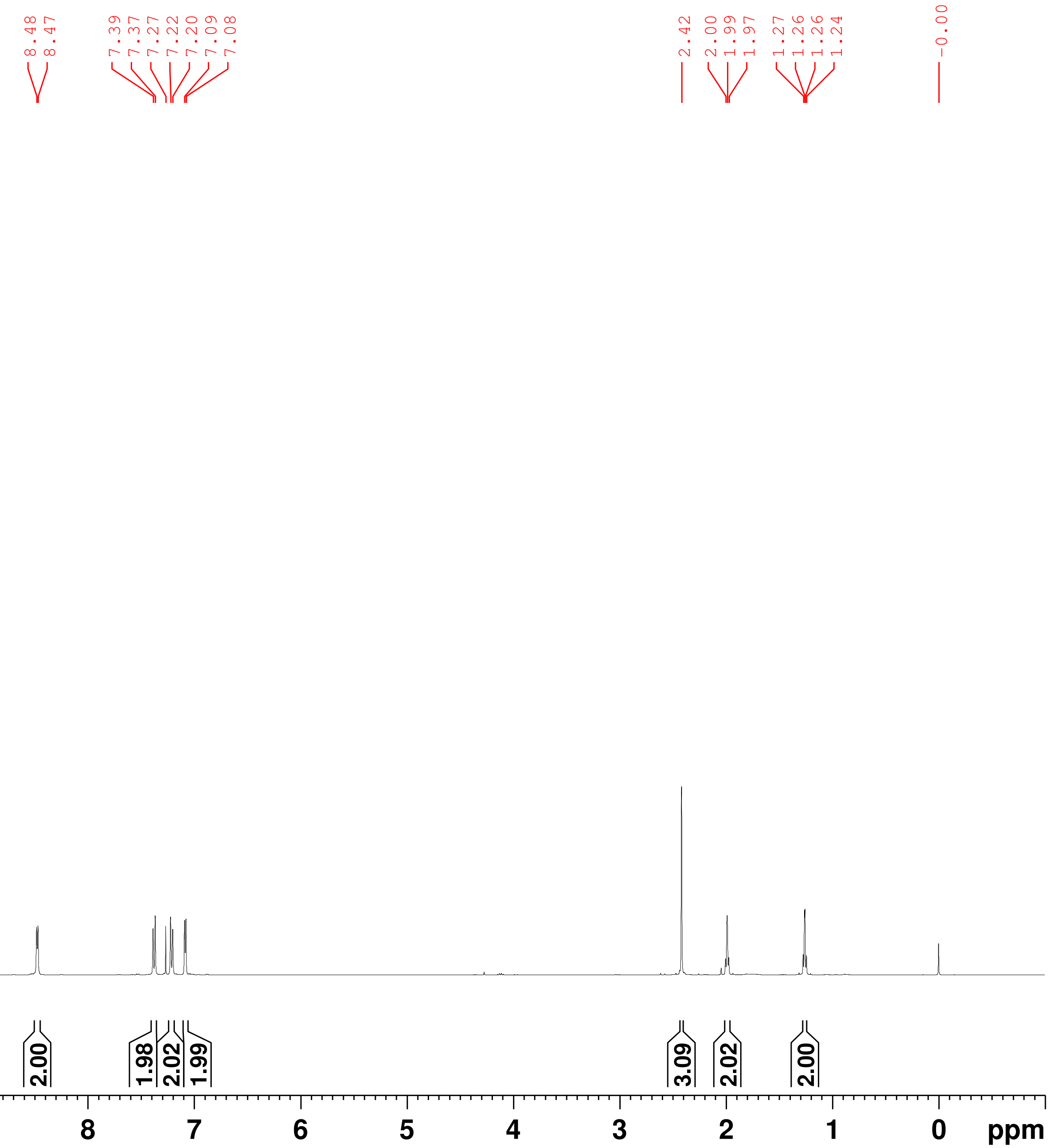

Current Data Parameters
NAME 1H_JH-1-27-again
EXPNO 2
PROCNO 1

F2 - Acquisition Parameters
Date_ 20221026
Time 18.54 h
INSTRUM Avance
PROBHD Z167430_0032 (
PULPROG zg30
TD 65536
SOLVENT CDCl3
NS 7
DS 0
SWH 8196.722 Hz
FIDRES 0.250144 Hz
AQ 3.9976959 sec
RG 101
DW 61.000 usec
DE 13.20 usec
TE 298.0 K
D1 0.10000000 sec
TD0 1
SFO1 400.3024719 MHz
NUC1 1H
P0 4.00 usec
P1 12.00 usec
PLW1 8.80000019 W

F2 - Processing parameters
SI 65536
SF 400.3000066 MHz
WDW EM
SSB 0
LB 0.30 Hz
GB 0
PC 1.00

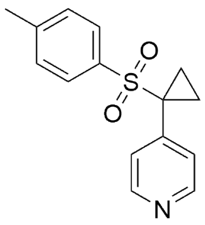

10

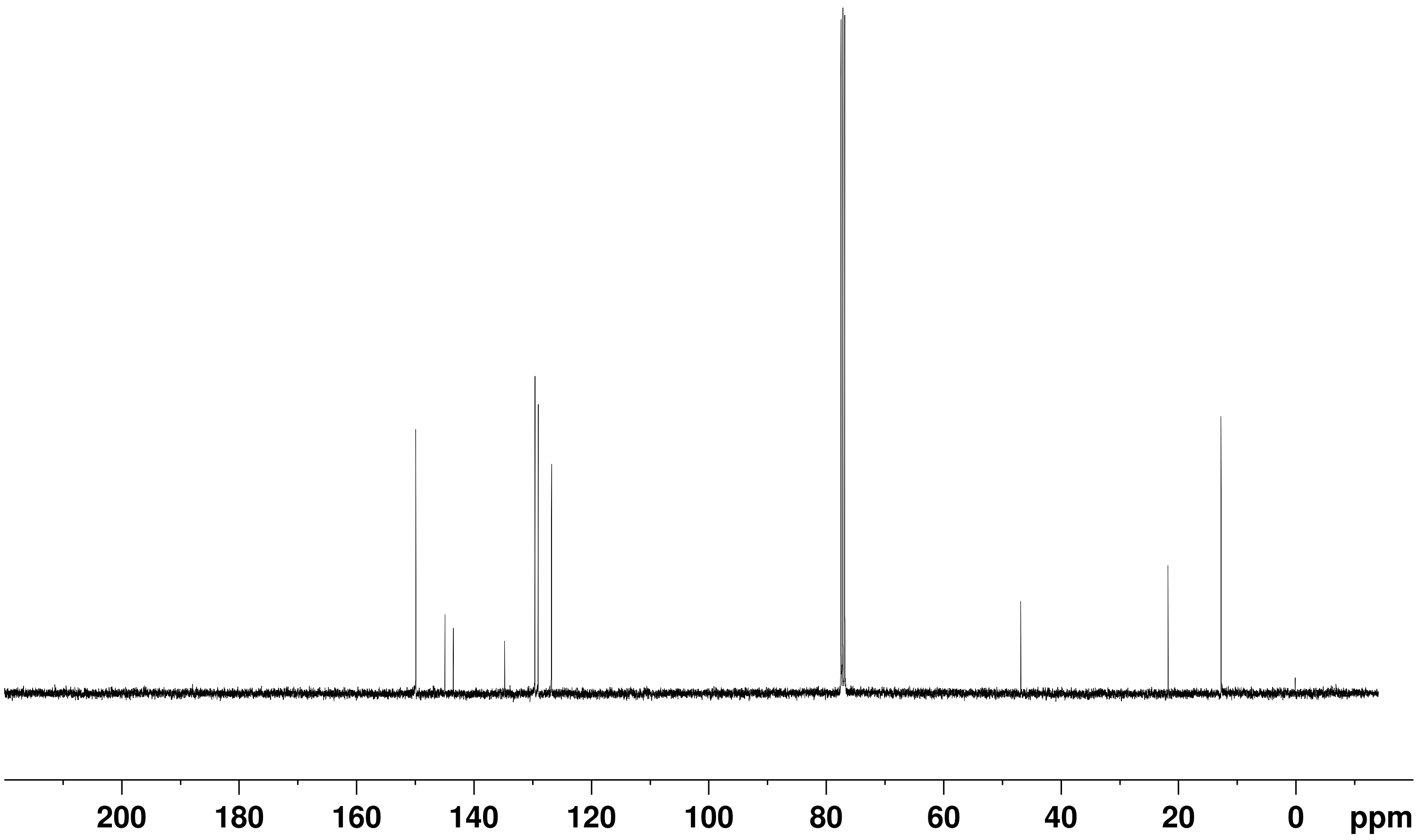

BRUKER

```
Current Data Parameters
NAME       13C_JH-1-27-again
EXPNO                      3
PROCNO                     1

F2 - Acquisition Parameters
Date_              20221026
Time                  18.58 h
INSTRUM              Avance
PROBHD     Z167430_0032 (
PULPROG             zgpg30
TD                   65536
SOLVENT              CDCl3
NS                      64
DS                       4
SWH              25000.000 Hz
FIDRES            0.762939 Hz
AQ               1.3107200 sec
RG                    3.25
DW                  20.000 usec
DE                   18.29 usec
TE                   298.0 K
D1              2.00000000 sec
D11             0.03000000 sec
TD0                      1
SFO1           100.6665872 MHz
NUC1                   13C
P0                    3.33 usec
P1                   10.00 usec
PLW1           39.31399918 W
SFO2           400.3016012 MHz
NUC2                    1H
CPDPRG[2           waltz64
PCPD2                80.00 usec
PLW2            8.80000019 W
PLW12           0.20176961 W
PLW13           0.10112690 W

F2 - Processing parameters
SI                  131072
SF             100.6555035 MHz
WDW                     EM
SSB       0
LB                    1.00 Hz
GB        0
PC                    1.40
```

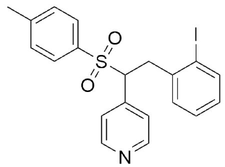

11

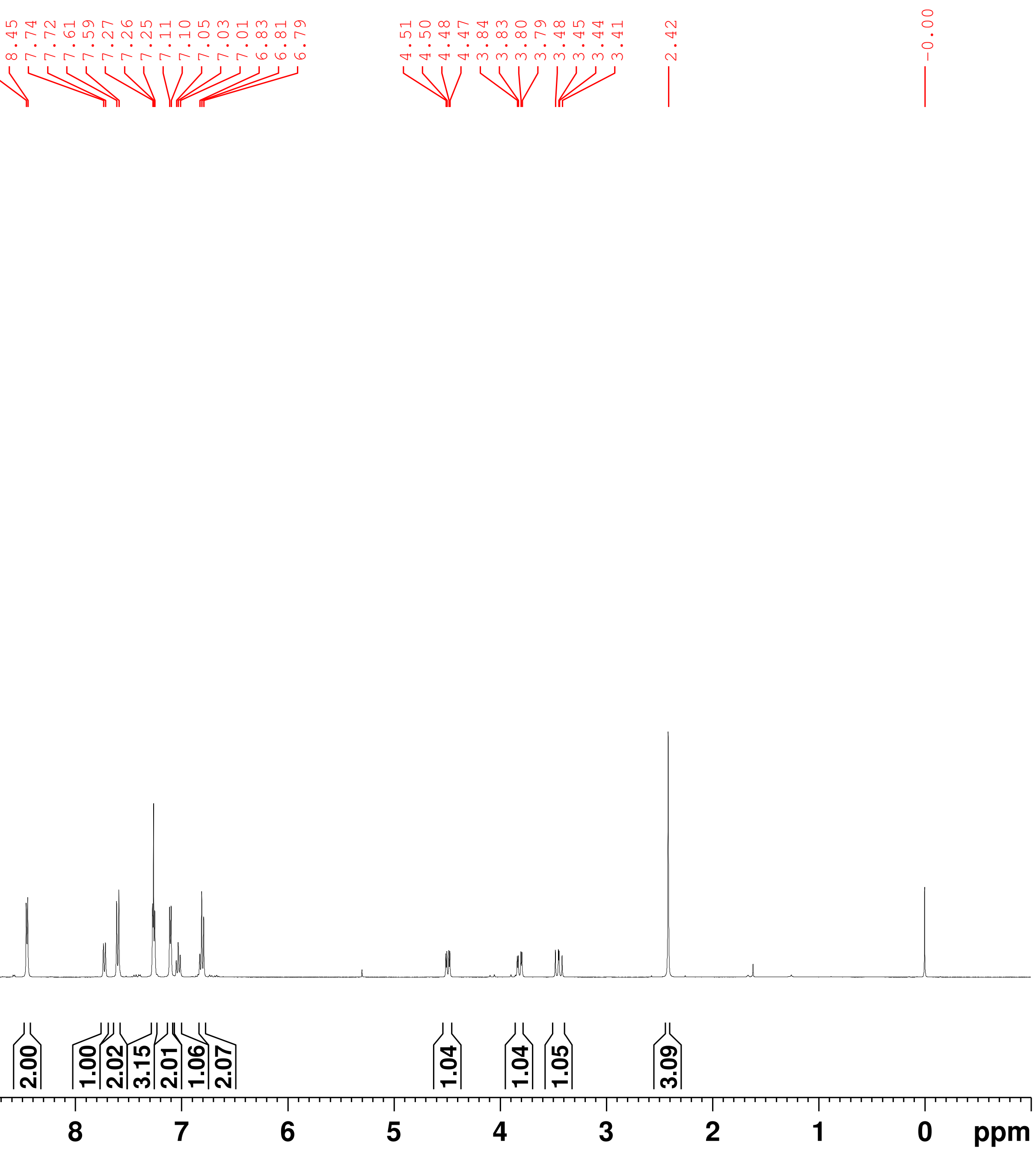

BRUKER

```
Current Data Parameters
NAME          1H_JH-1-65
EXPNO                  2
PROCNO                 1

F2 - Acquisition Parameters
Date_           20220725
Time            10.58 h
INSTRUM           Avance
PROBHD   Z167430_0032 (
PULPROG             zg30
TD                 65536
SOLVENT            CDCl3
NS                    16
DS                     0
SWH             8196.722 Hz
FIDRES          0.250144 Hz
AQ             3.9976959 sec
RG                   101
DW                61.000 usec
DE                 13.20 usec
TE                 298.0 K
D1            0.10000000 sec
TD0                    1
SFO1         400.3024719 MHz
NUC1                  1H
P0                  4.00 usec
P1                 12.00 usec
PLW1          8.80000019 W

F2 - Processing parameters
SI                 65536
SF           400.3000089 MHz
WDW                   EM
SSB      0
LB                  0.30 Hz
GB       0
PC                  1.00
```

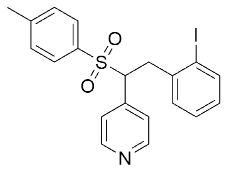

11

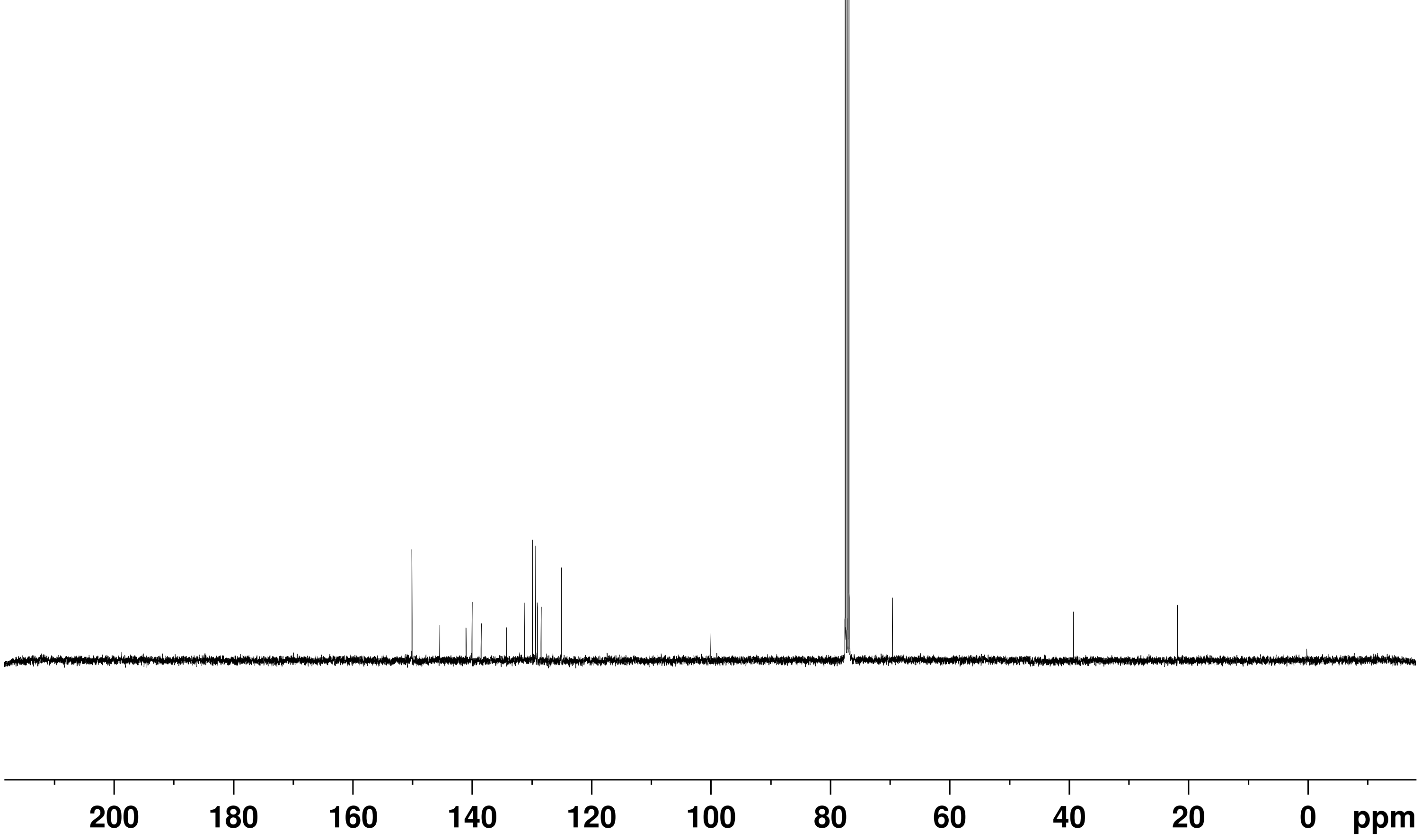

Current Data Parameters
NAME 13C_JH-1-65
EXPNO 1
PROCNO 1

F2 - Acquisition Parameters
Date_ 20220725
Time 11.04 h
INSTRUM Avance
PROBHD Z167430_0032 (
PULPROG zgpg30
TD 65536
SOLVENT CDCl3
NS 64
DS 4
SWH 23809.523 Hz
FIDRES 0.726609 Hz
AQ 1.3762560 sec
RG 3.25
DW 21.000 usec
DE 19.29 usec
TE 298.0 K
D1 2.00000000 sec
D11 0.03000000 sec
TD0 1
SFO1 100.6655806 MHz
NUC1 13C
P0 3.33 usec
P1 10.00 usec
PLW1 39.31399918 W
SFO2 400.3016012 MHz
NUC2 1H
CPDPRG[2 waltz64
PCPD2 80.00 usec
PLW2 8.80000019 W
PLW12 0.20176961 W
PLW13 0.10112690 W

F2 - Processing parameters
SI 131072
SF 100.6555026 MHz
WDW EM
SSB 0
LB 1.00 Hz
GB 0
PC 1.40

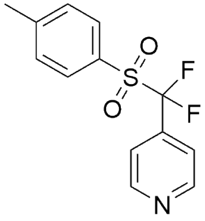

12

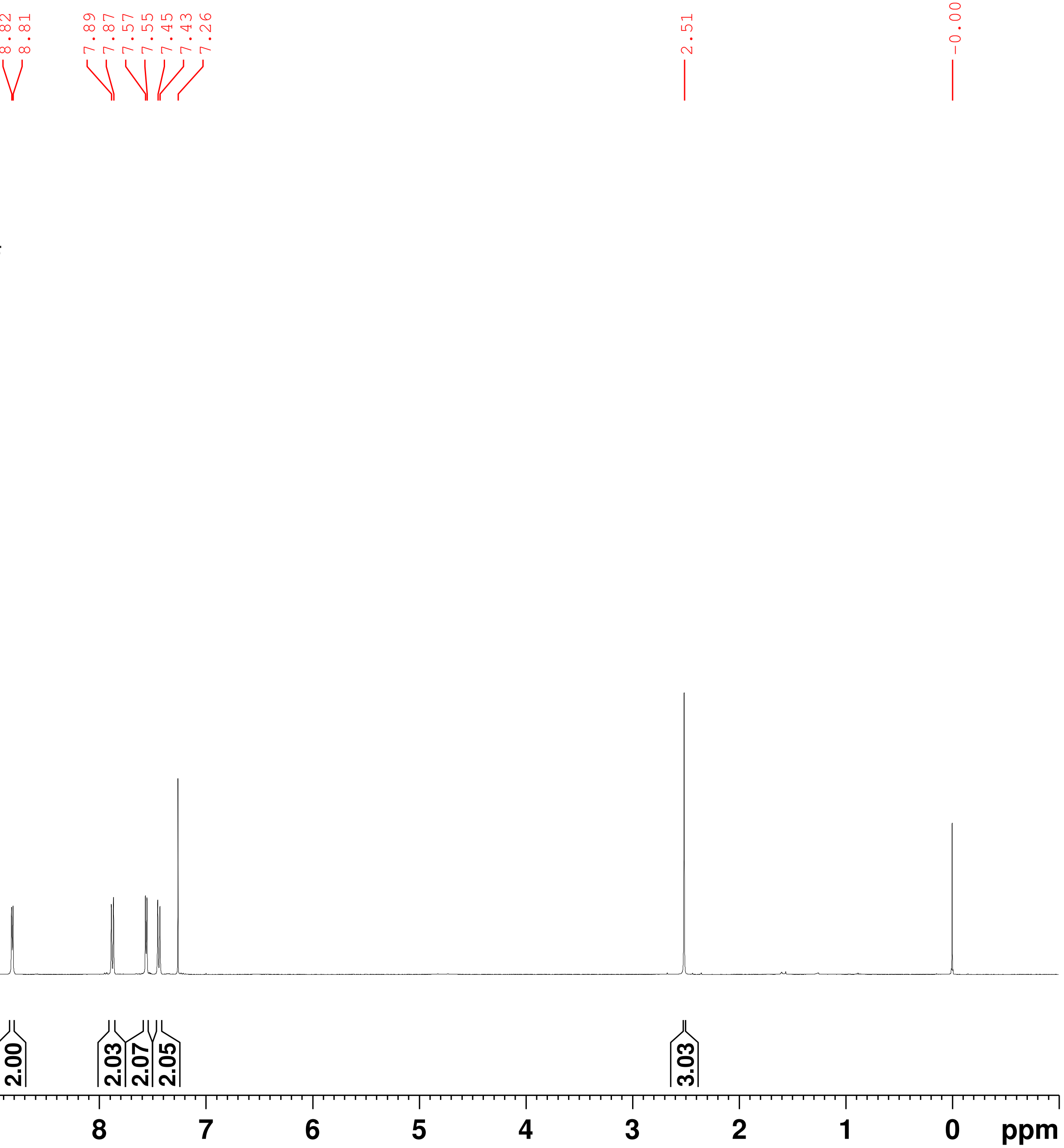


```
Current Data Parameters
NAME              1H_ST-8-43
EXPNO                      1
PROCNO                     1

F2 - Acquisition Parameters
Date_               20220926
Time                   13.46 h
INSTRUM               Avance
PROBHD     Z167430_0032 (
PULPROG                 zg30
TD                     65536
SOLVENT                CDCl3
NS                        16
DS                         2
SWH                 8196.722 Hz
FIDRES              0.250144 Hz
AQ                 3.9976959 sec
RG                       101
DW                    61.000 usec
DE                     13.20 usec
TE                     298.0 K
D1                1.00000000 sec
TD0                        1
SFO1             400.3024719 MHz
NUC1                      1H
P0                      4.00 usec
P1                     12.00 usec
PLW1              8.80000019 W

F2 - Processing parameters
SI                     65536
SF               400.3000091 MHz
WDW                       EM
SSB       0
LB                      0.30 Hz
GB        0
PC                      1.00
```

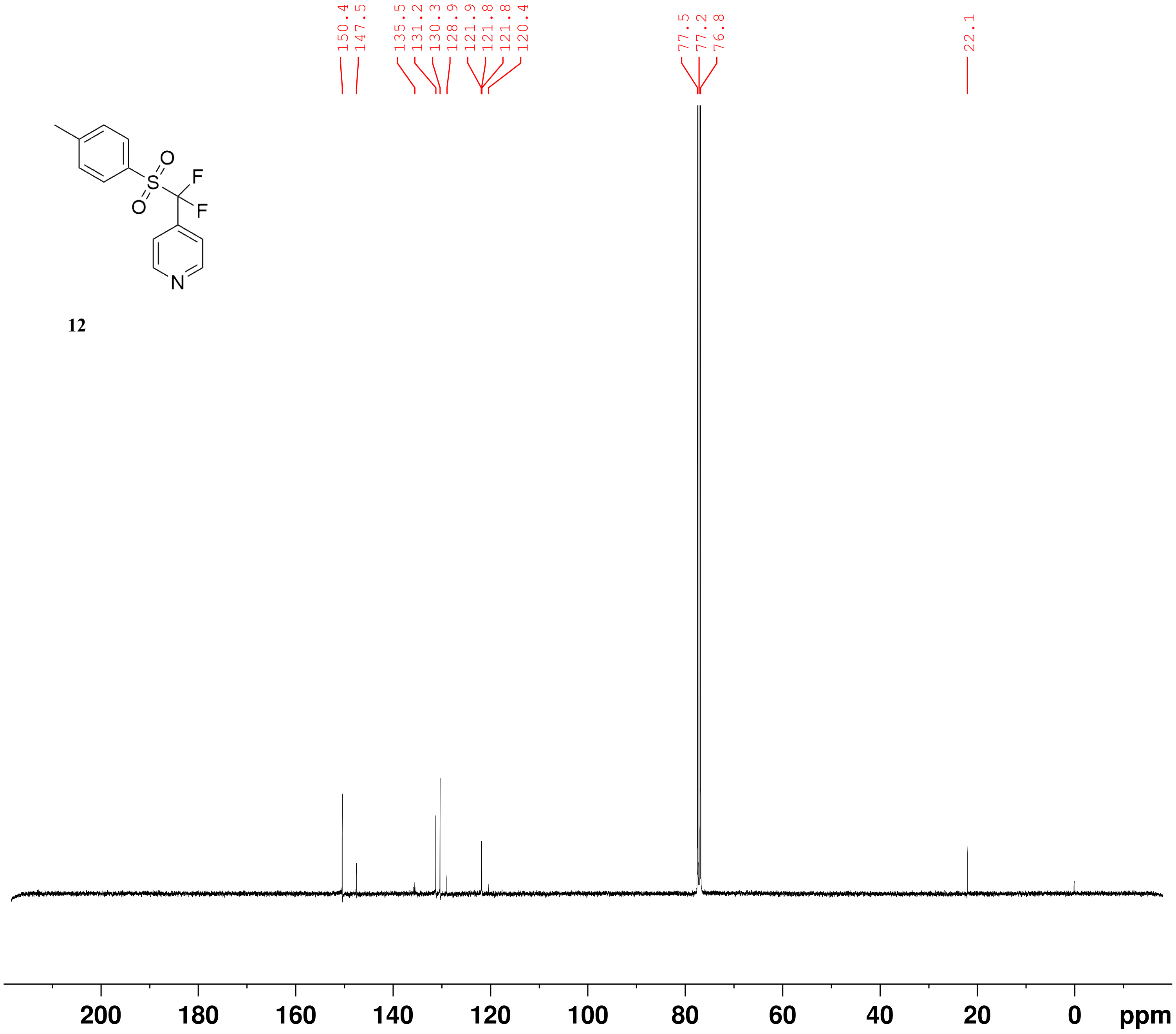


```
Current Data Parameters
NAME       13C_ST-8-43-real
EXPNO                     2
PROCNO                    1

F2 - Acquisition Parameters
Date_              20220926
Time                  14.11 h
INSTRUM              Avance
PROBHD     Z167430_0032 (
PULPROG              zgpg30
TD                    65536
SOLVENT               CDCl3
NS                      200
DS                        4
SWH               23809.523 Hz
FIDRES             0.726609 Hz
AQ                1.3762560 sec
RG                     3.25
DW                   21.000 usec
DE                    19.29 usec
TE                    298.0 K
D1               3.00000000 sec
D11              0.03000000 sec
TD0                       1
SFO1            100.6655806 MHz
NUC1                    13C
P0                     3.33 usec
P1                    10.00 usec
PLW1            39.31399918 W
SFO2            400.3016012 MHz
NUC2                     1H
CPDPRG[2            waltz64
PCPD2                 80.00 usec
PLW2             8.80000019 W
PLW12            0.20176961 W
PLW13            0.10112690 W

F2 - Processing parameters
SI                   131072
SF              100.6555019 MHz
WDW                      EM
SSB        0
LB                     1.00 Hz
GB         0
PC                     1.40
```

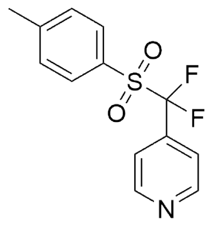

12

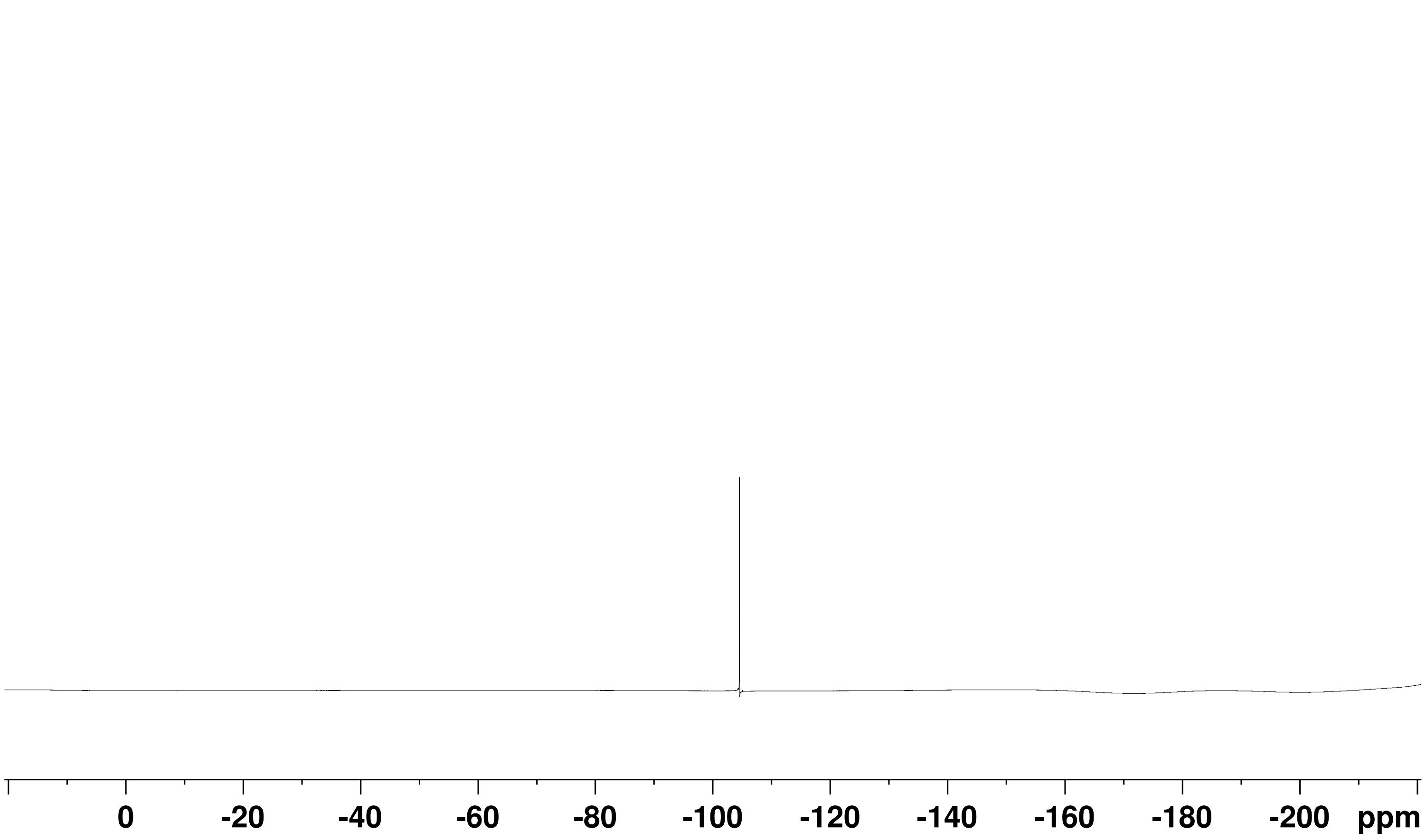

Current Data Parameters	
NAME	19FST0843
EXPNO	1
PROCNO	1
F2 - Acquisition Parameters	
Date_	20220901
Time	18.12 h
INSTRUM	Avance
PROBHD	Z167430_0032 (
PULPROG	zg30
TD	131072
SOLVENT	CDCl3
NS	16
DS	4
SWH	90909.094 Hz
FIDRES	1.387163 Hz
AQ	0.7208960 sec
RG	29.1159
DW	5.500 usec
DE	18.00 usec
TE	298.0 K
D1	1.00000000 sec
TD0	1
SFO1	376.6206602 MHz
NUC1	19F
P0	5.00 usec
P1	15.00 usec
PLW1	6.10930014 W
F2 - Processing parameters	
SI	32768
SF	376.6583260 MHz
WDW	EM
SSB	0
LB	2.00 Hz
GB	0
PC	1.00

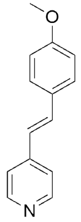

13

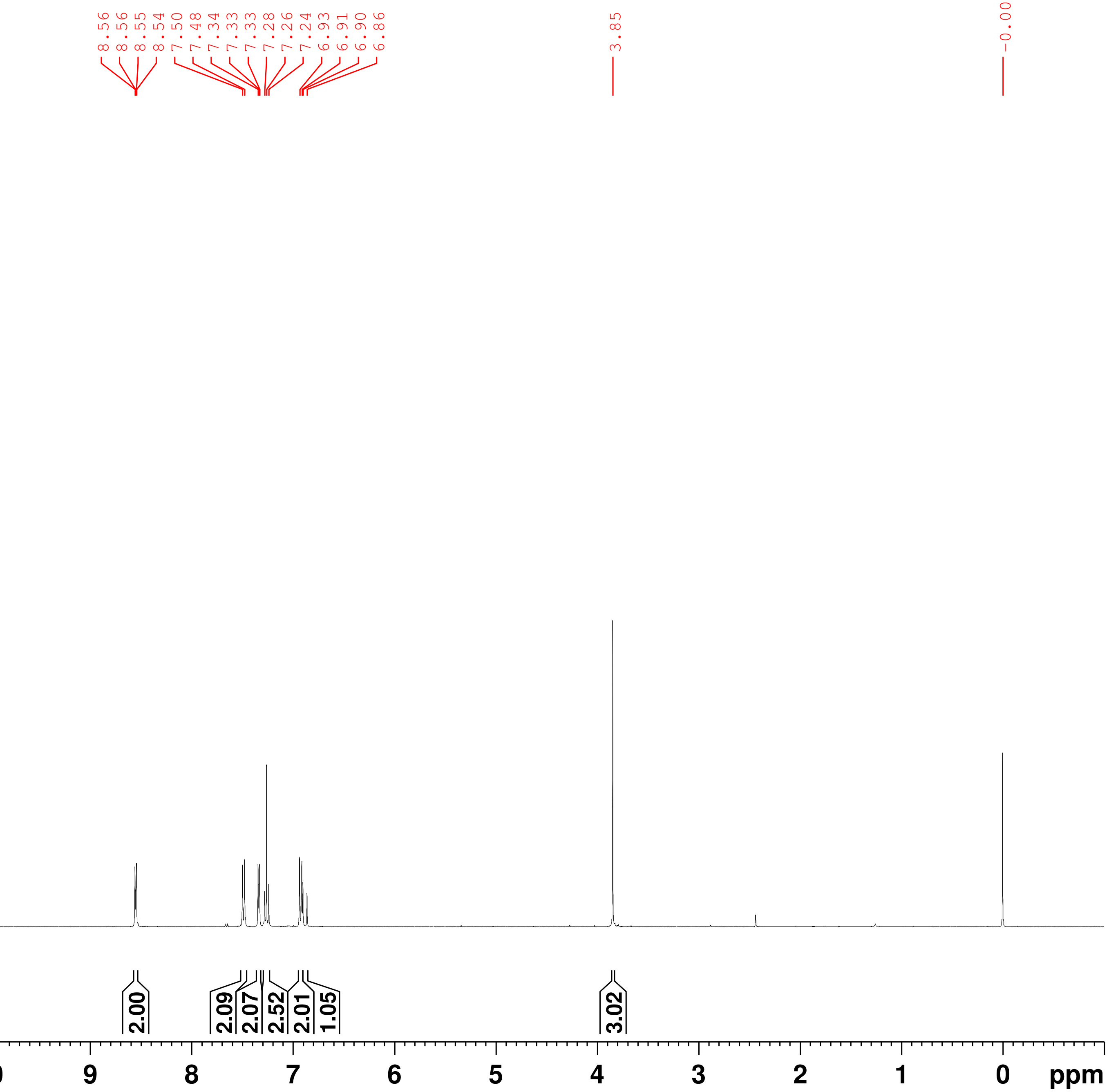


```
Current Data Parameters
NAME        1H_ST-7-289-again
EXPNO                       2
PROCNO                      1

F2 - Acquisition Parameters
Date_                20221027
Time                   9.36 h
INSTRUM                Avance
PROBHD       Z167430_0032 (
PULPROG                  zg30
TD                      65536
SOLVENT                 CDCl3
NS                         16
DS                          0
SWH                  8196.722 Hz
FIDRES               0.250144 Hz
AQ                  3.9976959 sec
RG                        101
DW                     61.000 usec
DE                      13.20 usec
TE                      298.0 K
D1                 0.10000000 sec
TD0                         1
SFO1              400.3024719 MHz
NUC1                       1H
P0                       4.00 usec
P1                      12.00 usec
PLW1               8.80000019 W

F2 - Processing parameters
SI                      65536
SF                400.3000096 MHz
WDW                        EM
SSB          0
LB                       0.30 Hz
GB           0
PC                       1.00
```

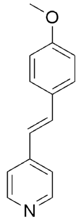

13

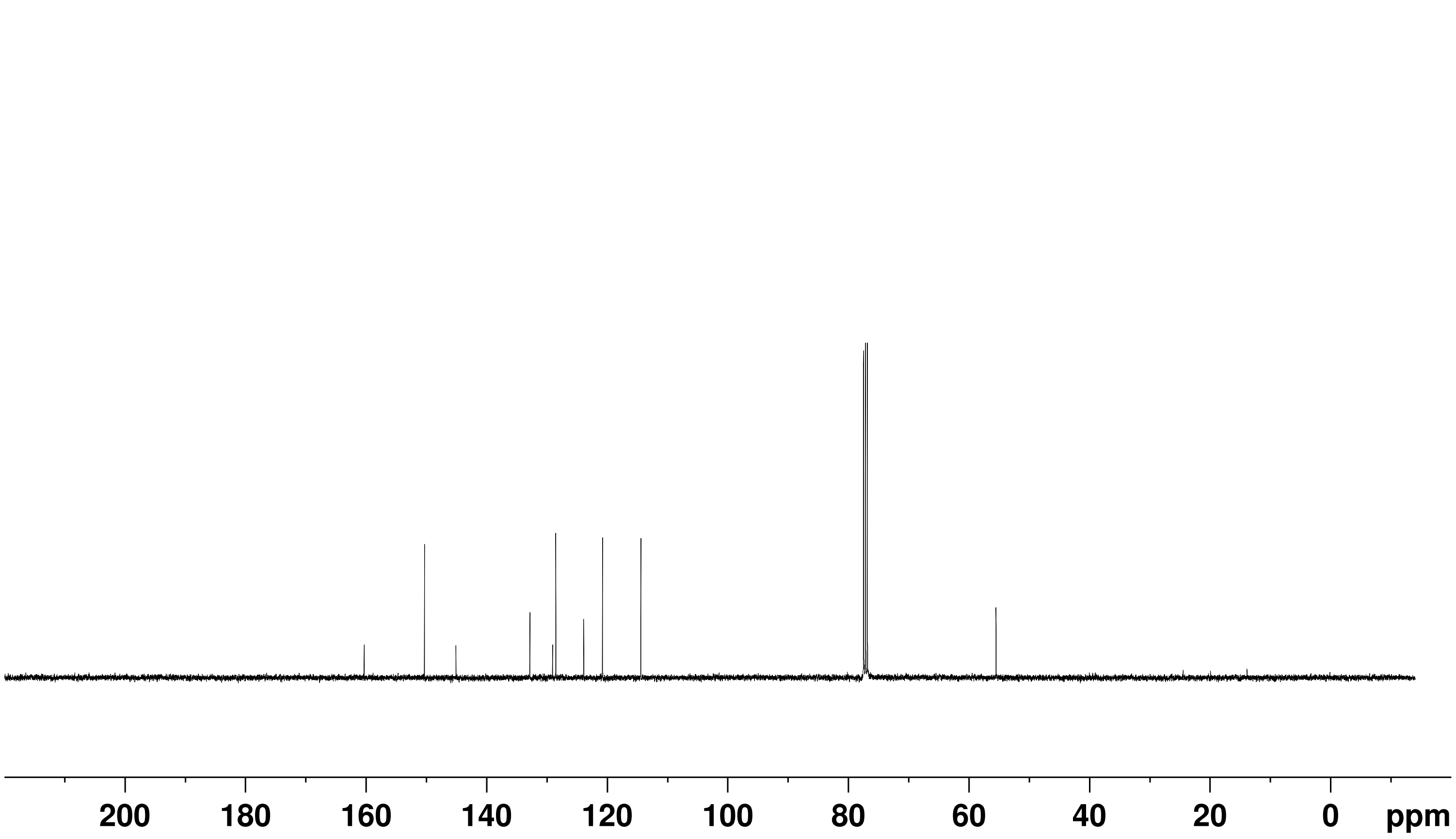

Current Data Parameters
NAME 13C_ST-7-289
EXPNO 1
PROCNO 1

F2 - Acquisition Parameters
Date_ 20221026
Time 18.34 h
INSTRUM Avance
PROBHD Z167430_0032 (
PULPROG zgpg30
TD 65536
SOLVENT CDCl3
NS 32
DS 4
SWH 25000.000 Hz
FIDRES 0.762939 Hz
AQ 1.3107200 sec
RG 3.25
DW 20.000 usec
DE 18.29 usec
TE 298.0 K
D1 2.00000000 sec
D11 0.03000000 sec
TD0 1
SFO1 100.6665872 MHz
NUC1 13C
P0 3.33 usec
P1 10.00 usec
PLW1 39.31399918 W
SFO2 400.3016012 MHz
NUC2 1H
CPDPRG[2 waltz64
PCPD2 80.00 usec
PLW2 8.80000019 W
PLW12 0.20176961 W
PLW13 0.10112690 W

F2 - Processing parameters
SI 131072
SF 100.6555038 MHz
WDW EM
SSB 0
LB 1.00 Hz
GB 0
PC 1.40

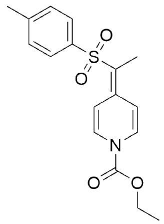

14

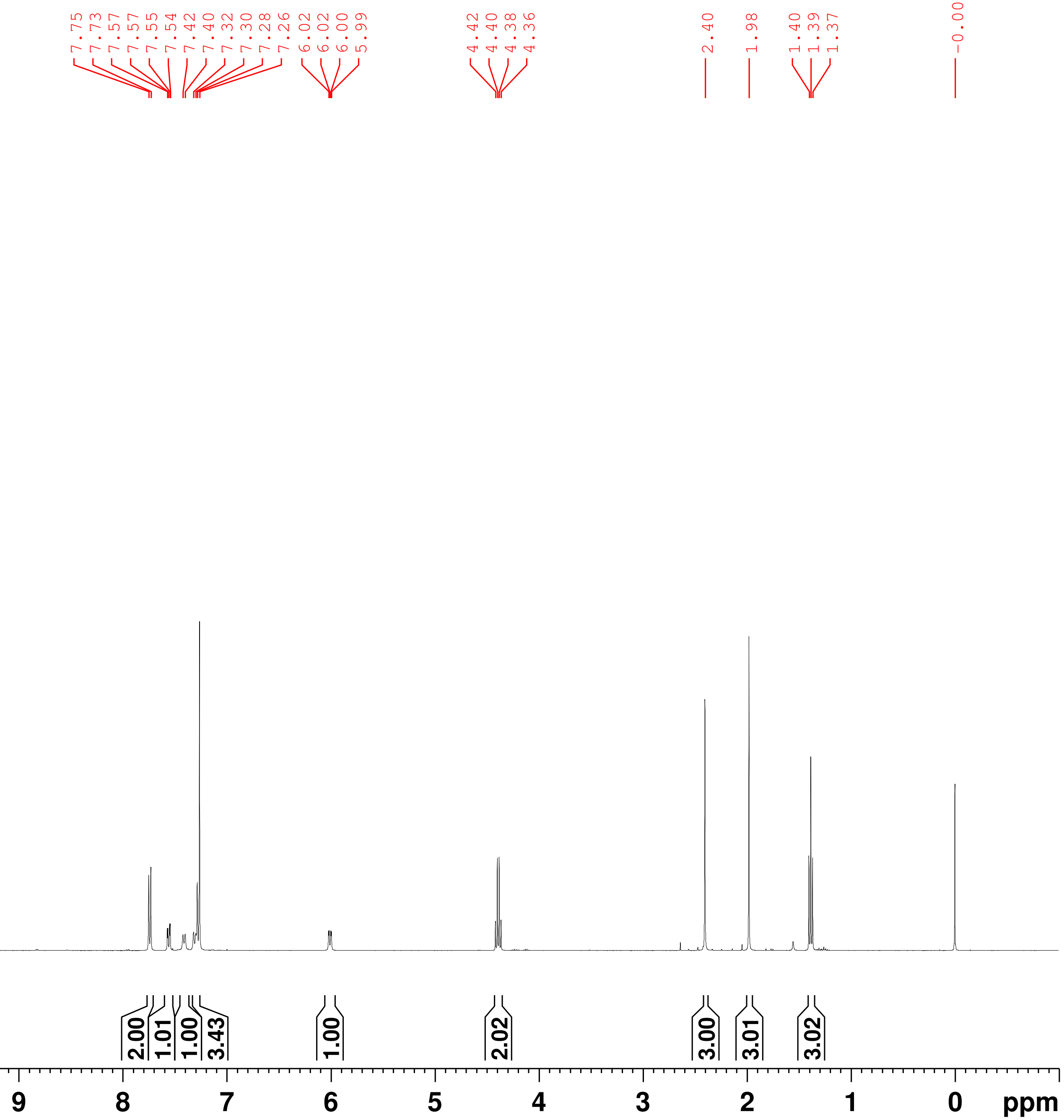

Current Data Parameters
NAME 1H_JH-1-67
EXPNO 2
PROCNO 1

F2 - Acquisition Parameters
Date_ 20220725
Time 11.12 h
INSTRUM Avance
PROBHD Z167430_0032 (
PULPROG zg30
TD 65536
SOLVENT CDCl3
NS 16
DS 0
SWH 8196.722 Hz
FIDRES 0.250144 Hz
AQ 3.9976959 sec
RG 101
DW 61.000 usec
DE 13.20 usec
TE 298.0 K
D1 0.10000000 sec
TD0 1
SFO1 400.3024719 MHz
NUC1 1H
P0 4.00 usec
P1 12.00 usec
PLW1 8.80000019 W

F2 - Processing parameters
SI 65536
SF 400.3000091 MHz
WDW EM
SSB 0
LB 0.30 Hz
GB 0
PC 1.00

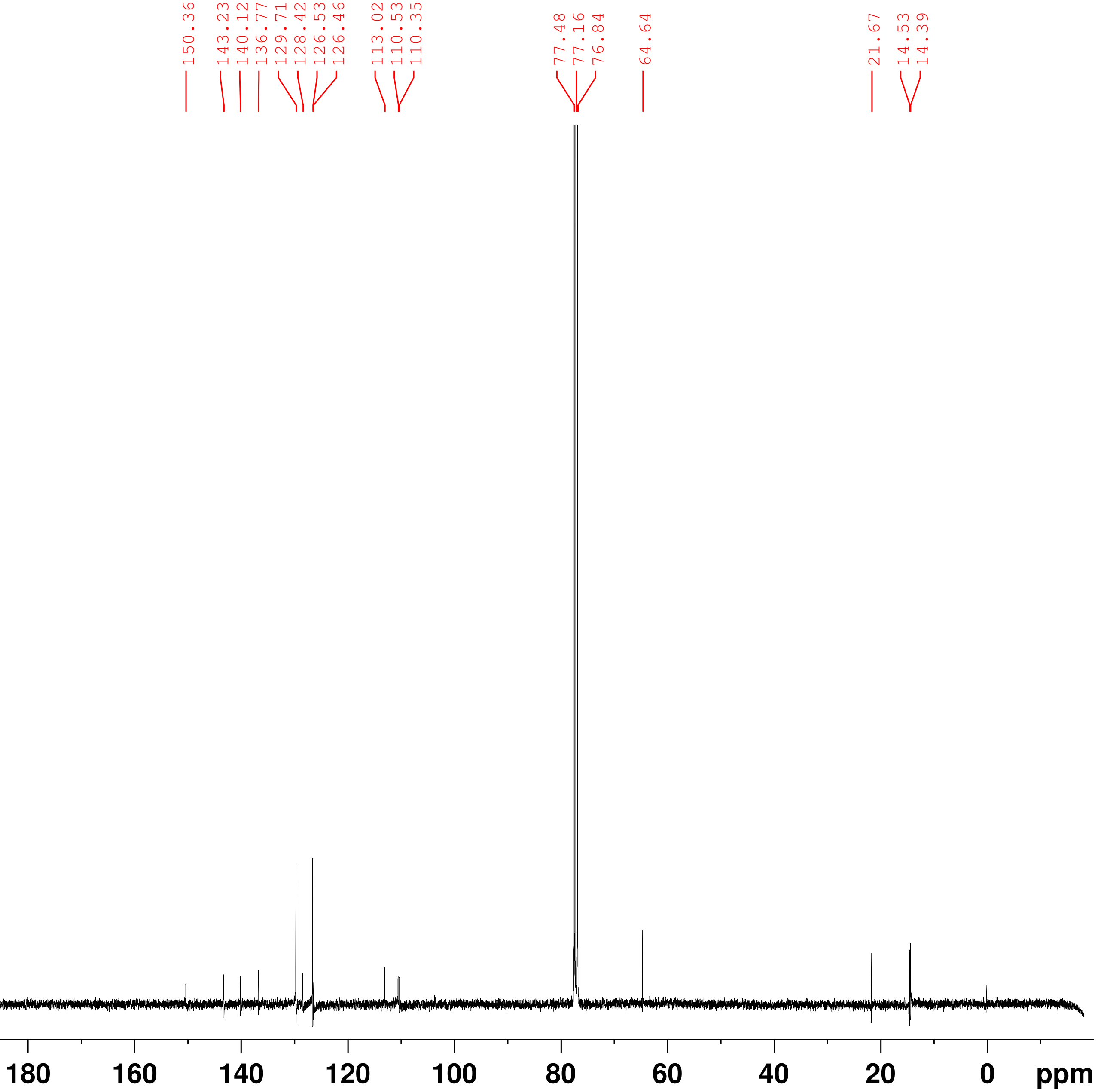

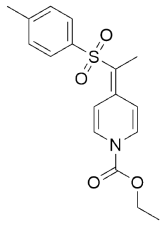

14

Current Data Parameters
NAME 13C_JH-1-67-re
EXPNO 3
PROCNO 1

F2 - Acquisition Parameters
Date_ 20220725
Time 11.32 h
INSTRUM Avance
PROBHD Z167430_0032 (
PULPROG zgpg30
TD 65536
SOLVENT CDCl3
NS 256
DS 4
SWH 23809.523 Hz
FIDRES 0.726609 Hz
AQ 1.3762560 sec
RG 3.25
DW 21.000 usec
DE 19.29 usec
TE 298.0 K
D1 2.00000000 sec
D11 0.03000000 sec
TD0 1
SFO1 100.6655806 MHz
NUC1 13C
P0 3.33 usec
P1 10.00 usec
PLW1 39.31399918 W
SFO2 400.3016012 MHz
NUC2 1H
CPDPRG[2 waltz64
PCPD2 80.00 usec
PLW2 8.80000019 W
PLW12 0.20176961 W
PLW13 0.10112690 W

F2 - Processing parameters
SI 131072
SF 100.6555018 MHz
WDW EM
SSB 0
LB 1.00 Hz
GB 0
PC 1.40
